# Redox cycling nitroxide limits cellular iron availability and selectively inhibits iron-sulfur cluster metabolism

**DOI:** 10.1038/s41420-026-03042-w

**Published:** 2026-03-24

**Authors:** Erdem M. Terzi, Kenji M. Fujihara, Marte Molenaars, Douglas E. Biancur, Richard Possemato

**Affiliations:** Department of Pathology, New York University Grossman School of Medicine, New York, NY 10016, USA. Laura & Isaac Perlmutter Cancer Center, New York, NY USA

**Keywords:** Nutrient signalling, Metabolic pathways

## Abstract

Iron-sulfur clusters (ISCs) are redox active cofactors for essential proteins with diverse functions. We demonstrate that Tempol, a redox cycling nitroxide, limits iron bioavailability in a manner distinct from iron chelators, mainly via its effect on ascorbate and iron redox balance. This non-canonical iron limitation triggers upregulation of IRP2 and HIF1α, proteins whose degradation is ferrous iron-dependent, while disrupting ISC synthesis only in a subset of cell lines. Suppression of ISC synthesis inhibits ISC-dependent enzymes, destabilizes ISC proteins, and reduces cell viability, particularly in cells dependent on ISC protein ACO2. These effects are reversed by the reducing agent ascorbate, a cofactor required for multiple enzymes, such as the HIF1α prolyl hydroxylases. Tempol treatment also inhibits ferroptosis, an oxidative form of cell death catalyzed by reduced iron. These results demonstrate ascorbate and cellular iron redox state are essential in iron homeostasis, which is proposed to underlie pathological conditions from neurodegeneration to cancer.

## Introduction

Iron is an essential micronutrient required for several vital functions, such as oxidative phosphorylation, DNA replication and repair, epigenetic regulation, and oxygen transport and storage [1]. Iron is predominantly utilized either as a free ion or in an iron-containing cofactor such as iron-sulfur clusters (ISC) and heme [2].

Regulation of the redox-active nature of iron is important for maintaining these functions. Enzymes such as those in the iron- and alpha-ketoglutarate (αKG)-dependent dioxygenase family utilize ascorbate (Vitamin C) as a co-factor likely to restore iron to its reduced, ferrous (Fe^2+^) form [3]. On the other hand, excess Fe^2+^ can also facilitate oxidative damage and promote cell death via ferroptosis, an iron-dependent cell death mechanism characterized by membrane lipid peroxidation [4–6].

Iron dysregulation is reported in diverse pathological contexts: iron overload causes disease pathology in hemochromatosis, brain iron accumulation is associated with multiple forms of neurodegeneration, and increased ISC synthesis is observed in lung cancer [1, 7–10]. Moreover, highly proliferative cells require increased iron uptake via transferrin receptor (TFR1) [11, 12]. In contrast, iron is sequestered in response to infection. For example, hepcidin produced by the liver limits dietary iron uptake and its release from macrophages [13, 14].

**Fig. 1:**
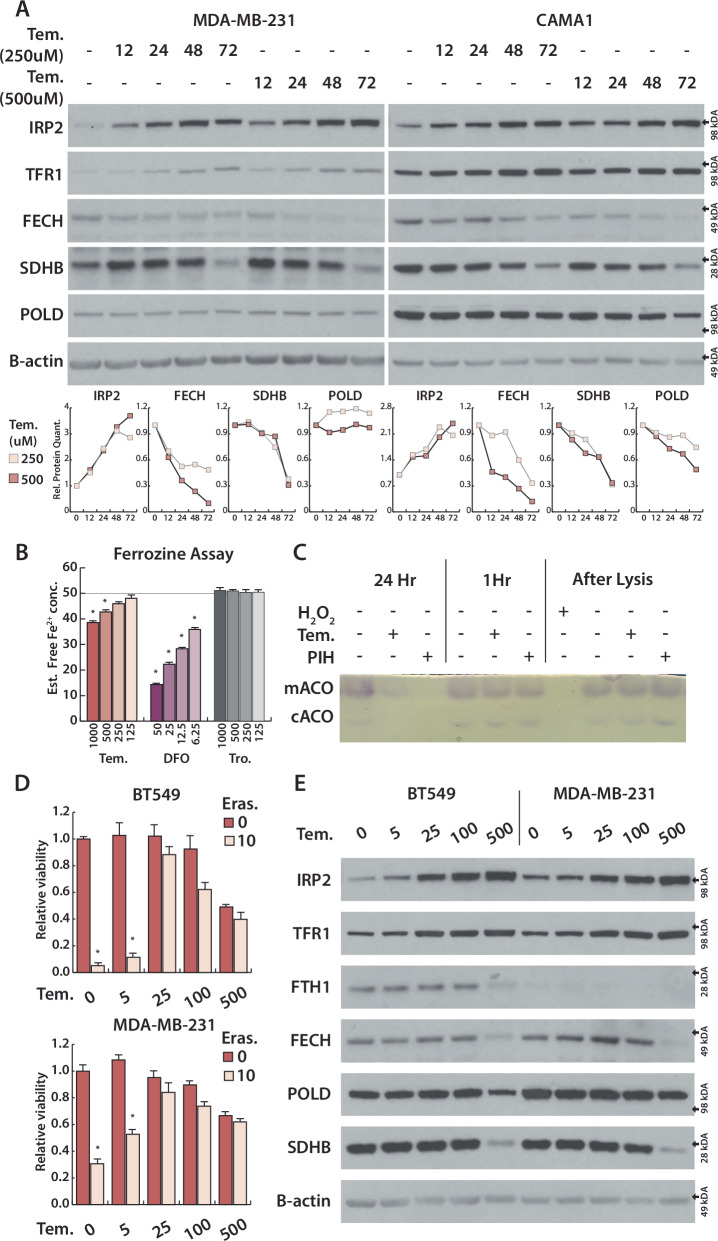
Nitroxide compound Tempol limits cellular iron, degrades ISC proteins and suppress ferroptosis. **A** Above, Immunoblots from lysates derived from indicated cell lines, treated with Tempol (250, 500 μM) for 12, 24, 48, 72 h for indicated proteins. Below, Quantification of immunoblots. **B** Estimated free Fe^2+^ iron after incubation of 50 μM ammonium iron(II) sulfate with Tem (125, 250, 500, 1000 μM), Deferoxamine (DFO, 6.25, 12.5, 25, 50 μM) and Trolox (Tro., 125, 250, 500, 1000 μM) for 5 min. **C** First 6 lanes, aconitase assay of HEPG2 cells, treated with Tem (500 μM) and PIH (50 μM) for 1 h and 24 h. Last 4 lane, aconitase assay of HEPG2 cell lysates, incubated with 2.5 mM Tempol, 250 μM PIH and 1 mM H_2_O_2_ for 20 min. **D** Relative viability of BT549 and MDA-MB-231, treated with Tempol (5, 25, 100, 500) or Eras (10 μM) for 3 days. **E** Immunoblots from lysates derived from BT549 and MDA-MB-231, treated with Tempol (5, 25, 100, 500 μM, 3 days) for indicated proteins *p < 0.01, error bars are s.e.m.

In mammals, the cellular bioavailable labile iron pool (LIP) is tightly regulated by the iron-responsive element-binding proteins, IRP1 and IRP2 [15]. Either low cellular iron or low ISC levels activates IRPs, promoting their binding to iron-response elements (IRE) in target mRNAs [16–18]. IRP:IRE binding in turn regulates a set of iron metabolism genes involved in iron import, export, storage, and utilization to restore iron homeostasis (Supplementary Fig. S1A). Based on the location of the IRE in the target mRNA, IRP-mRNA binding either stabilizes and upregulates the target mRNA, such as in TFR1 [19], or inhibits translation of the target mRNA without decreasing transcript level, such as in the iron storage proteins ferritin heavy and light chains (FTH1 and FTL) (Fig. 1A) [20, 21]. IRP1 and IRP2 are homologous proteins and have largely overlapping targets, but are activated in different ways. The cytosolic aconitase enzyme contains an ISC that can be lost upon oxidative damage or iron restriction, and the resulting apo-protein—termed IRP1—binds IREs [22]. In contrast, IRP2 is continuously degraded by a ubiquitin ligase complex containing an iron-regulated protein, FBXL5. Iron limitation results in ubiquitin-proteasome mediated FBXL5 degradation, thereby stabilizing IRP2 [23, 24]. Moreover, recent studies demonstrate that IRP2 is regulated by ISC levels [17, 18]. These differences can partially explain distinct phenotypes observed in mice with deletion of *Aco1 or Ireb2*, the genes encoding IRP1 and IRP2 [25]. Deletion of both *Aco1 and Ireb2* is embryonic lethal [26], indicating that IRP1 and IRP2 partially compensate for each other.

*Ireb2* knockout mice and patients with mutations in *IREB2* display neurological phenotypes and microcytic anemia due to iron dysregulation [27–30]. Tempol, a redox cycling nitroxide, mitigates neuromuscular impairment associated with IRP2 deficiency in mice, a function attributed to the ability of Tempol to selectively activate IRP1 by degrading its ISC [31, 32]. Contrary to its proposed role in ISC oxidation, Tempol has also been hypothesized to limit pathology in multiple disease models due to its antioxidant properties. Indeed, Tempol improves neuronal phenotypes in an ALS mouse model with mutated superoxide dismutase 1 [33], limits hepatotoxicity caused by acetaminophen overdose [34], and acts as a chemo-preventative agent in ATM-deficient mice [35]. Tempol has also recently been reported to target the ISC in the SARS-CoV-2 RNA-dependent RNA polymerase (RdRp) and helicase, proteins required for virus replication [36, 37]. Although Tempol treatment failed to produce a significant improvement in COVID-19 patients in Phase II/III trials (NCT04729595), Tempol has therapeutic potential owing to its relative safety and ability to target ISCs in enzymes required for viral replication.

Here, we show that Tempol counters the effects of ascorbate in human cells, thereby limiting the labile iron pool dependent on iron redox state, in a distinct manner from iron chelators. Accordingly, non-canonical iron limitation by Tempol upregulates IRP2 and HIF1α and inhibits ferroptosis. Utilizing a cell line panel, we demonstrate that redox-dependent iron limitation by Tempol differentially affects the ability of cells to maintain ISC metabolism, limiting their proliferative capacity. Altogether, our model emphasizes the importance of ascorbate and iron redox in cellular iron metabolism, which can be altered by redox active molecules such as Tempol.

## Results

### Nitroxide compound Tempol activates IRP2 and degrades ISC proteins independent of IRP1

Prior work indicates that the nitroxide compound Tempol has a unique ability to selectively degrade the ISC in the cytoplasmic aconitase enzyme, resulting in activation of its IRP1 form, a property that could provide insight into cellular iron metabolism [32]. Indeed, upon Tempol treatment we observe upregulation of TFR1 mRNA and protein and downregulation of FTH1/FTL protein in accordance with IRP1 activation (Supplementary Figs. S1A–C, S2A). Treatment with either Tempol or iron chelator deferoxamine (DFO) inhibits cytosolic aconitase activity and mitochondrial aconitase activity (encoded by the gene *ACO2*) (Supplementary Fig. S2B). Unexpectedly, tempol also causes a dose-dependent increase in IRP2 protein levels in most cell lines (Supplementary Fig. S1A). We found this observation counterintuitive given that activation of IRP1 and its downstream targets should increase iron availability and suppress IRP2.

To assess whether Tempol has effects on iron metabolism independent of its proposed function to activate IRP1, we generated cell lines in which the IRP1 gene (*ACO1*) is disrupted by CRISPR/Cas9 (Supplementary Fig. S3A, B). Indeed, Tempol upregulated IRP2 protein levels in cells lacking IRP1 to a similar extent as IRP1 wild type cell lines, consistent with an independent effect on iron homeostasis (Supplementary Fig. S3A, B). As most ISC proteins destabilize upon losing their ISC [38], we asked whether Tempol treatment could result in the loss of other ISC-containing proteins. We measured the level of a set of proteins with diverse functions - ferrochelatase (FECH) [39], succinate dehydrogenase (SDHB) [40], and DNA polymerase delta (POLD) [41] - and compared the kinetics of IRP2 protein accumulation to the levels of these ISC proteins after Tempol treatment. We observed IRP2 accumulation starting at 12 h, followed by differential loss of ISC proteins at later time points, in both control cells or those lacking IRP1 (Fig. 1A, Supplementary Fig. S3A, B). Acute inhibition of ISC synthesis via doxycycline (DOX)-inducible expression of a dominant negative ISCU (ISCU-DN) [42] resulted in an increase in IRP2 and decrease in FECH levels, similar to treatment with Tempol or DFO (Supplementary Fig. S3B, C). Overall, our data suggests that iron limitation and subsequent IRP2 activation occur prior to ISC inhibition, and Tempol can alter iron and ISC metabolism similar to iron chelator DFO.

### Iron limitation by Tempol indirectly inhibits ISC synthesis and suppresses ferroptosis

Decreasing iron availability, direct ISC degradation, and inhibition of ISC biosynthesis can each behave similarly in the context of IRP activation and ISC metabolism. Therefore, to independently assess the effect of Tempol on these possible mechanisms, we measured whether Tempol can limit iron availability and ISC protein activity in vitro. Incubation with the antioxidant Trolox does not decrease available Fe^2+^ levels, while DFO efficiently limits Fe^2+^ availability. Tempol lowers Fe^2+^ availability, but to a significantly lesser degree than DFO (Fig. 1B). Tempol is quite cell permeable [43, 44], enabling it to alter intracellular iron levels more efficiently than DFO, which has low cell permeability. Therefore, we measured the effect of Tempol and cell permeable iron chelator PIH on ISC-dependent aconitase activity. Both Tempol and cell permeable iron chelator PIH decrease ISC-dependent aconitase activity significantly after 24 h treatment, but not after 1 h treatment (Fig. 1C). To measure direct inhibition of ISC by Tempol, we incubated cell lysates without addition of reducing agents with very high doses of Tempol, PIH, or H_2_O_2_ for 20 min. While H_2_O_2_ treatment eliminates aconitase activity by directly destroying ISC, neither Tempol nor PIH decrease aconitase activity on this time scale (Fig. 1C). These data are consistent with indirect aconitase inhibition by Tempol via iron limitation and subsequent inhibition of ISC biosynthesis.

Free Fe^2+^ facilitates lipid peroxidation [45]. As such, another effect of increasing cellular ferrous iron availability is sensitization to death by ferroptosis [46]. Indeed, we have shown that ISC inhibition activates IRP1 and IRP2, increasing ferroptosis sensitivity [18]. To directly activate IRP2 and increase iron availability, we engineered cells in which the protein responsible for IRP2 degradation, FBXL5, is placed under DOX control. As anticipated, DOX addition suppresses FBXL5, increases IRP2 and TFR1, and decreases FTL, similar to Tempol treatment (Supplementary Fig. S4A). However, while FBXL5 suppression sensitizes cells to ferroptosis, Tempol treatment blocks ferroptosis in these conditions, further indicating that Tempol limits cellular iron availability rather than directly degrading ISCs (Supplementary Fig. S4B). We also measured the dose-dependent effect of Tempol on ferroptosis sensitivity and IRP2 activation to determine whether iron limitation correlates with ferroptosis suppression. Tempol treatment concomitantly increases IRP2 levels and protects cells from ferroptosis beginning at 25 μM; however, ISC protein stability is unaffected until 500 μM at these time points (Fig. 1D, E). We observe a similar strong correlation with ferroptosis inhibition and IRP2 activation in iron chelator DFO treated cells (Supplementary Fig. S4C, D). Thus, we conclude that Tempol limits cellular Fe^2+^ availability, upregulates IRP2, and suppresses ferroptosis at low doses, an effect which is sufficient to inhibit ISC synthesis at higher concentrations or longer time points.

#### Tempol-dependent iron limitation is rescued by ascorbate, but not by iron supplementation

**Fig. 2:**
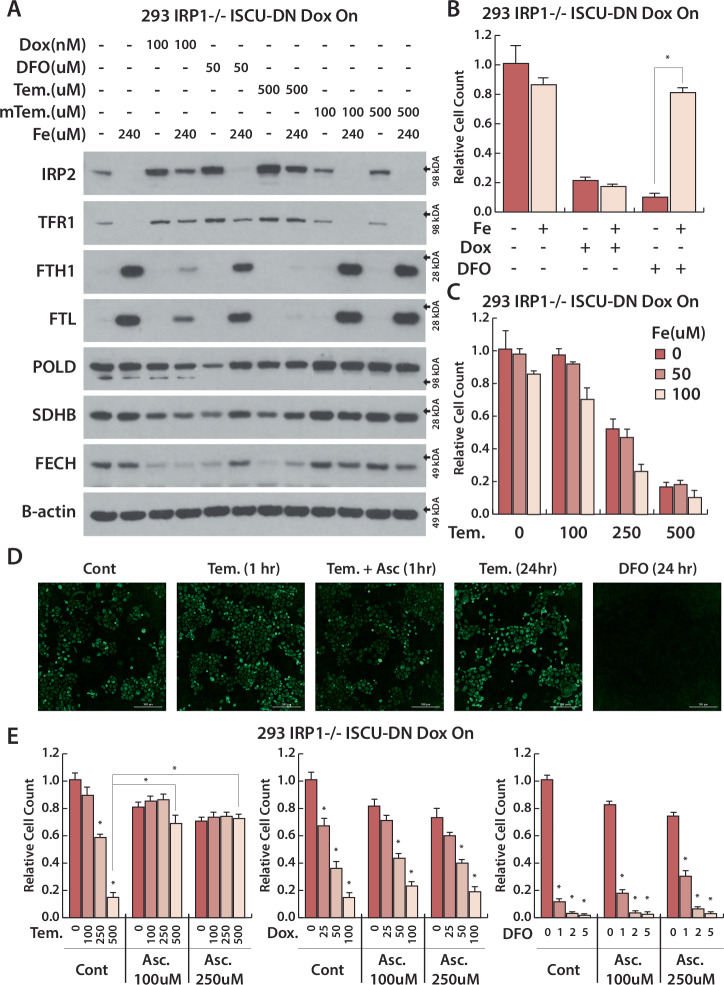
Tempol limits alters iron redox balance and limit cellular iron by a distinct mechanism than iron chelation. **A** Immunoblots for indicated proteins of lysates derived from IRP1 knockout HEK293 cell line expressing doxycycline (Dox)-inducible ISCU-DN, treated with Dox (100 nM), Deferoxamine (DFO, 50 μM), Tempol (Tem., 500 μM), mitoTempo (mTem., 100, 500 μM) for 2 days, Iron(III) nitrate (Fe, 240 μM) added after day 1. **B** Relative cell count of IRP1 knockout HEK293 cell line expressing Dox-inducible ISCU-DN, treated with Dox (100 nM), DFO (10 μM) and Fe (100 μM) for 3 days. **C** Relative cell count of IRP1 knockout HEK293 cell line expressing Dox-inducible ISCU-DN, treated with Tem. (100, 250, 500 μM) and Fe (50, 100 μM) for 4 days. **D** Mitochondrial Fe^2+^ iron levels of 647 V cells treated with 500 μM Tempol, 125 μM Ascorbate or 10 μM DFO for 1 h and 24 h. **E** Relative cell count of IRP1 knockout HEK293 cell line expressing Dox-inducible ISCU-DN, treated with Tem. (100, 250, 500 μM), Dox (25, 50, 100 nM), DFO (1, 2, 5 μM) and Iron(III) nitrate (Fe, 50 μM) for 4 days. *p < 0.01, error bars are s.e.m.

Based on these data, we next considered whether Tempol could be acting as a weak iron chelator. As iron chelators directly bind iron, iron supplementation reverses their effect by saturating their iron binding capacity. In contrast, iron supplementation should not rescue the effect of direct ISC synthesis inhibition. Accordingly, we observed that iron supplementation completely reverses IRP2 stabilization and rescues ISC protein levels in DFO treated cells, while iron supplementation minimally affects IRP2 stabilization and ISC protein level loss upon ISC synthesis inhibition (DOX-inducible ISCU-DN) (Fig. 2A). Similarly, iron supplementation is able to completely rescue proliferation defects induced by iron chelation, but not ISC inhibition (Fig. 2B). Upon Tempol treatment, iron supplementation only partially affects the levels of ISC proteins and does not rescue proliferation (Fig. 2A–C, Supplementary Fig. S5). As incorporation of iron into ISCs occurs within mitochondria, the mitochondrial Fe^2+^ pool is important for ISC homeostasis. While, incubation with DFO completely eliminates mitochondrial Fe^2+^, Tempol does not decrease mitochondrial Fe^2+^ (Fig. 2D). Therefore, we conclude that Tempol limits cellular iron availability in a mechanism distinct from iron chelators. Tempol limits iron availability with significantly lower ratio compared to iron chelator DFO in vitro assay (Fig. 1B), Although intracellular labile iron pool is undoubtedly much lower reported to be around 5 μM compared to in vitro assay [47, 48], we next investigated whether Tempol inhibit bioavailable iron indirectly, related to a factor or factors in the intracellular environment. Fe^2+^-dependent enzymes can utilize ascorbate, a redox active cofactor, thought to act as a reducing agent to recycle Fe^3+^ to Fe^2+^. Thus, we hypothesized that Tempol might limit cellular iron by interfering in this process, promoting an environment favoring Fe^3+^. Remarkably, ascorbate supplementation completely rescues cell growth and mitochondrial iron levels in Tempol treated cells, while ascorbate does not affect proliferation defects induced by iron chelation or inhibition of ISC synthesis (Fig. 2E). TEMPOL’s reduction rate by ascorbate is often used as a proxy for cellular reducing capacity or ascorbate content [49, 50]. Taken together, our data indicates that Tempol limits iron availability in a different manner than iron chelators, potentially via interfering ascorbate and altering the iron redox state.

### Redox-dependent iron limitation inhibits growth selectively by inhibiting ISC synthesis

**Fig. 3:**
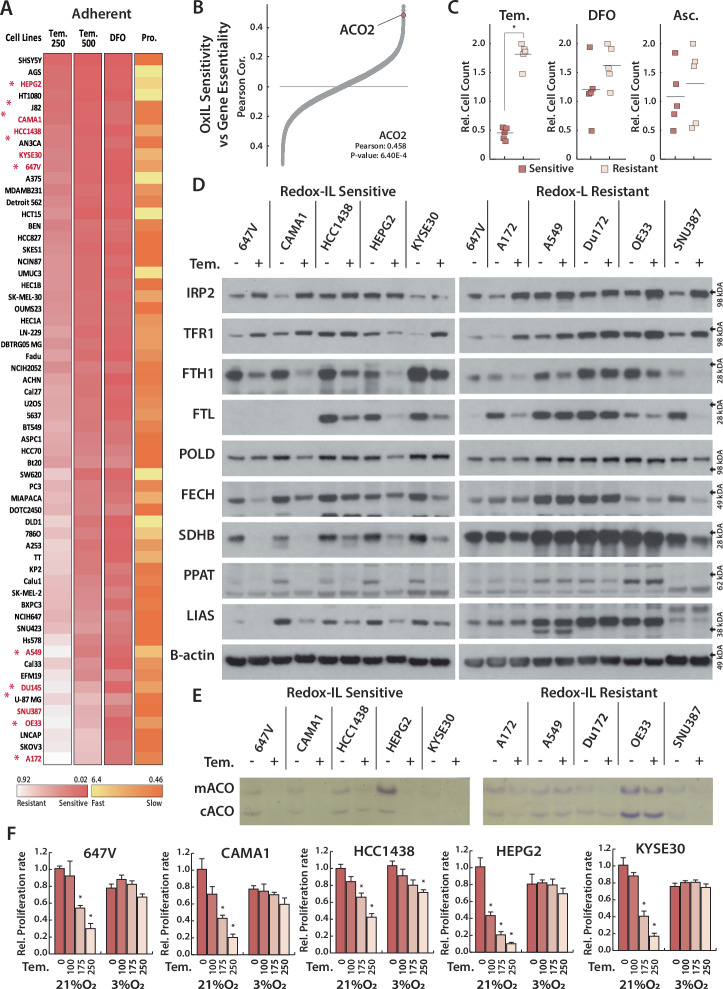
Redox-dependent iron limitation decreases ISC protein levels in sensitive, but not in resistant cell lines. **A** Heatmap for relative proliferation rate (Pro.) and the relative cell count of 60 cell lines, treated with Deferoxamine (DFO, 10 μM), Tempol (Tem. 250, 500 μM) for 4 days. Proliferation rate is measured as cell count relative to median cell line. **B** Correlation between gene essentiality and sensitivity to redox-dependent iron limitation; Gene essentiality date is obtained from DepMap databases for CRISPR. **C** Relative cell count for 5 sensitive and 5 resistant cell lines from (**A**) (red *), treated with Tem. (250, 500 μM), DFO (1, 2, 5 μM), Ascorbate (Asc., 100 250, 500 μM) for 4 days. **D** Immunoblots for indicated proteins of lysates derived from 5 sensitive and 5 resistant cell lines from (**A**) (red *), treated with Tem. (250 μM) for 4 days. **E** Aconitase assay for mitochondrial(mACO) and cytosolic(cACO) aconitases of 5 sensitive and 5 resistant cell lines from (**A**) (red *), treated with Tem. (250 μM) for 4 days. **F** Relative cell count for 5 sensitive cell lines, treated with Tem. (100, 175, 250 μM), incubated at 21% and 3% O_2_ for 4 days. *p < 0.01, error bars are s.e.m.

In the panel of cell lines tested above, we noted a variable response of redox-dependent iron limitation on cell viability. Cancer cell lines have inherent genetic and epigenetic variabilities, and their differential response to pharmacological agents can reveal important information about underlying biology. Therefore, we measured the proliferation rate of 60 adherent and 15 suspension cell lines after a 4-day treatment with 250 μM or 500 μM Tempol or 10 μM DFO (Fig. 3A, Supplementary Fig. S6A). We observed a highly variable response to Tempol treatment across lines, which did not correlate with proliferation rate and only weakly correlated with DFO sensitivity (Supplementary Fig. S6B,C). Comparing gene essentiality data from the DepMap dataset, we observe a significant positive correlation between mitochondrial aconitase (ACO2) essentiality and Tempol sensitivity (Fig. 3B, Supplementary Fig. S7). As our data show that Tempol inhibits ISC proteins, including ACO2, differentially across cell lines, we hypothesized that the observed pattern of Tempol sensitivity can be based on selective ISC degradation broadly, and specific effects on ACO2 in cells with a heightened dependency on this protein. Those cell lines that are sensitive to TEMPOL treatment but are relatively resistant to ACO2 inhibition do not exhibit a selective dependency on any other ISC-containing proteins (Supplementary Table), consistent with ACO2 dependence being the major single driver of TEMPOL sensitivity.

To investigate the mechanism of sensitivity to redox-dependent iron limitation, we selected 5 Tempol sensitive and 5 resistant adherent cell lines, attempting to match their lineage and proliferation rates. We repeated our assessment of sensitivity to iron chelation at a lower DFO concentration and verified that there is no strong correlation with sensitivity to Tempol (Fig. 3C, Supplementary Fig. S8A). Ascorbate is toxic at elevated concentrations due to its reducing potential; however, we also observe no correlation between sensitivity to ascorbate and Tempol (Fig. 3C, Supplementary Fig. S8B). We measured the effect of Tempol on ISC proteins in different sub-cellular locations and with different functions: POLD (nuclear, DNA synthesis), PPAT (cytosolic, de novo purine synthesis), FECH (mitochondrial, heme biosynthesis), SDHB (mitochondrial, TCA cycle/electron transport chain), and LIAS (mitochondrial, lipoic acid synthesis). As cytosolic aconitase is potentially one of the most sensitive enzymes to ISC inhibition, we also measured cytosolic and mitochondrial aconitase activity. In all sensitive cell lines, Tempol induces a significant loss of aconitase activity and a decrease in ISC proteins with varying kinetics, with POLD exhibiting the most resistance (Fig. 3D). Conversely, Tempol did not affect aconitase activity or ISC protein levels in most resistant cell lines, with all but one line exhibiting no detectable change in the level or activity of any measured ISC protein (Fig. 3E). These data indicate that redox-dependent iron limitation differentially impacts ISC proteins.

Molecular oxygen (O_2_) can directly degrade ISCs, and culturing cells at a tissue level environmental O_2_ concentration (3% O_2_) can rescue many effects of ISC inhibition [9]. We confirmed this effect by inhibiting the essential ISC synthesis protein NFS1 in 10 cell lines cultured at 21% and 3% O_2_ (Supplementary Fig. S9). Since we hypothesize that redox-dependent iron limitation restricts cell growth by inhibiting ISC synthesis, we tested the O_2_ dependence of sensitivity to Tempol. Low O_2_ was sufficient to rescue the effects of Tempol on proliferation in sensitive cell lines (Fig. 3F). Tempol destabilizes ISC proteins at the same concentration that we observe this proliferation defect, and low O_2_ rescues most ISC protein levels (Supplementary Fig. S10). Low O_2_ did not revert IRP2 activation, suggesting that redox-dependent iron limitation still occurs in low O_2_. We then considered whether the effects of redox-dependent iron limitation on iron metabolism are also mitigated in resistant lines. Interestingly, Tempol treatment results in IRP2 upregulation in all cell lines, irrespective of sensitivity to redox-dependent iron limitation. Accordingly, we observe upregulation of TFR1 and downregulation of FTH1/FTL in all lines (Fig. 3D, E). IRP2 can be stabilized either via iron or ISC limitation. As we observe no ISC limitation in resistant cell lines (Fig. 3D, E), these data further demonstrate that Tempol increases IRP2 primarily via iron limitation. Taken together, we conclude that sensitive cell lines are less capable of maintaining ISC synthesis under redox-dependent iron limitation, even though the labile iron pool is restricted in all tested cell lines.

### Tempol treatment antagonizes ascorbate limiting redox-dependent iron utilization and activating HIF1α

**Fig. 4:**
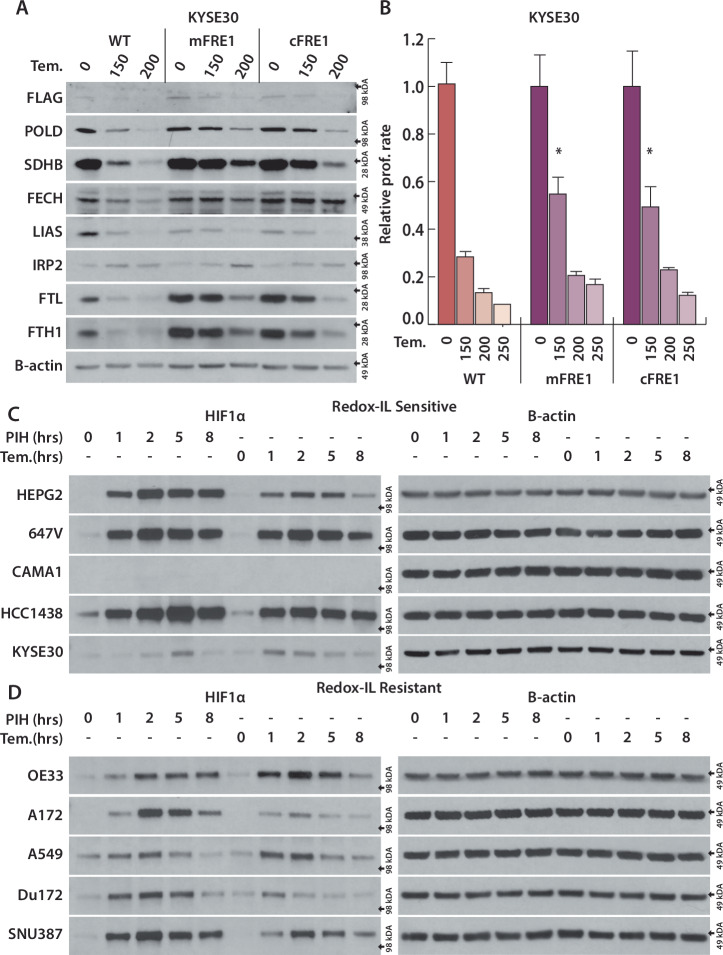
Tempol treatment antagonizes ascorbate limiting redox-dependent iron utilization and activating HIF1α. **A** Immunoblots for indicated proteins of lysates derived from KYSE30 cell line expressing mitochondrial(mFRE1) or cytocolic(cFRE1) located FRE1, treated with Tempol (Tem., 150, 200 μM) for 4 days. **B** Relative cell count for KYSE30 cell line expressing mitochondrial(mFRE1) or cytocolic(cFRE1) located FRE1, treated with Tem. (150, 200, 250 μM) for 4 days, Immunoblots for HIF1α and B-actin of lysates derived from 5 sensitive (**C**) and 5 resistant (**D**) cell lines, treated with PIH(50 μM) and Tem.(500 μM) for 1, 2, 5, 8 h. *p < 0.01, error bars are s.e.m.

Since iron redox state is important for its reactivity, binding and solubility, iron reductase/oxidizers are utilized to alter the iron redox state, including iron reductase FRE1 in Saccharomyces cerevisiae [51]. To test whether expression of an exogenous iron reductase can reverse our hypothesized effects of Tempol on redox-dependent iron limitation, we expressed mitochondrial and cytosolic targeted FRE1 in the Tempol-sensitive KYSE30 cell line. FRE1 expression induced a moderate rescue in proliferation and ISC-protein levels at same Tempol concentration(150 μM) in both mitochondrial and cytosolic targeted FRE1 expression (Fig. 4A, B). As we were unable to fully rescue the effects of Tempol treatment with FRE1 expression, these data indicate that iron limitation by Tempol primarily occurs indirectly, rather than via direct iron oxidation. Tempol can oxidize and deplete ascorbate in cell-free systems [49, 50]. Thus, we investigated whether Tempol can indirectly impact redox-dependent iron utilization by interfering with ascorbate.

Hypoxia-inducible factor 1-alpha (HIF1α) stability is regulated by prolyl hydroxylases (PHDs), which require αKG, ascorbate, and Fe^2+^ for their function, and ascorbate is hypothesized to maintain Fe^2+^ by reducing Fe^3+^, enabling efficient enzyme function [52, 53]. Therefore, we hypothesized that Tempol could facilitate redox-dependent iron limitation and stabilize HIF1α, either by directly oxidizing Fe^2+^ or inhibiting ascorbate function. We compared HIF1α levels after treatment with Tempol and PIH, a cell permeable iron chelator, in sensitive and resistant cell lines. Tempol increases HIF1α levels dramatically within 1 h similar to PIH treatment in all cell lines tested except CAMA1, which had no HIF1α increase in both Tempol and PIH treatments (Fig. 4C, D). Neither Tempol nor PIH increases IRP2 levels until 8 h and we do not observe significant changes in ISC protein levels on this time-scale (Supplementary Fig. S11A–C). Tempol has a strong antioxidant function, as such we compared antioxidant Trolox and reducing agent BME on their effect on iron metabolism and HIF1α levels. Neither Trolox nor BME alter HIF1α and IRP2 levels and has no effect on ISC protein stability (Supplementary Fig. S12). Tempol is derivate of another nitroxide, Tempo. Similar to Tempol, Tempo also increased HIF1α levels (Supplementary Fig. S13A, B). However, Tempo displayed minimal to no degradation of ISC proteins and no subsequent proliferation defect, indicating a more unique interaction of Tempol with iron metabolism (Supplementary Figs. S13C, S14). These data demonstrate that HIF1α activation precedes IRP2-mediated iron sensing mechanisms and ISC protein degradation upon both redox-dependent iron limitation by Tempol and iron chelation by PIH.

### Ascorbate completely rescues ISC degradation and cell growth defects induced by redox-dependent iron limitation

**Fig. 5:**
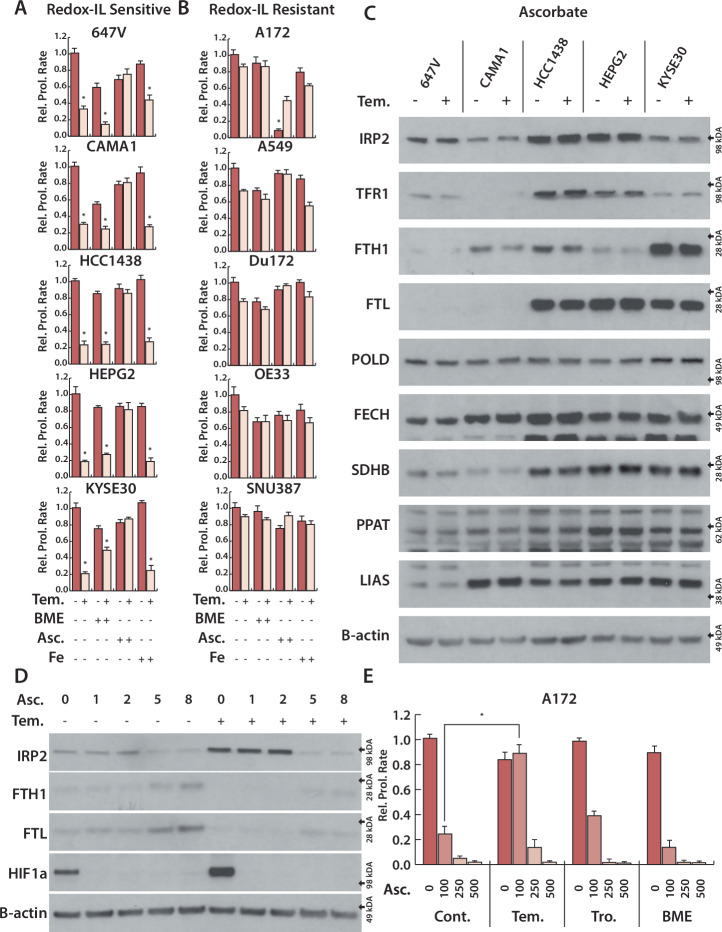
Ascorbate reverse redox-dependent iron limitation and rescues ISC homeostasis and cell growth. Relative cell count for 5 sensitive (**A**) and resistant (**B**) cell lines to redox-dependent iron limitation, treated with Tempol (Tem. 250), 2-mercaptoethanol (BME, 200 μM), Ascorbate (Asc., 250 μM), Iron (III) nitrate (Fe, 50 μM) for 4 days. **C** Immunoblots for indicated proteins of lysates derived from 5 sensitive cell lines, treated with Tem. (250 μM) and Asc (250 μM) for 4 days. **D** Immunoblots for indicated proteins of lysates derived from hPCC cells, treated with Tem. (250 μM) for 8 h and Asc (100 μM) for 1, 2, 5, 8 h. **E** Relative cell count for A172 cell line, treated with Asc (100, 250, 500 μM), Tem. (100 μM), BME. (100 μM) and Trolox(Tro, 100 μM) for 4 days *p < 0.01, error bars are s.e.m.

To further test our model, we asked whether the redox-dependent iron limitation could be counteracted by ascorbate in an expanded panel of cell lines, and whether a general reducing agent, 2-Mercaptoethanol (BME), could elicit a similar effect. Ascorbate rescues the effects of Tempol on cell growth completely in all cell lines tested, while iron supplementation and BME do not (Fig. 5A, B). Ascorbate also completely reverses IRP2 stabilization, changes to IRP2 target gene expression (TFR1, FTH, FTL), and loss of ISC protein levels in Tempol treated cells (Figs. 3D and 5C), while iron supplementation and BME treatment have a minimal effect on these phenotypes (Supplementary Fig. S15A, B).

We then asked whether redox-dependent iron limitation could counteract ascorbate in a model where ascorbate is limiting, and another model in which it is toxic. In most cell lines tested, we observe very low basal HIF1α levels, and ascorbate treatment does not alter IRP2 levels significantly. We detected high basal HIF1α levels in hPCC cells, which decreases significantly upon ascorbate treatment, suggesting that ascorbate is a limiting factor for HIF1α activation in these cells. Similar to HIF1α, IRP2 levels decreased rapidly upon ascorbate treatment. Tempol increases both IRP2 and HIF1α, which can be rapidly counteracted by ascorbate (Fig. 5D).

A172 cells are particularly sensitive to Ascorbate. As our data indicate Tempol primarily acts as an ascorbate antagonist, we asked whether Tempol would affect this sensitivity. While Tempol counteracts the effects of ascorbate, antioxidant Trolox and BME do not (Fig. 5E). Taken together, our data demonstrates that that anti-ascorbate effects of Tempol can facilitate redox-dependent iron limitation and ferroptosis sensitivity by this novel mechanism. Cells sensitive to redox-dependent iron limitation cannot maintain ISC synthesis upon Tempol treatment, inhibiting their cell growth.

## Discussion

**Fig. 6:**
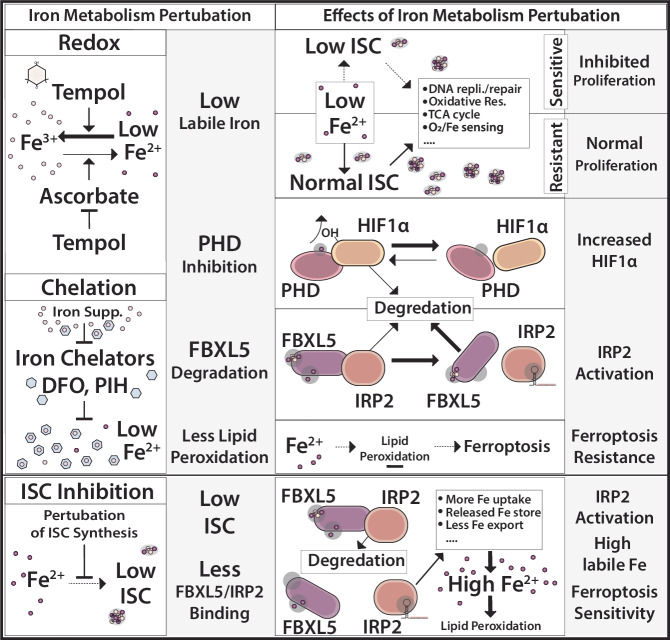
Perturbation of iron metabolism by redox-dependent iron limitation, iron chelation, ISC inhibition. Supplementary Table Legend. Correlation between TEMPOL Sensitivity and Gene Knockdown or Kncockout Effect (DepMap). Table reports the following: Depmap ID (Unique Cell Line ID from the DepMap Project); Tempol (Effect of TEMPOL on cell viability); Tempol Correl. (Correlation, pearson r, of Tempol Sensitivity from Column B with knockdown or knockout effect of the gene indicated in the subsequent columns); Gene: ACO2 Corr, (Correlation, pearson r, of Sensitivity to ACO1 suppression from Column D with knockdown or knockout of gene indicated in the column; ACO2 Hi: Low Diff (Difference between the average sensitivity between TEMPOL sensitive plus ACO2 resistant cell lines and TEMPOL sensitive plus ACO2 sensitive cell lines to knockdown or knockout of the gene indicated in the given column; *p*-val (*p*-value of a *t*-test for the difference reported in ACO2 Hi:Low Diff. Not corrected for multiple hypotheses).

Iron intake is essential for cell viability, and maintaining an optimal pool of bioavailable iron is challenging for highly proliferating cells [54]. Maintaining iron homeostasis is further complicated by the need for proper iron handling to avoid excessive iron-catalyzed oxidative damage, especially in the face of oxidative stress induced by various pathological states including oncogene activation in cancer [1]. Multiple iron handling mechanisms and anti-oxidant response pathways are designed to restrain oxidative damage caused by reactive molecules like iron [55–57]. Iron and aKG-dependent dioxygenases require the cofactor ascorbate (Vitamin C), thought to reduce ferrous iron and enable continued catalysis [53, 58]. Our data reveal that Tempol can facilitate redox-dependent iron limitation by altering the iron redox state, acting as an anti-ascorbate (Fig. 6). This feature of Tempol is similar to other anti-vitamins, such as methotrexate and warfarin [59]. Indeed, prior work indicates that ascorbate can reduce Tempol in cell-free assays [49], further suggesting that redox-dependent iron limitation by Tempol may be facilitated by an anti-ascorbate effect in addition to direct oxidation of Fe^2+^ to Fe^3+^. In contrast, BME, commonly used as a reducing agent in cultured cells, minimally rescues the effects of redox-dependent iron limitation, underscoring the specific importance of ascorbate on iron redox cycling. Based on the interesting properties of redox-dependent iron limitation, future work may be directed at generating related anti-ascorbate molecules similar to Tempol with more potent effects and better pharmacokinetics and pharmacodynamics.

Similar to iron chelation, redox-dependent iron limitation suppresses ferroptosis, a form of cell death mediated by Fe^2+^-catalyzed peroxidation of membrane lipid polyunsaturated fatty acid side chains [4]. Our finding emphasize the importance of iron redox state not only in cellular iron metabolism, but also in ferroptosis. Other ferroptosis suppressors directly chelate iron, are lipophilic radical-trapping anti-oxidants, or limit the pool of membrane lipids with polyunsaturated fatty acid side chains [60]. While we cannot exclude a dual anti-ferroptotic role of Tempol as an anti-oxidant, redox-dependent iron limitation and suppression of ferroptosis occur at same concentrations similar to iron chelators, indicating that Tempol primarily inhibit ferroptosis by a novel Fe^2+^-limiting mechanism.

We observe that cell lines differ in their ability to maintain ISC synthesis upon redox-dependent iron restriction, despite similar effects on the iron sensor IRP2. Indeed, the effect on cell proliferation induced by iron chelators like DFO only correlates weakly with the effects of redox-dependent iron limitation, further underscoring this distinction. Redox-dependent iron limitation upregulates IRP2 and HIF1α in all cell lines, however, only a subset of cell lines cannot maintain ISC synthesis, leading to proliferative defects upon redox-dependent iron limitation. ACO2 gene essentiality significantly correlates with the sensitivity to redox-dependent iron limitation, which could partially explain inhibition of cell growth upon ISC loss. We previously showed that cell lines differ in their sensitivity to ISC inhibition, with triple negative breast cancer cell lines exhibiting a heightened dependency on ISC synthesis due to a context-specific requirement for the ISC protein POLE [61]. Thus, ACO2 can represent another important determinant of sensitivity to ISC inhibition, especially in the context of redox-dependent iron limitation.

Environmental O_2_ concentration is an important factor influencing ISC metabolism, as O_2_ can degrade ISCs by direct oxidation [9]. Tissue level O_2_ concentrations limit the effects of redox-dependent iron limitation on cell-essential ISC proteins, while still exerting effects on the LIP and iron-dependent enzymes. As most cells in human body are exposed to O_2_ concentrations well-below those standardly used in cell culture [62], we anticipate that redox-dependent iron limitation would be effective in modulating iron metabolism in the body with only limited effects on ISC protein function. On the other hand, redox-dependent iron limitation may target ISC proteins in tissue exposed to higher O_2_ concentration more effectively, explaining how Tempol can target ISC proteins required for viral replication in SARS-CoV-2 in lung airways.

## Methods

### Reagents

Reagents were obtained from the following sources. Antibodies: β-actin (8457), FTH1 (3998), IRP1 (20272), IRP2 (37135), and TFR1 (CD71, 13208) were from Cell Signaling Technologies; NFS1 (sc-365308), ISCU (sc-271468), and FECH (sc-377377) were from Santa Cruz Biotechnology; and FTL (ab218400), SDHB (ab175225), POLD1 (ab186407) were from Abcam. PPAT (15401-1-AP), LIAS (11577-1-AP) were from Proteintech. Cells: 5637, A-172, 786-O, A-253, A-375, A549, ACHN, AGS, AN3 CA, AsPC-1, BT-20, BT-549, BxPC-3, CAL 27, Calu-1, CAMA-1, DBTRG-05MG, Detroit 562, DoTc2 4510, DU 145, FaDu, HCC70, HCC827, HCT-15, HEC-1-A, HEC-1-B, Hep G2, Hs 578T, HT-1080, J82, LN-229, LNCaP, MDA-MB-231, MIA PaCa-2, NCI-H2052, NCI-H647, NCI-N87, PC-3, SH-SY5Y, SK-ES-1, SK-MEL-2, SK-OV-3, SNU-387, SNU-423, TT, U-2 OS, U-87 MG, UM-UC-3 from American Type Culture Collection; 647-V, BEN, CAL-33, EFM19, KYSE-30, OE-33, SK-MEL-30 from DSMZ, KP-2, OUMS-23 from JCRB, Chemicals: RPMI (MT10040CV) was from Corning; PrimeStar HS DNA Polymerase Premix (R040A) was from Takara; RNeasy Plus Mini Kit (74136), Qiaprep Spin Miniprep Kit (27106), and QIAquick Gel Extraction Kit (28706) were from Qiagen. 4-hydroxy-2,2,6,6-tetramethyl-1-piperidinyloxy (Tempol, 27051), Erastin (S7242) was from Selleckchem. (+)-Sodium L-ascorbate(A4034), 6-Hydroxy-2,5,7,8-tetramethylchromane-2-carboxylic acid (Trolox, 238813), Pyridoxal isonicotinoyl hydrazine (PIH, 528110), deferoxamine mesylate (D9533), ferrostatin-1 (SML0583), iron(III) nitrate (F8508), Hydrogen Peroxide(216763) were from Sigma-Aldrich; KI696 (HY-101140) from MedChemExpress shRNAs were obtained from the The RNAi Consortium (TRC) or identical sequences cloned into pLKO.1p: shGFP, TRCN0000072186; shNFS1, TRCN0000229753.

### Cell culture

Cell lines were tested for mycoplasma by polymerase chain reaction (PCR), and the authenticity of cell lines was verified by short tandem repeat (STR) profiling. Cells were cultured in RPMI (11875093, ThermoFisher) supplemented with 10% fetal bovine serum (Peak Serum) and 1% penicillin and streptomycin (15140122, ThermoFisher), adherent cells are passaged using 0.05% Trypsin (15090046, ThermoFisher), diluted in PBS. Cells are incubated in an incubator maintaining 3% O_2_, when stated. Single-cell clones are obtained by infecting parental cells with Cas9 system (pLentiCRISPR V2, Addgene), specific sgRNA for knockout, and cDNA vectors for exogenous expression for indicated genes. After selection, cells are plated in 96 wells in serial dilution and single clones are screened with Western blot. Target sequences for sgRNAs are used in the study IRP1 clones, (i) TGGCTCAGGAATCATCCACC, (ii) CCGCCAGGTTGGGGTAGTGG; FBXL5, GGTGGGGCTCTACTGCGACA.

### Cell proliferation and viability assays

Cell viability assay: 2000–3000 cells per well are plated to white, clear bottom 96-well (655098, Greiner) in triplicates after indicated infection or treatment, and further treatments are done as indicated. Cell viability is measured using the CellTiter-Glo Luminescent Cell Viability Assay (Promega G7573) in a plate reader, and empty wells used as baseline to correct background. Cell proliferation: 25,000 cells per well are plated in 12 wells (FB012928, Fisher) in triplicate. After one day, cells are treated as indicated for 4 days. Cells are counted using Beckman Z2 Coulter Counter. Each assay is replicated three times.

### Immunoblotting

50,000–100,000 cells per well are plated in 6 wells (FB012927, Fisher) for 3–4 day treatment, 250,000 cells were plated for 0–1 day treatments. One day after plating, cells are treated as indicated. After treatment, cells are washed with cold phosphate-buffered saline (PBS) and lysed by addition of lysis buffer [50 mM tris (pH 7.4), 150 mM NaCl, 1% NP-40, 0.1% sodium deoxycholate, 0.1% SDS, and 2 mM EDTA] with a protease inhibitor cocktail (Sigma-Aldrich, 5892791001). Cell lysates are collected after 10-min incubation on ice and centrifuged at 4 °C at 21,000 × *g* for 10 min. Supernatant is collected, and protein concentration is quantified using a bicinchoninic acid (BCA) protein assay kit (Pierce 23225). Eight micrograms protein is loaded with loading buffer into Bolt 4 to 12% bis-tris polyacrylamide gels (Thermo Fisher Scientific, NW04125BOX) with protein ladder (Thermo Fisher Scientific, LC5925), electrophoresed in running buffer (Thermo Fisher Scientific, B0002) at 110 V for 1 hours, and transferred in transfer buffer [N-cyclohexyl-3-aminopropanesulfonic acid (2.2 g liter^−1^), NaOH (0.45 g liter^−1^), and 10% ethanol] to a polyvinylidene difluoride membrane (Millipore, IPVH00010) at 75 V for 3.5 h. Membranes are blocked with 5% bovine serum albumin (BSA), incubated with indicated antibody for 1–2 h at room temperature, washed three times with TBS-T (tris buffered saline, 0.1% Tween-20), incubated with secondary antibody for 1 h at room temperature, washed three times with TBS-T, and developed using ECL substrate (Thermo Fisher Scientific, PI-32106) with autoradiography film (WorldWide, 41101001) in dark room. Each experiment is replicated three times, representing figures are shown in the results.

### qPCR

50,000–100,000 cells per well are plated in 6 wells (FB012927, Fisher), and after one day, cells are treated as indicated. After treatment, RNA was isolated by column purification (RNeasy Kit, Qiagen). One microgram of total RNA is reverse transcribed to cDNA using reverse transcriptase (Thermo Fisher Scientific, 18090050) with 1 μl of RNase OUT (10777019, Invitrogen). Quantitative PCR (qPCR) was performed on cDNA using SYBR green quantification (Thermo Fisher Scientific, K0223). Expressions are quantified relative to ACTB and RPL13A. Each assay is replicated three times. Primers used are as follows:

Β-Actin (ACTB), AAGGGACTTCCTGTAACAATGCA (forward), and CTGGAACGGTGAAGGTGACA (reverse); FTH1, AGGTGCGCCAGAACTACCA (forward) and TCGCGGTCAAAGTAGTAAGACATGG (reverse); RPL13A, CATAGGAAGCTGGGAGCAAG (forward) and GCCCTCCAATCAGTCTTCTG (reverse); TFR1, AGGCCAATGTCACAAAACCAA (forward) and AGCCAATCATAAATCCAATCAAGAA (reverse).

### In gel aconitase assay

Protocol was adapted from ref. [18]. 50,000–100,000 cells per well are plated to 6 wells (FB012927, Fisher) for 3–4 day treatment, 250000 cells were plated for 0–1 day treatments. One day after plating, cells are treated as indicated. After treatment, cells are washed with cold PBS and lysed by addition of lysis buffer [40 mM KCl, 25 mM tris-Cl (pH 7.5), 1% Triton X-100, 0.1 M fresh DTT, 1 M Na Citrate, and 1 M MnCl_2_] with a protease inhibitor cocktail (Sigma-Aldrich, 5892791001). After 10-min incubation on ice, lysates are collected and centrifuged at 4 °C at 21,000 × *g* for 10 min. Supernatant is collected, and protein concentration is quantified using a Bradford protein assay kit (BioRad, 5000006). 16 micrograms of protein were loaded into 8% polyacrylamide/TBE gel supplemented with 1 M Na citrate and run at 185 V for 3 h in running buffer [25 mM tris base, 192 mM glycine, and 3.6 mM citrate (pH 8)]. Gel was washed with distilled water and incubated at 37 °C for 5–45 min in reaction solution [100 mM tris (pH 8.0), 1 mM nicotinamide adenine dinucleotide phosphate (Sigma-Aldrich, 10128031001), 2.5 mM cis-aconitate (Sigma-Aldrich, A3412), 5 mM MgCl2, 1.2 mM MTT (3-(4,5-dimethylthiazol-2-yl)-2,5-diphenyltetrazolium bromide) (Thermo Fisher Scientific, M6494), 0.3 mM phenazine methosulfate (Sigma-Aldrich, P9625), and isocitrate dehydrogenase (5 U/ml; Sigma-Aldrich, I2002)]. Gel is washed three times with distilled water for 5 min to remove background and gel is imaged. Each experiment is replicated three times, representing figures are shown in the results.

### Ferrozine assay for ferrous iron

4 μL of indicated concentrations of Tempol (27051, Selleckchem), Trolox (238813, Sigma) and DFO (D9533, Sigma) added to 196 μM freshly prepared 50 μM Ammonium iron (II) sulfate hexahydrate (215406, Sigma) and mixed. The mixture was incubated at room temperature for 5 min. 5 μL of a 20 mM stock of Ferrozine (ThemoScientific, 1266615) was added and mixed. Absorbance was measured at 562 nm. Each assay is replicated three times.

### Statistical analysis

Cell viability, proliferation assays, and mRNA quantification are performed in at least biological triplicate, the entire experiment performed at least three times, and the results of these three experiments combined and reported as *n* = 3. Immunoblots are repeated from at least three independently generated cell lysates, and representative immunoblots are shown. Student’s *t* distribution is assumed, which is not contradicted by data, and *P* values in the figures are the result of heteroscedastic Student’s *t* tests. No samples were excluded from analysis, no sample size estimates or randomization were used, experiments were not conducted blinded. *P* values less than 0.01 were considered significant.

## Supplementary information

Supplementary Figures and Legends

Uncropped blot and gel images

Supplementary Table

## Data Availability

All data generated or analyzed during this study are included in this published article and its supplementary information files.
