# Supplementary material for: Redox cycling nitroxide limits cellular iron availability and selectively inhibits iron-sulfur cluster metabolism
Source: Cell Death Discov. 2026 Mar 24;12:165. doi: 10.1038/s41420-026-03042-w (PMC13039289; doi:10.1038/s41420-026-03042-w)
Supplement: Supplementary file 1 — Supplementary Figures and Legends [file 41420_2026_3042_MOESM1_ESM.docx]

**SUPPLEMENTARY FIGURES AND LEGENDS**


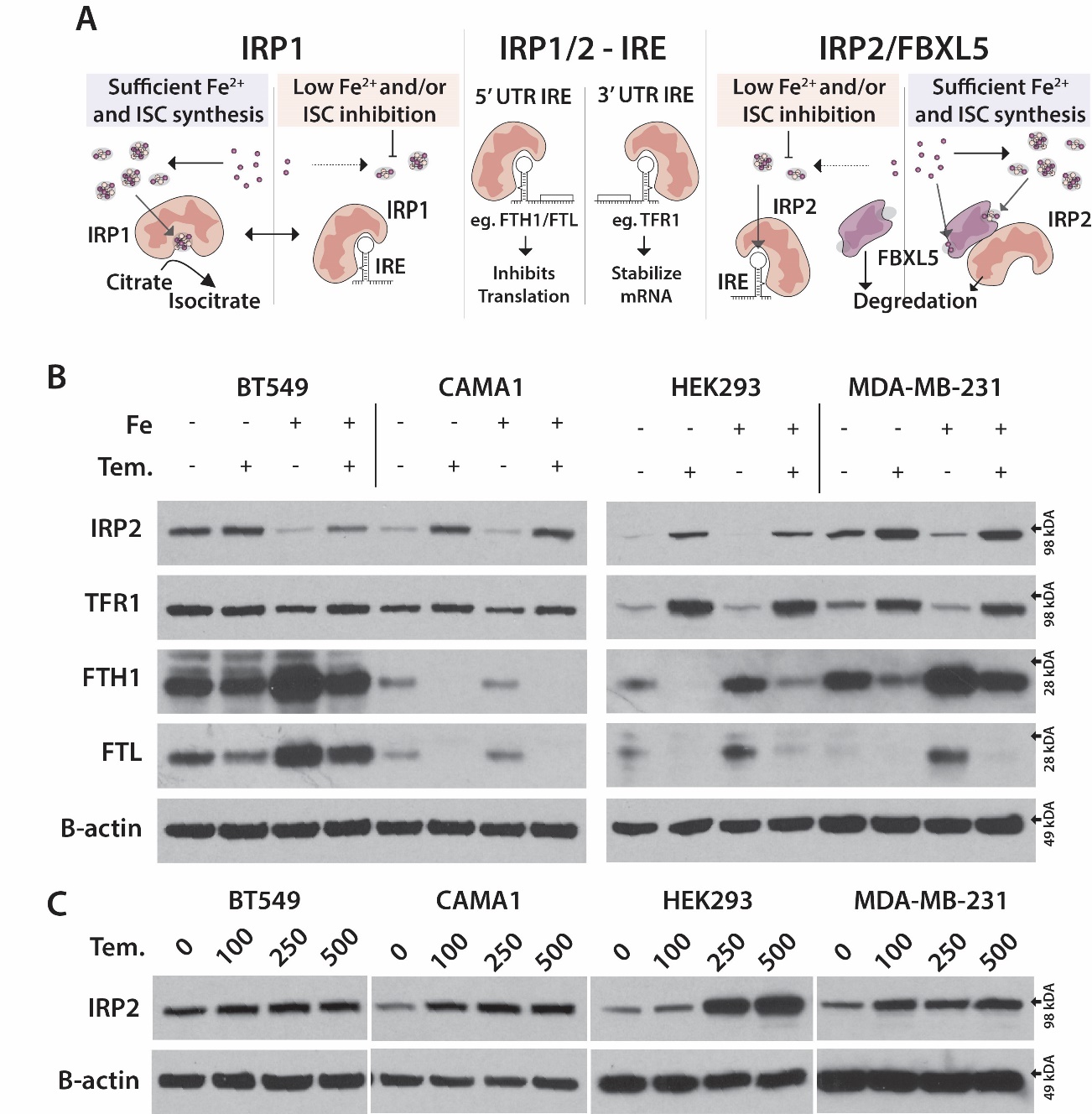


**Supp. Figure 1 | Tempol activates IRP2 in cell-line dependent manner A**, Schematic of IRP1 and IRP2 pathways. Left, IRP1 binds an ISC, preventing its association with IRE step-loops in target mRNAs. Right, IRP2 is degraded by FBXL5 when iron is high. Middle, when iron or ISC levels are low, IRP1 and IRP2 can bind IREs, regulating target mRNA stability or translation. **B**, Immunoblots from lysates derived from indicated cell lines, treated with Iron (III) nitrate (Fe, 24uM), Tempol (Tem, 500uM) for 3 days for indicated proteins. **C**, Immunoblots from lysates derived from indicated cell lines, treated with Tempol (100, 125, 500uM, 3 days) for indicated proteins.


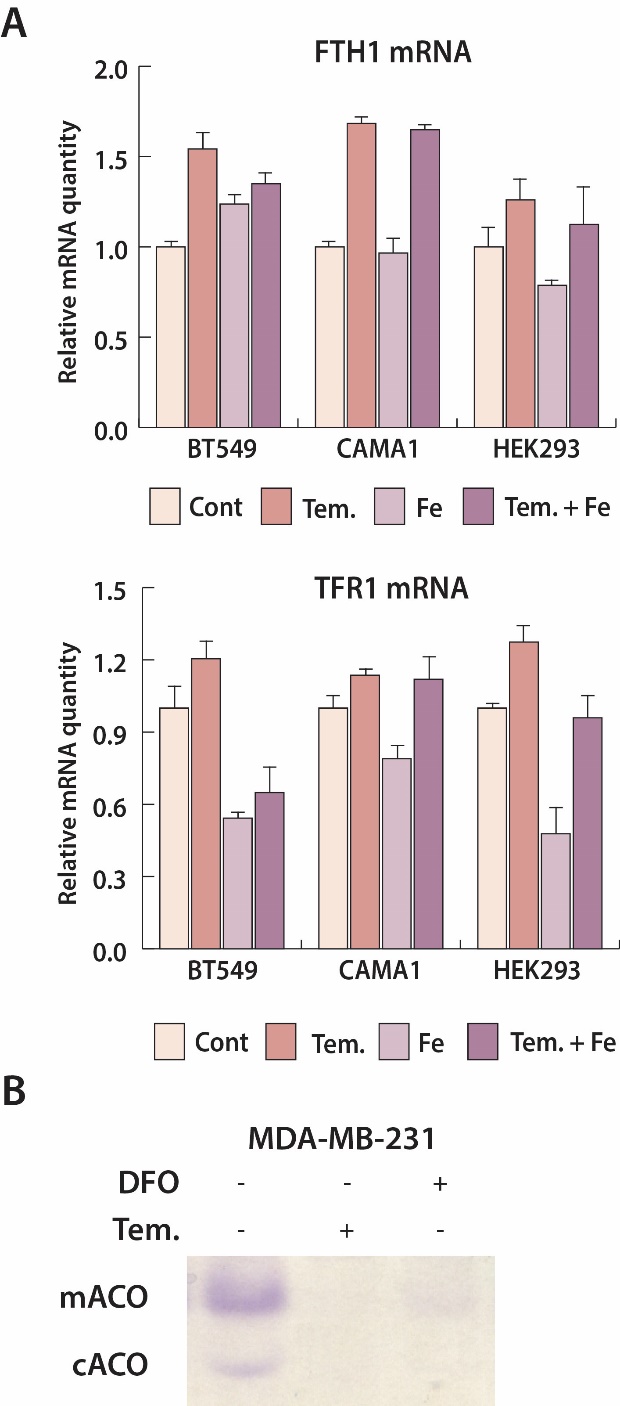


**Supp. Figure 2 | Tempol regulates IRP2 target TFR1 mRNAs and inhibits aconitase activity A,** Relative abundance of FTH1 and TFR1 mRNA in BT549, CAMA1, HEK293 cell lines, treated with Iron (III) nitrate (Fe, 24uM), Tempol (Tem., 500uM) for 3 days. **B,** Aconitase assay for mitochondrial (mACO) and cytosolic (cACO) aconitases of MDA-MB-231 cell line, treated with Deferoxamine (DFO, 50uM) and Tem. (500uM) for 1 day.


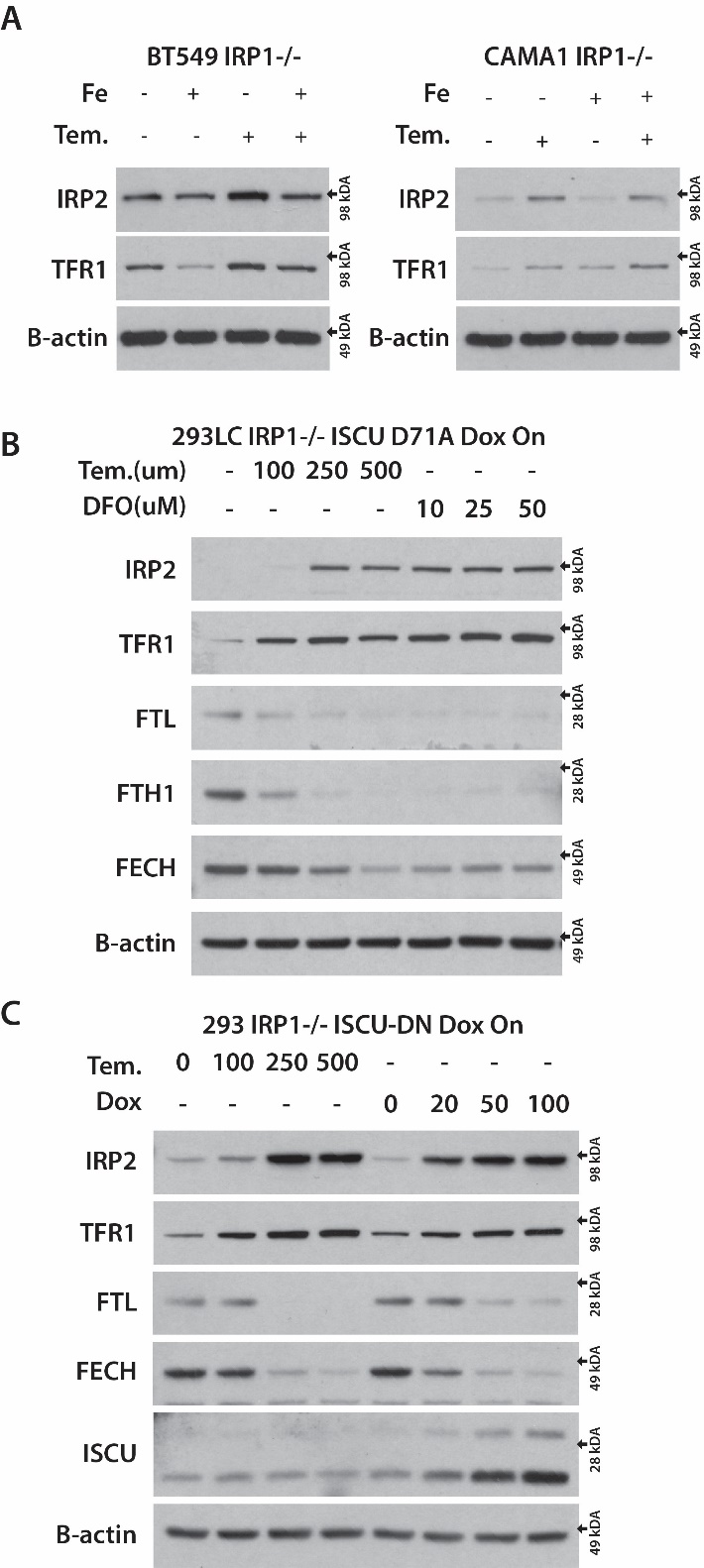


**Supp. Figure 3 | Tempol activates IRP2 independent of IRP1 similar to iron chelation and Fe-S inhibition. A,** Immunoblots for indicated proteins of lysates derived from IRP1 knockout BT549 and CAMA1 cell lines, treated with Iron(III) nitrate (24uM, Fe), Tempol (Tem., 500uM) for 3 days. **B**, Immunoblots for indicated proteins of lysates derived from IRP1 knockout 293 HEK cell line expressing doxycycline (Dox)-inducible ISCU-DN, treated with Tem. (100, 250, 500uM) or Deferoxamine (DFO, 10, 25, 50uM) for 3 days. **C**, Immunoblots for indicated proteins of lysates derived from IRP1 knockout 293 HEK cell line expressing doxycycline (Dox)-inducible ISCU-DN, treated with Tempol (Tem., 100, 250, 500uM) or Dox(20, 50, 100nM) for 3 days.


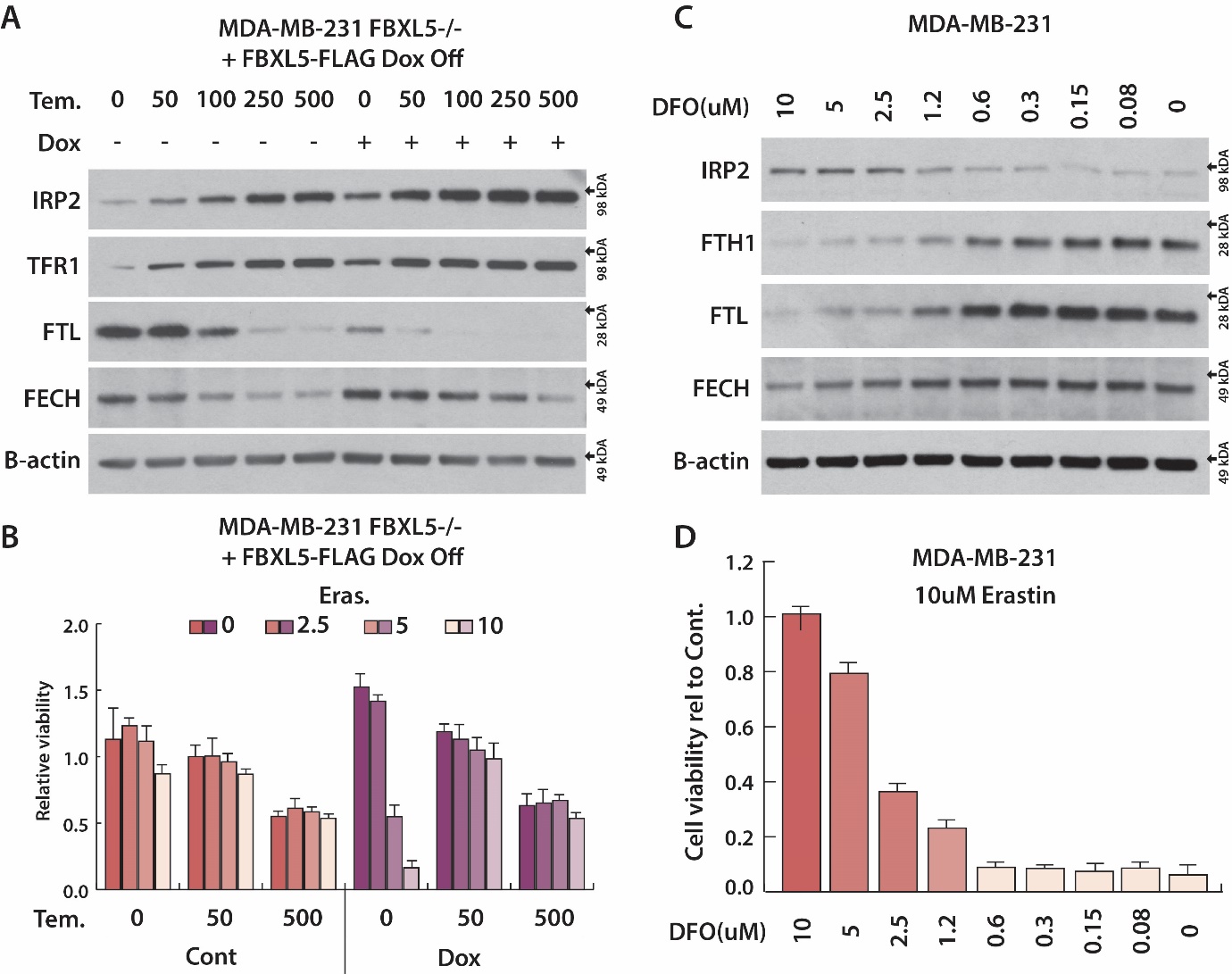


**Supp. Figure 4 | Tempol activates IRP2 and inhibits ferroptosis similar to iron chelator DFO A,** Immunoblots for indicated proteins of lysates derived from FBXL5 knock-out MDA-MB-231 cell line expressing doxycycline (Dox)-repressible FBXL5, treated with Tempol (Tem., 50, 100, 250, 500uM) and Dox(100nM) for 3 days. **B**, Relative viability of FBXL5 knock-out MDA-MB-231 cell line expressing Dox-repressible FBXL5, treated with Tem(50, 500uM), Dox(100nM) and Erastin (Eras. 2.5, 5, 10uM) for 3 days. **C**, Immunoblots from lysates derived from MDA-MB-231, treated with DFO as indicated concentration for 1 day for indicated proteins **D**, Relative viability of MDA-MB-231, treated with DFO as indicated concentration for 1day.


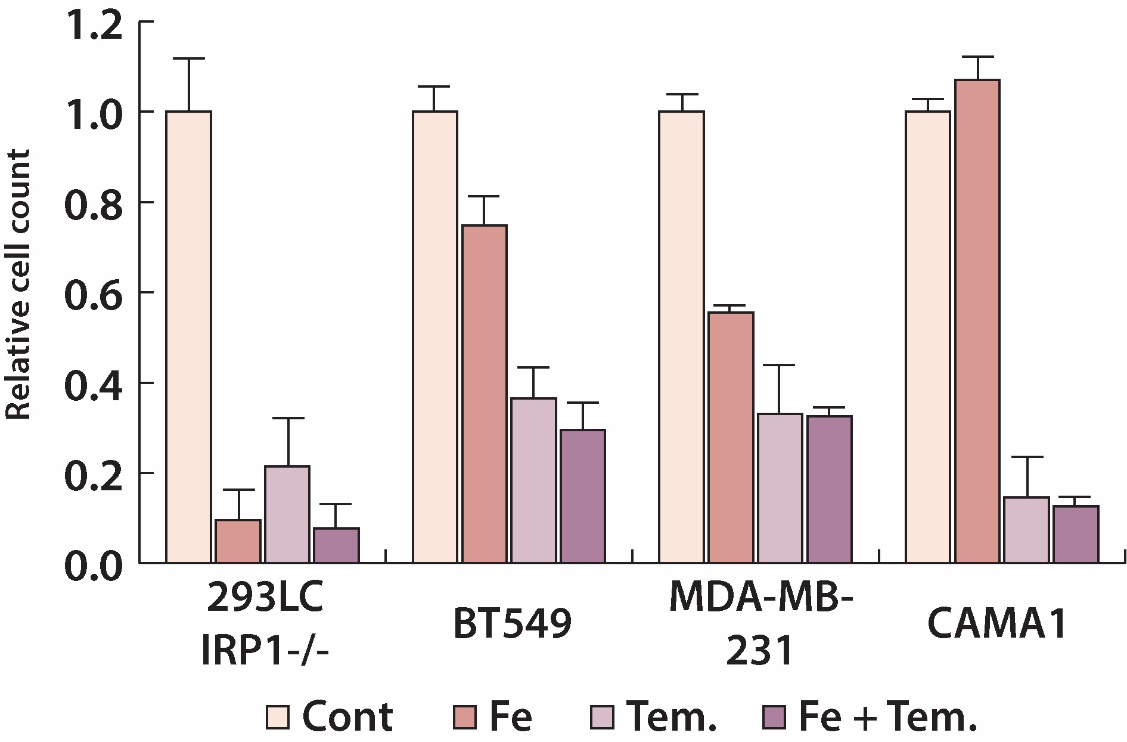


**Supp. Figure 5 | Iron supplementation does not rescue Tempol dependent growth inhibition** **A**, Relative cell counts of BT549, CAMA1, MDA-MB-231 and IRP1 knockout HEK293, treated with Iron (III) nitrate (240uM, Fe), Tempol (500uM, Tem.) for 4 days


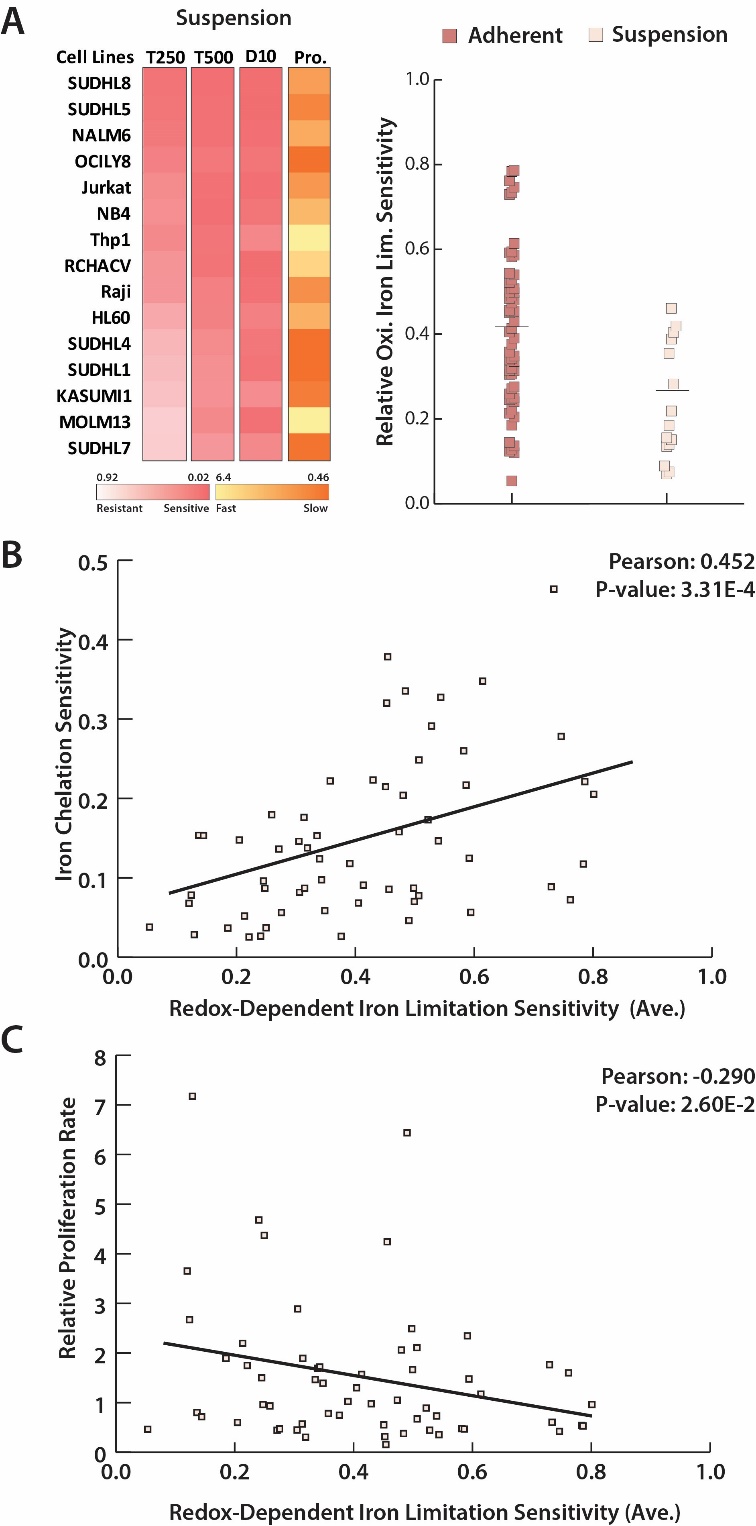


**Supp. Figure 6 | Redox-dependent iron limitation by Tempol moderately correlates with iron chelation by DFO A,** Left **-** Heatmap for relative proliferation rate (Pro.) and the relative cell count of 15 suspension cell lines, treated with Deferoxamine (DFO, 10uM), Tempol (Tem. 250, 500uM) for 4 days. Right – Relative sensitivity to redox-dependent iron limitation, estimated by relative cell count of 60 adherent cell lines and 15 suspension cell lines, treated with Tem. (250, 500uM) for 4 days. **B**, Correlation between relative sensitivity to iron chelation(DFO) and redox-dependent iron limitation(Tem.), estimated by relative cell count of 60 adherent cell lines, treated with DFO (10uM), Tem. (250, 500uM) for 4 days, respectively. **C**, Correlation between proliferation rate and relative sensitivity to redox-dependent iron limitation, estimated by relative cell count of 60 adherent cell lines, treated with Tem. (250, 500uM) for 4 days.


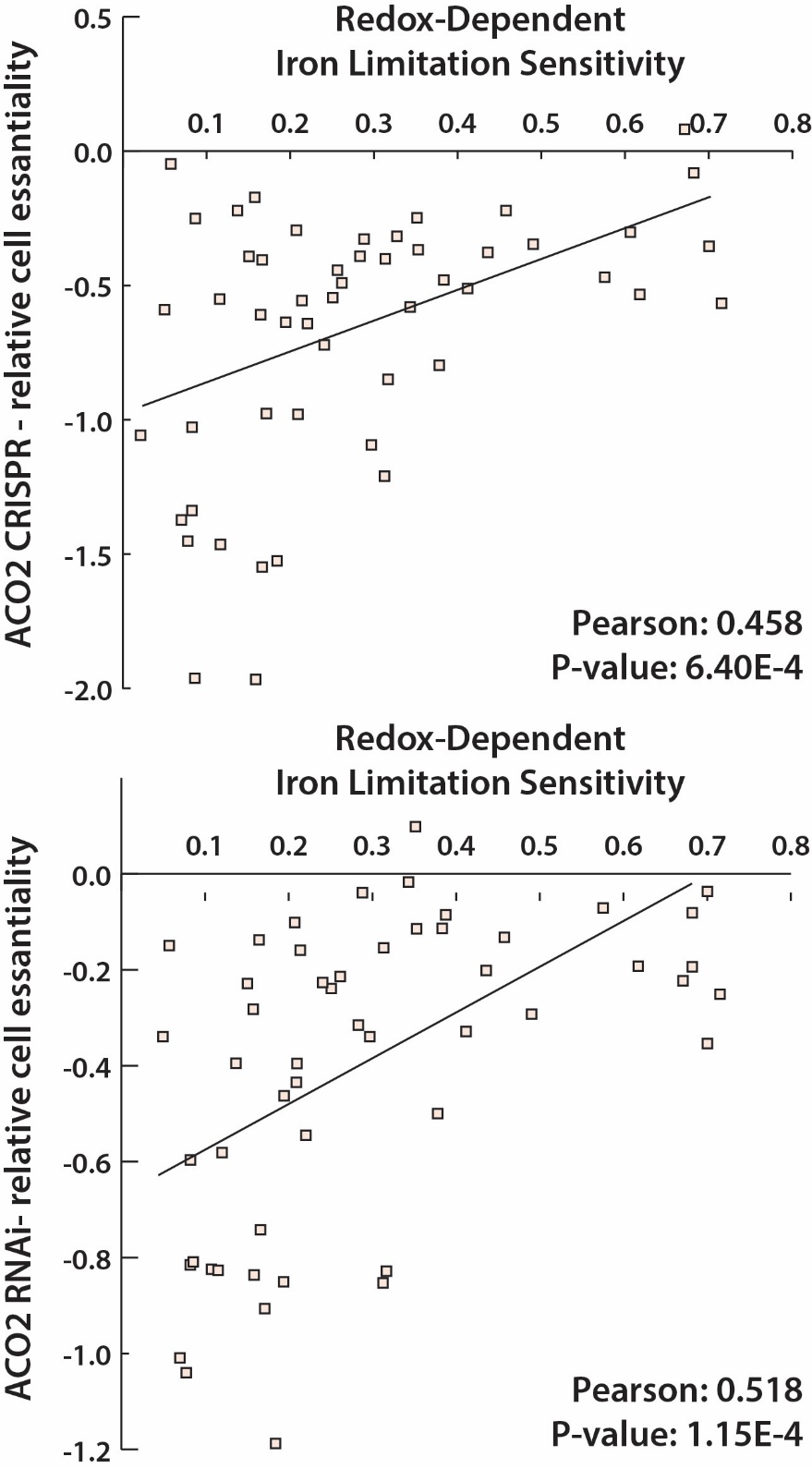


**Supp. Figure 7 | Redox-dependent iron limitation by Tempol moderately correlates with sensitivity to ACO2 inhibition.** Correlation between ACO2 relative gene essentiality and sensitivity to redox-dependent iron limitation; ACO2 gene essentiality date is obtained from DepMap databases for CRISPR (above) and RNAi (below), redox-dependent iron limitation sensitivity is estimated by relative cell count of 60 adherent cell lines, Tempol (250, 500uM) for 4 days.


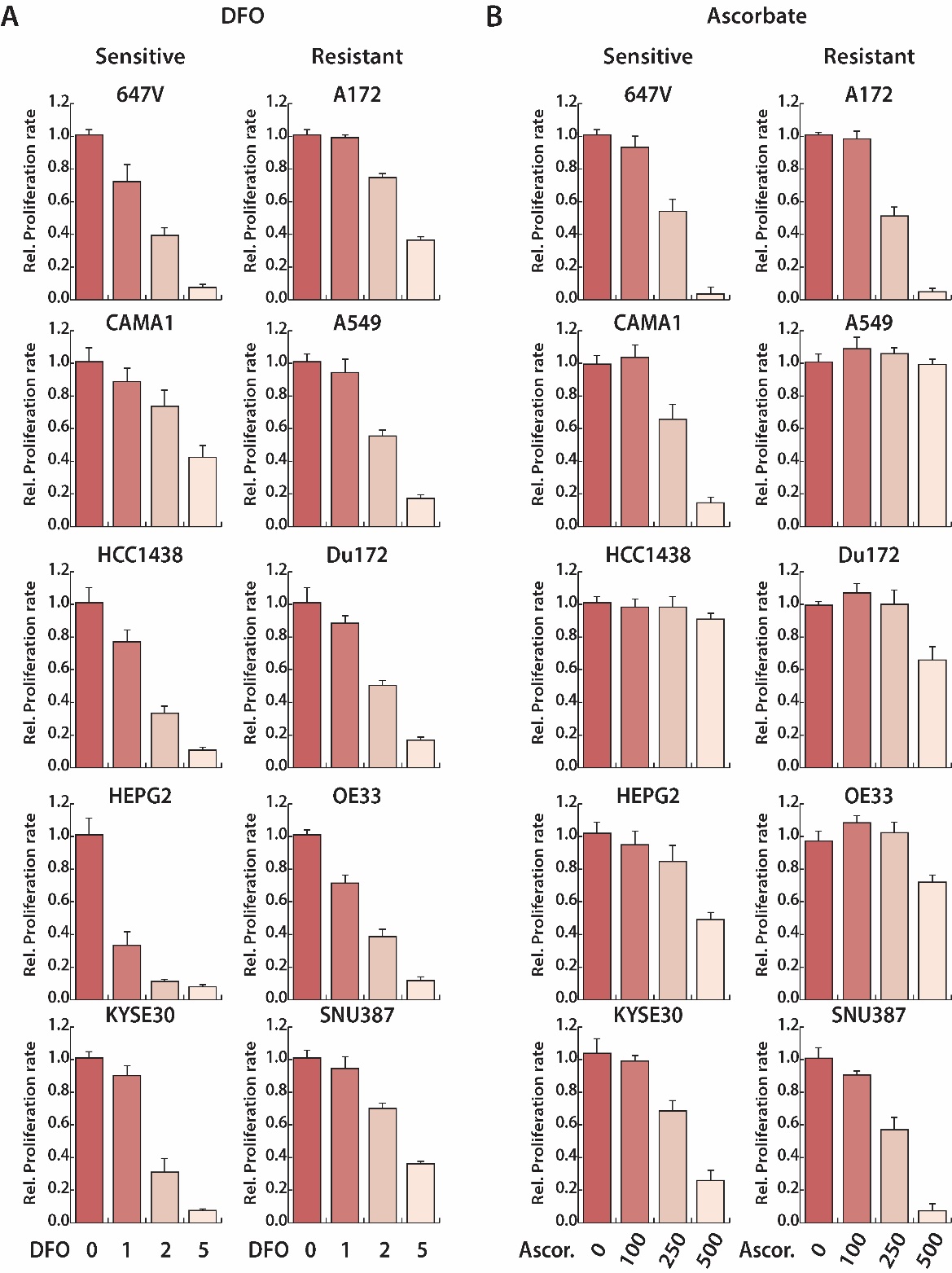


**Supp. Figure 8 | Sensitivity to DFO and Ascorbate do not significantly correlate with sensitivity to Tempol A,** Relative cell count for 5 sensitive and 5 resistant cell lines, treated with Deferoxamine (DFO, 1, 2, 5uM) for 4 days. **B**, Relative cell count for 5 sensitive and 5 resistant cell lines, treated with Ascorbate (Asc., 100 250, 500uM) for 4 days.


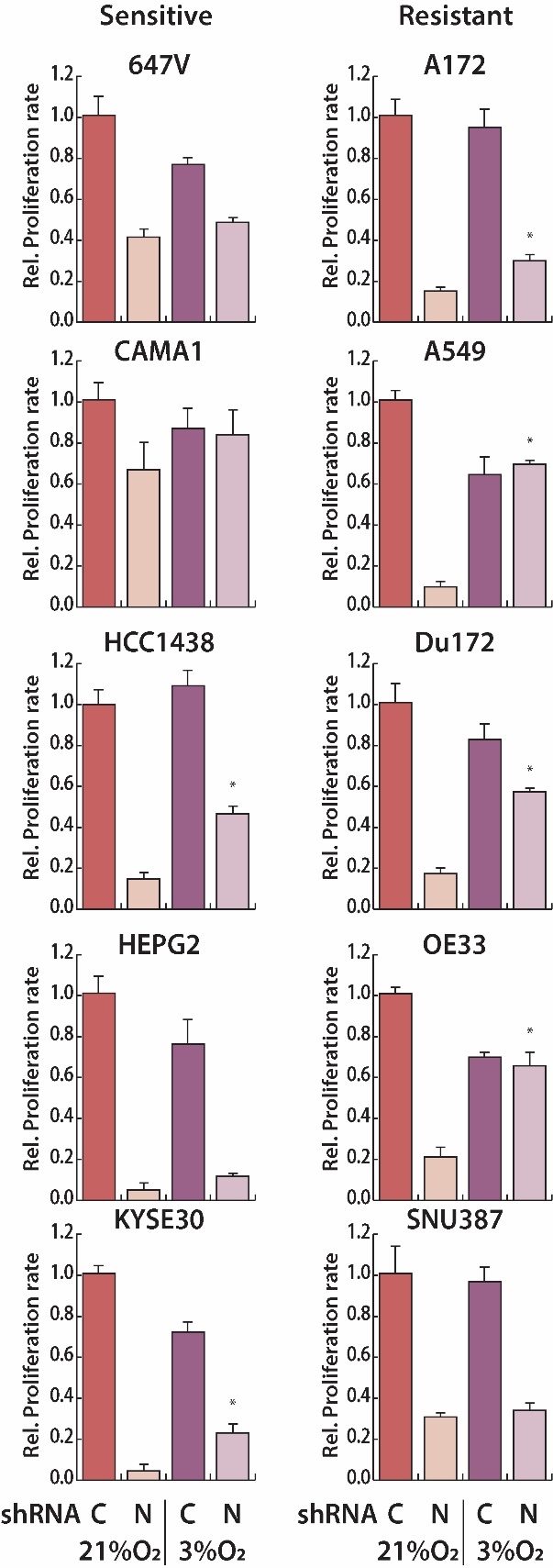


**Supp. Figure 9 | Fe-S inhibition by NFS1 suppression is rescued by lower oxygen concentration.** Relative cell count for 5 sensitive and 5 resistant cell lines, expressing a non-targeting shRNA (C) or shRNA targeting NFS1 (N), incubated at 21% and 3% O_2_ for 4 days. * p < 0.01, error bars are s.e.m..


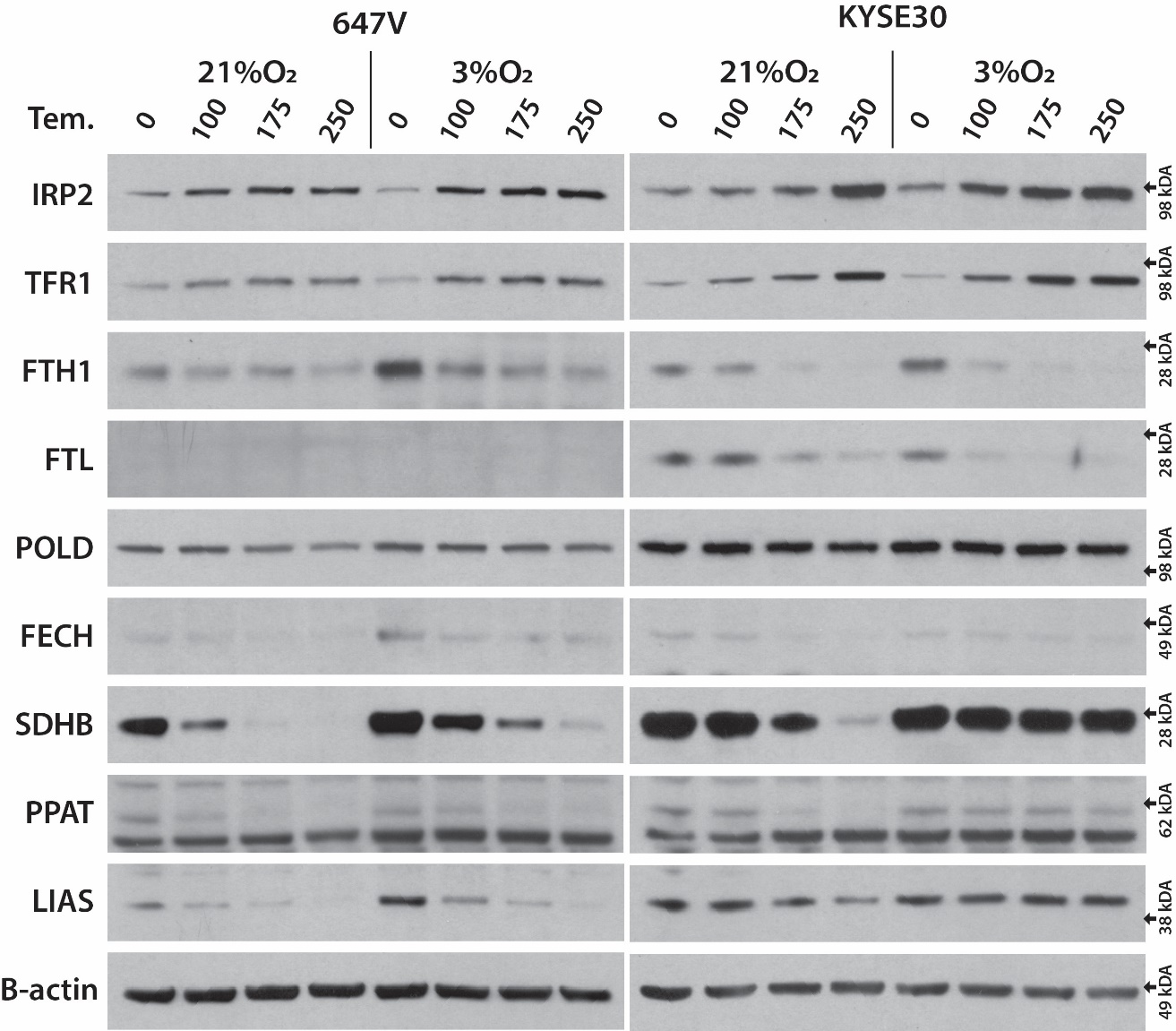


**Supp. Figure 10 | Degradation of FE-S proteins by Tempol is moderately rescued by low oxygen concentration.** Immunoblots for indicated proteins of lysates derived from 647V and KYSE30, treated with Tempol (Tem. ,100, 175, 250uM), incubated at 21% and 3% O_2_ for 4 days.


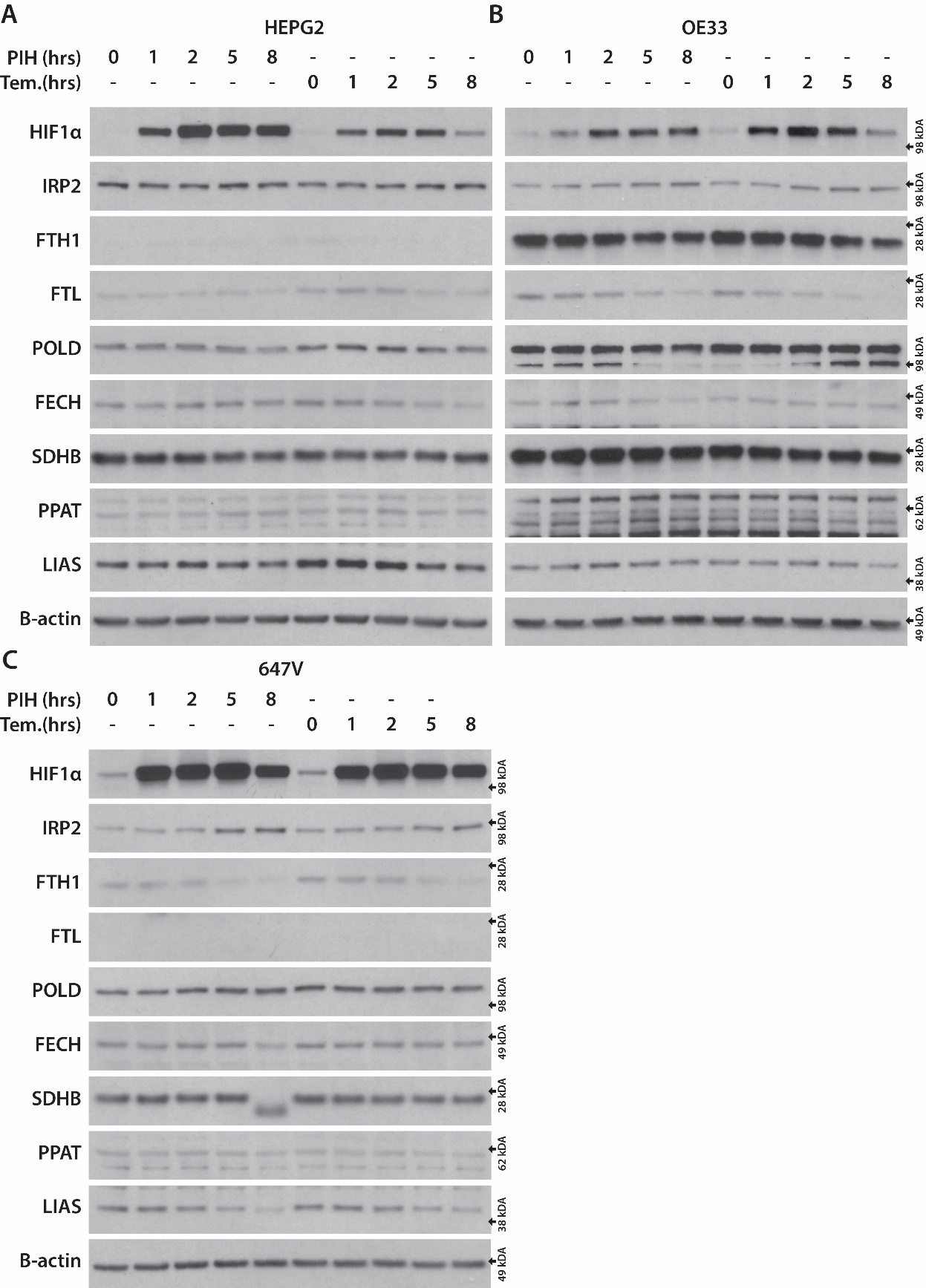


**Supp. Figure 11 | Short treatment with Tempol and iron chelators activates HIF1alphe before activation of IRP2 and Fe-S protein degradation. A,B,C** Immunoblots for indicated proteins of lysates derived from HEPG2(A), OE33(b) and 647V(C) cell lines, treated with pyridoxal isonicotinoyl hydrazine (PIH, 50uM) and Tempol (Tem., 500uM) for 1, 2, 5, 8 hrs.

**
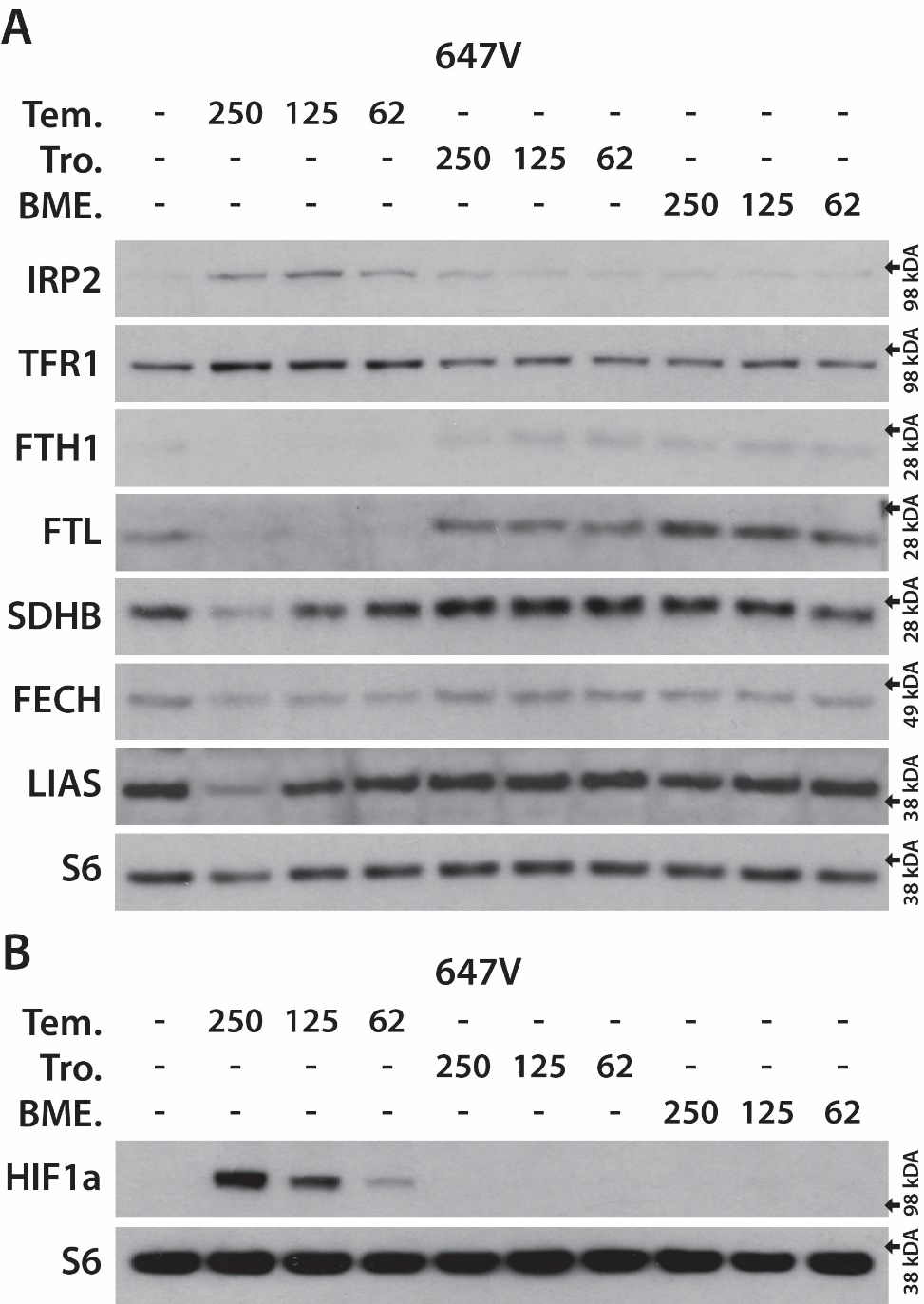
**

**Supp. Figure 12 | Tempol, but not antioxidant Trolox or reducing agent BME, alters HIF1a and IRP2 levels and decreases ISC protein stability. A, B.** Immunoblots for indicated proteins of lysates derived from 647V cell line, treated with Tempol (Tem., 250, 125, 62uM), Trolox(Tro. 250, 125, 62uM), beta mercaptoethanol (BME 250, 125, 62uM) for 4 days(A) or 2hrs (B).

**
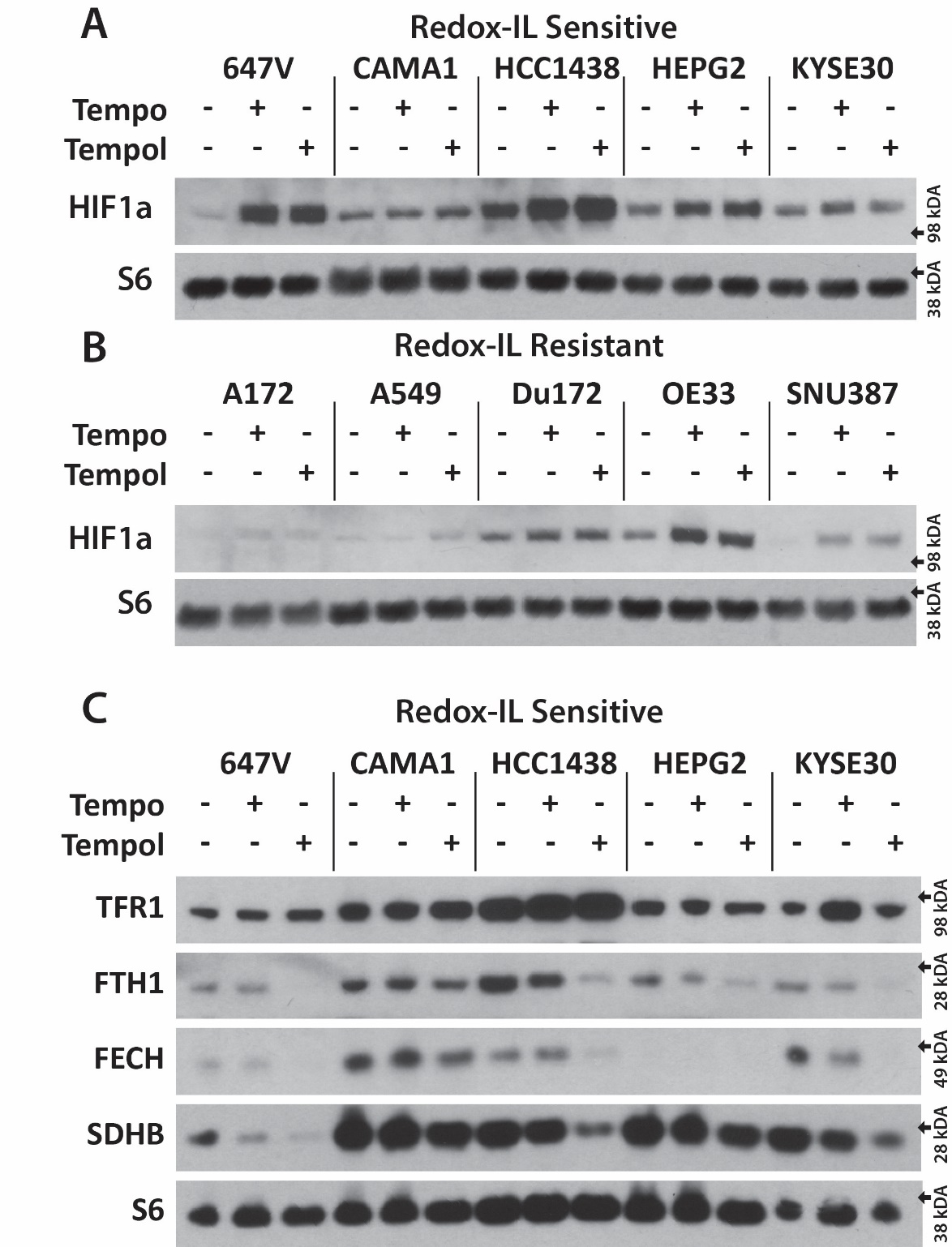
**

**Supp. Figure 13 | Tempo increase HIF1a levels without altering ISC protein levels unlike Tempol. . A, B.** Immunoblots for indicated proteins of lysates derived from redox iron limitation sensitive (A) and resistant (B) cell lines, treated with 250uM Tempo and 250uM Tempol for 2 hours. **C.** Immunoblots for indicated proteins of lysates derived from redox iron limitation sensitive cell lines treated with 250uM Tempo and 250uM Tempol for 4 days

**
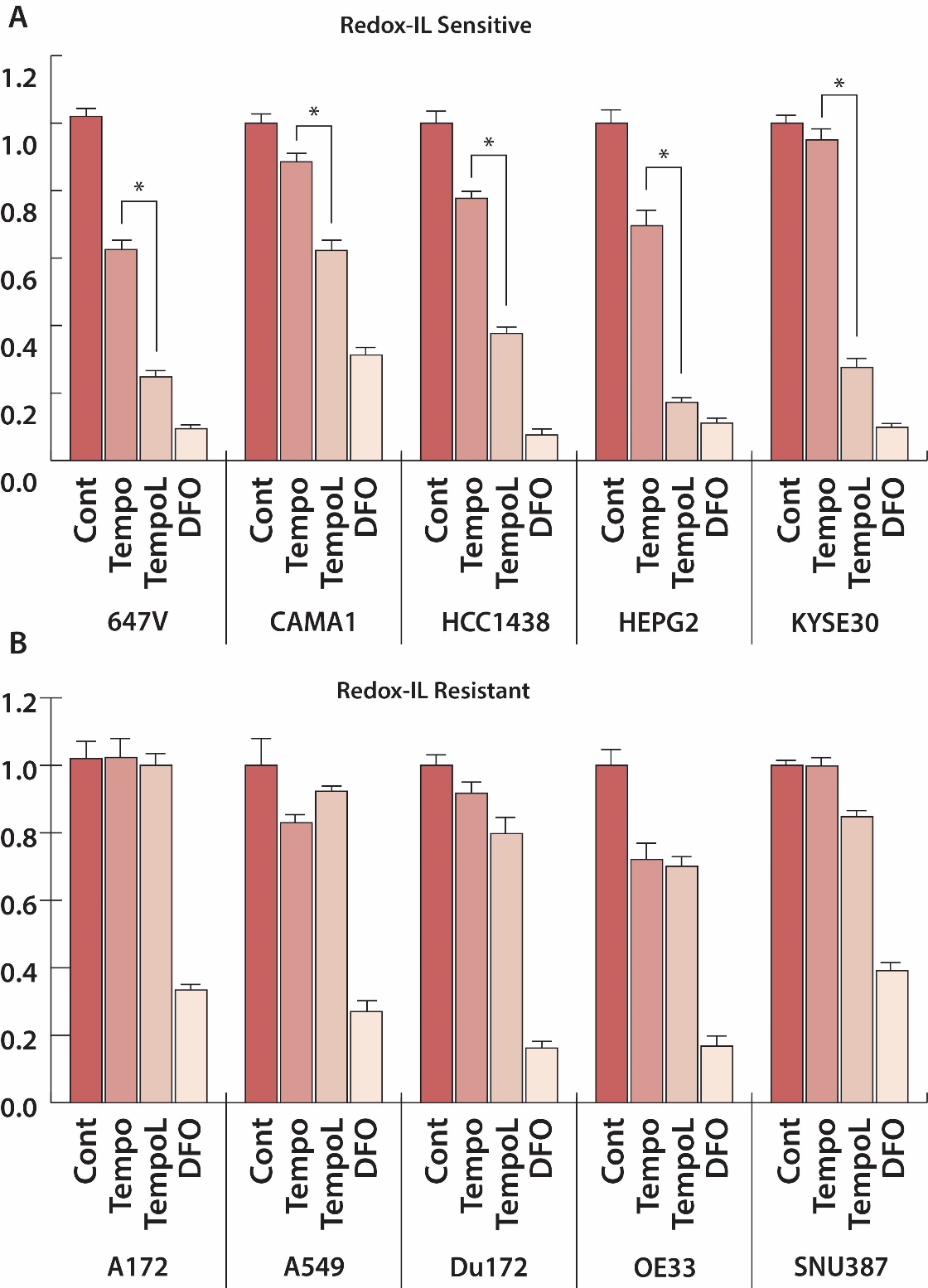
**

**Supp. Figure 14 | Tempo only slightly inhibits proliferation unlike Tempol. A, B** Relative cell count for redox iron limitation sensitive (A) and resistant(B) cell lines, treated with 250uM Tempo and 250uM Tempol, 10uM DFO for 4 days. * p < 0.01, error bars are s.e.m..


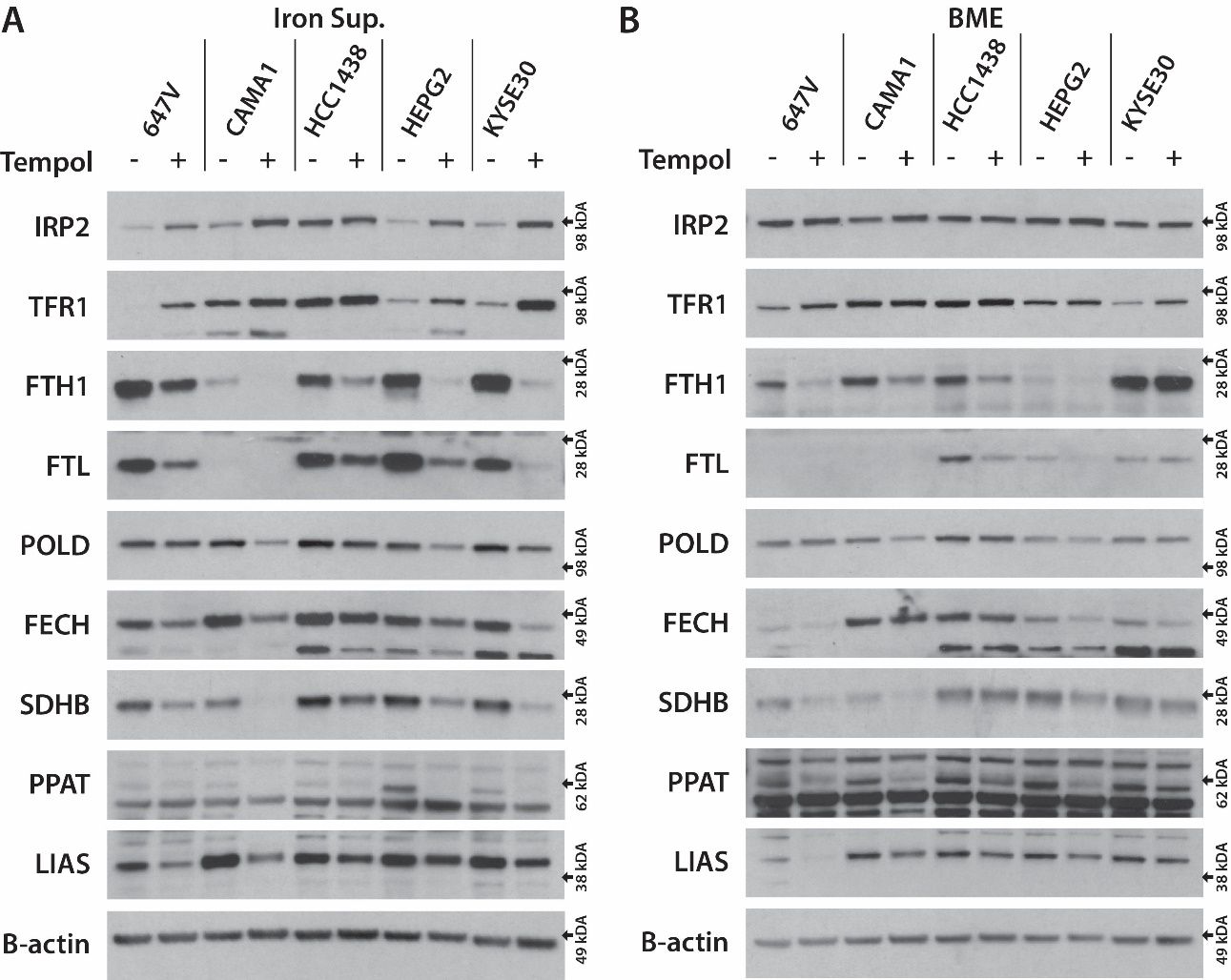


**Supp. Figure 15 | Iron supplementation and BME slightly rescue Fe-S protein degradation by Tempol A,** Immunoblots for indicated proteins of lysates derived from 5 sensitive cell lines, treated with Tempol (Tem., 250uM) and Iron(III) nitrate (Fe, 50uM) for 4 days. **B,** Immunoblots for indicated proteins of lysates derived from 5 sensitive cell lines, treated with Tempol (Tem., 250uM) and 2-Mercaptoethanol (BME, 250uM) for 4 days.
