# Supplementary material for: Redox cycling nitroxide limits cellular iron availability and selectively inhibits iron-sulfur cluster metabolism
Source: Cell Death Discov. 2026 Mar 24;12:165. doi: 10.1038/s41420-026-03042-w (PMC13039289; doi:10.1038/s41420-026-03042-w)
Supplement: Supplementary file 2 — Uncropped blot and gel images [file 41420_2026_3042_MOESM2_ESM.pdf]

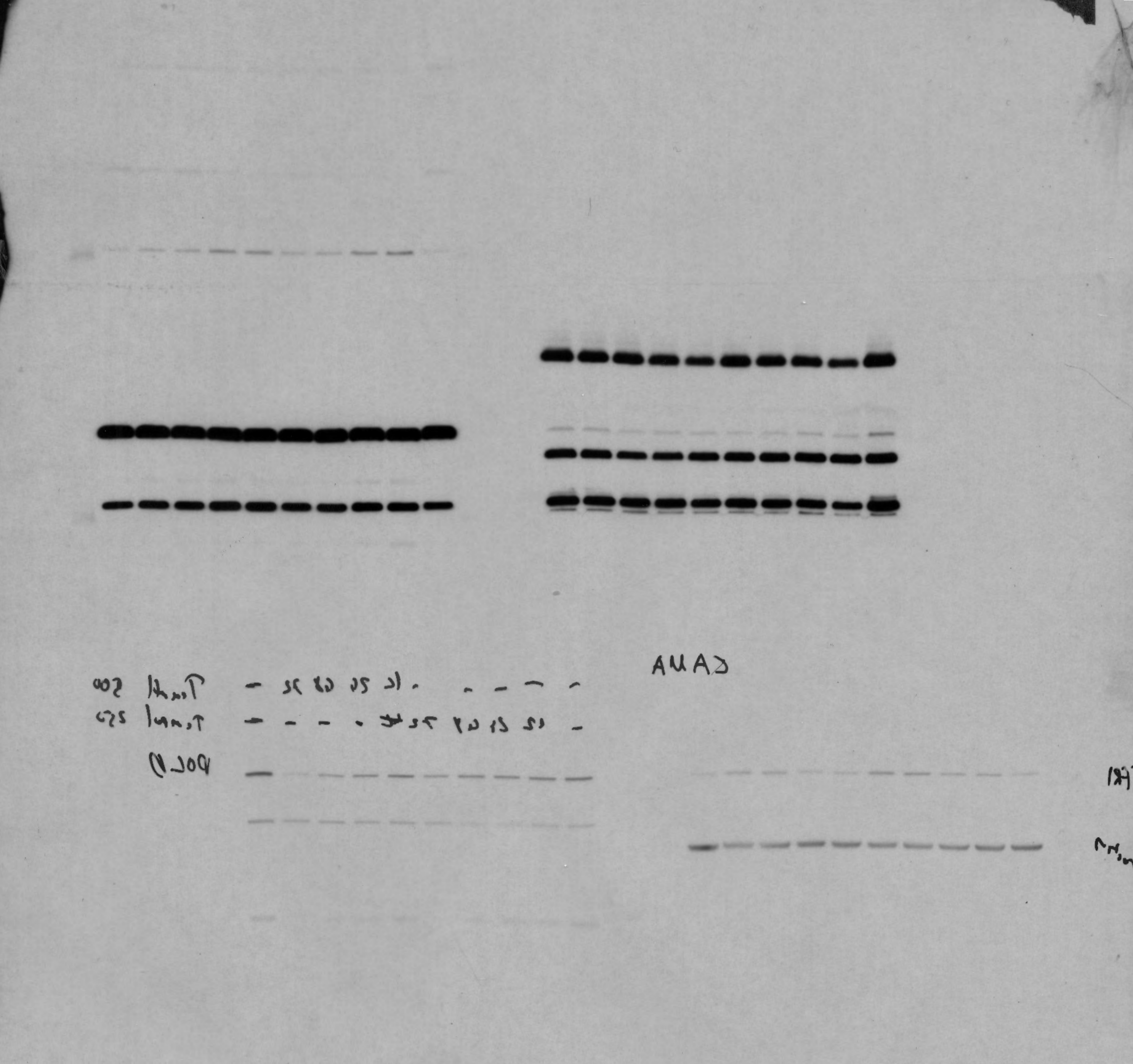

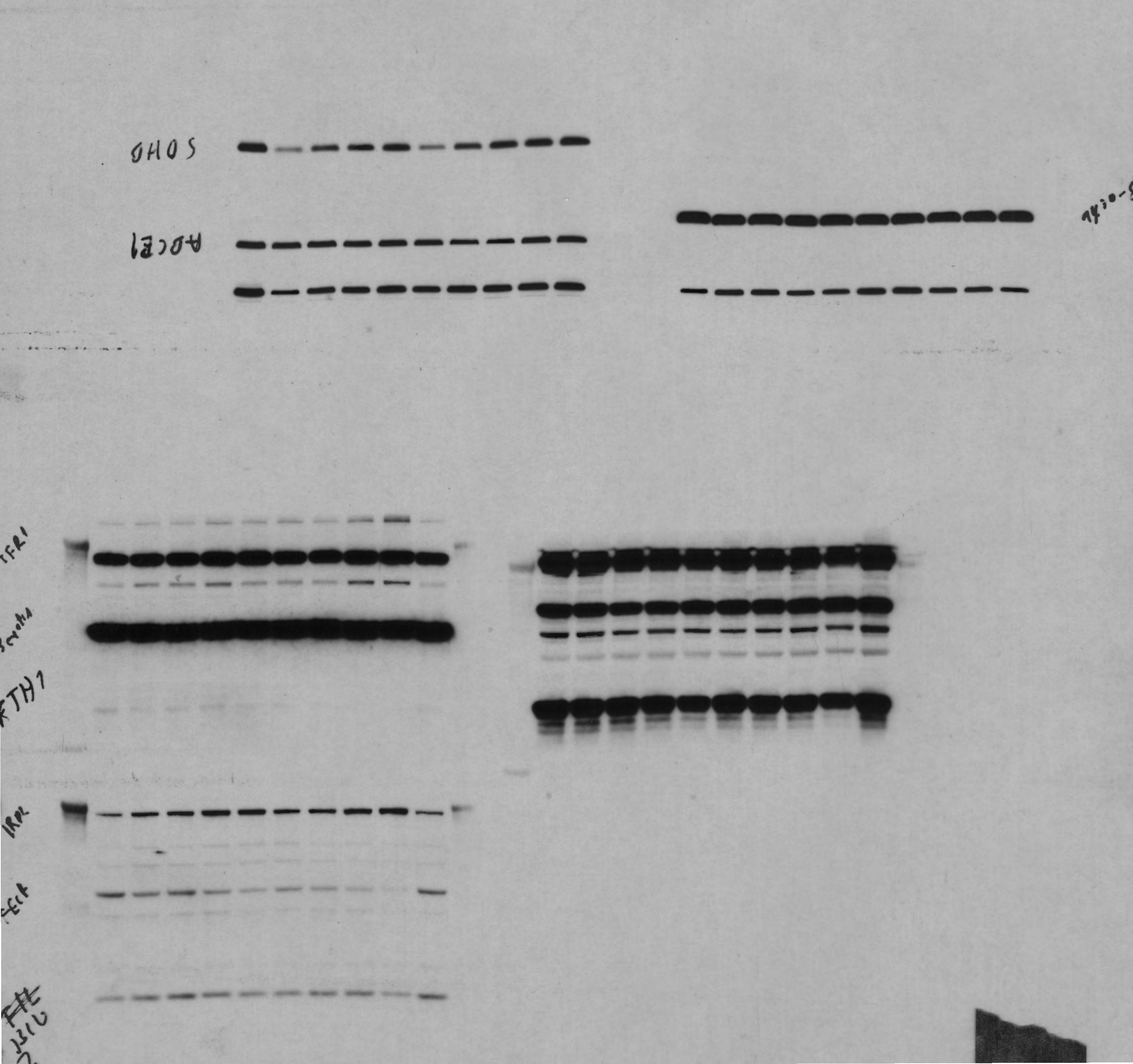

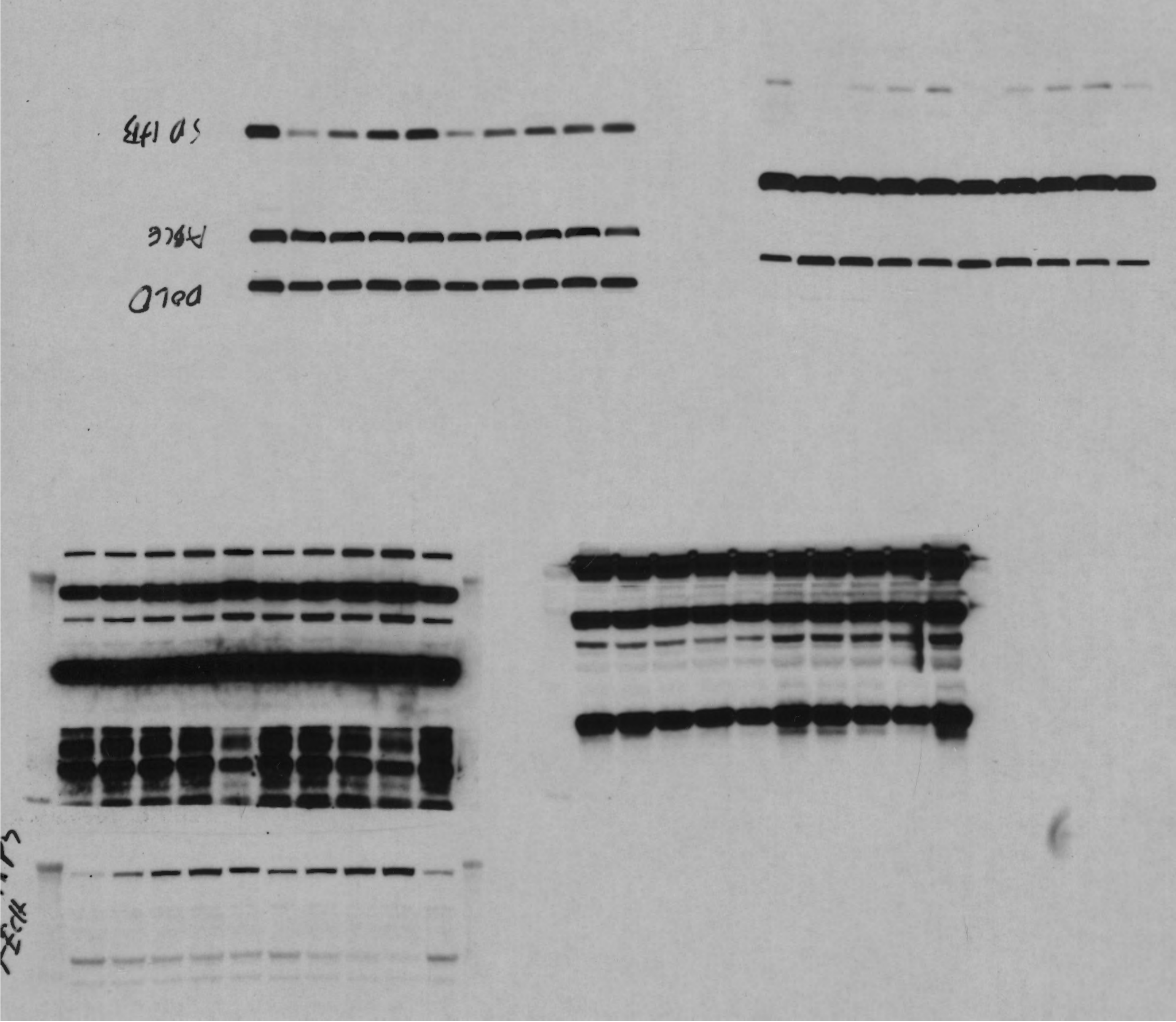

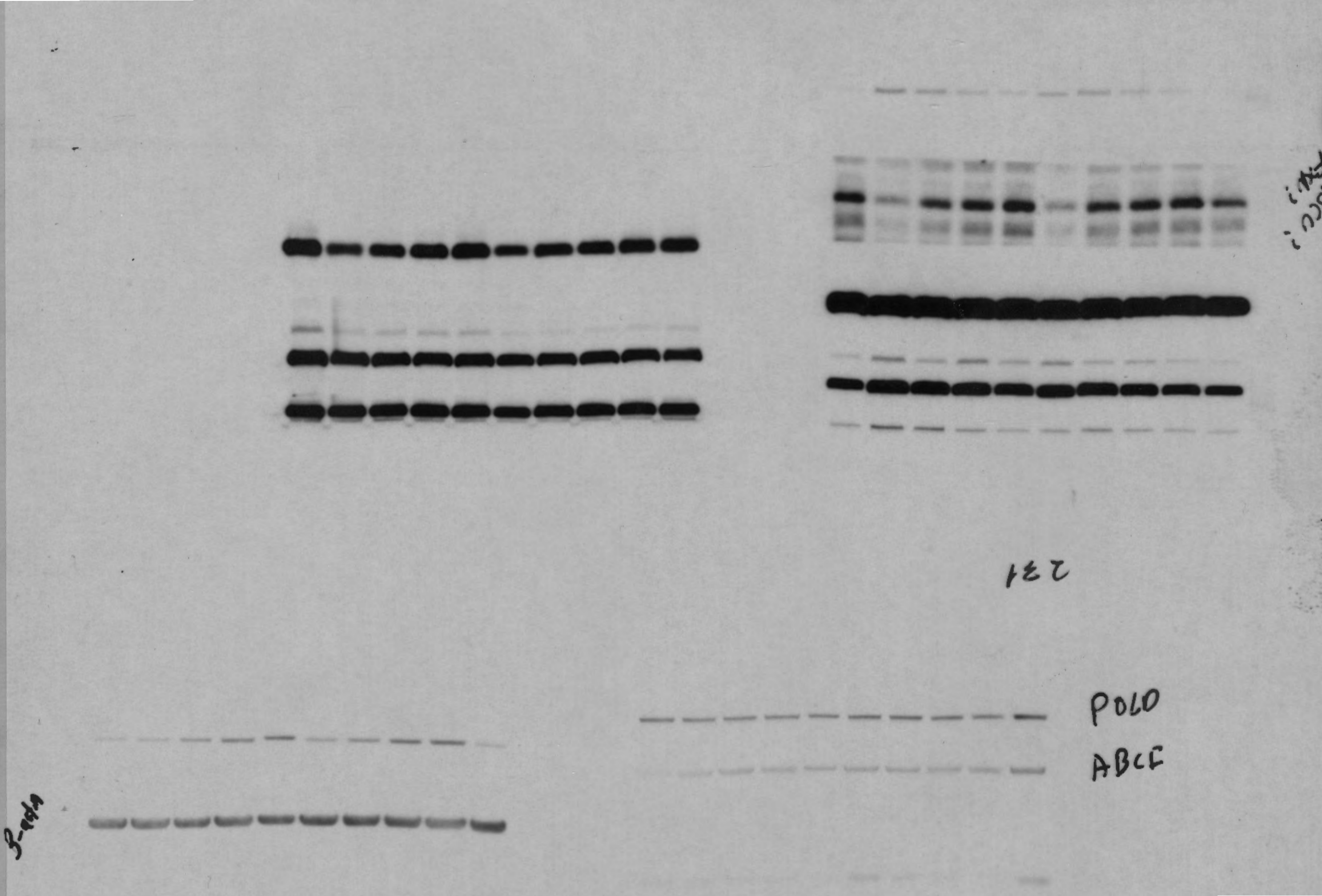
POLO
ABCE

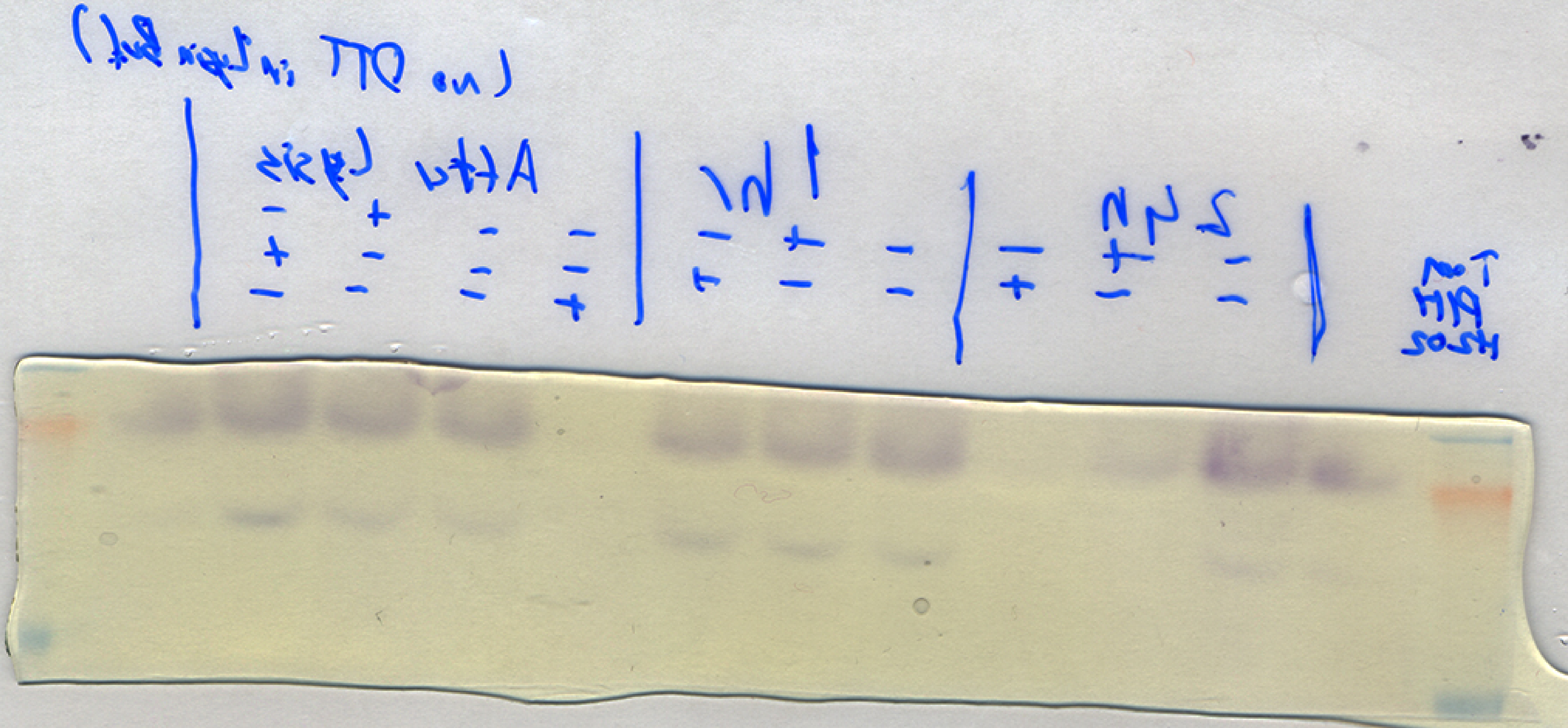
(no DTT in Lysis Buf)
Lysis
1hr
2hr
H2O2

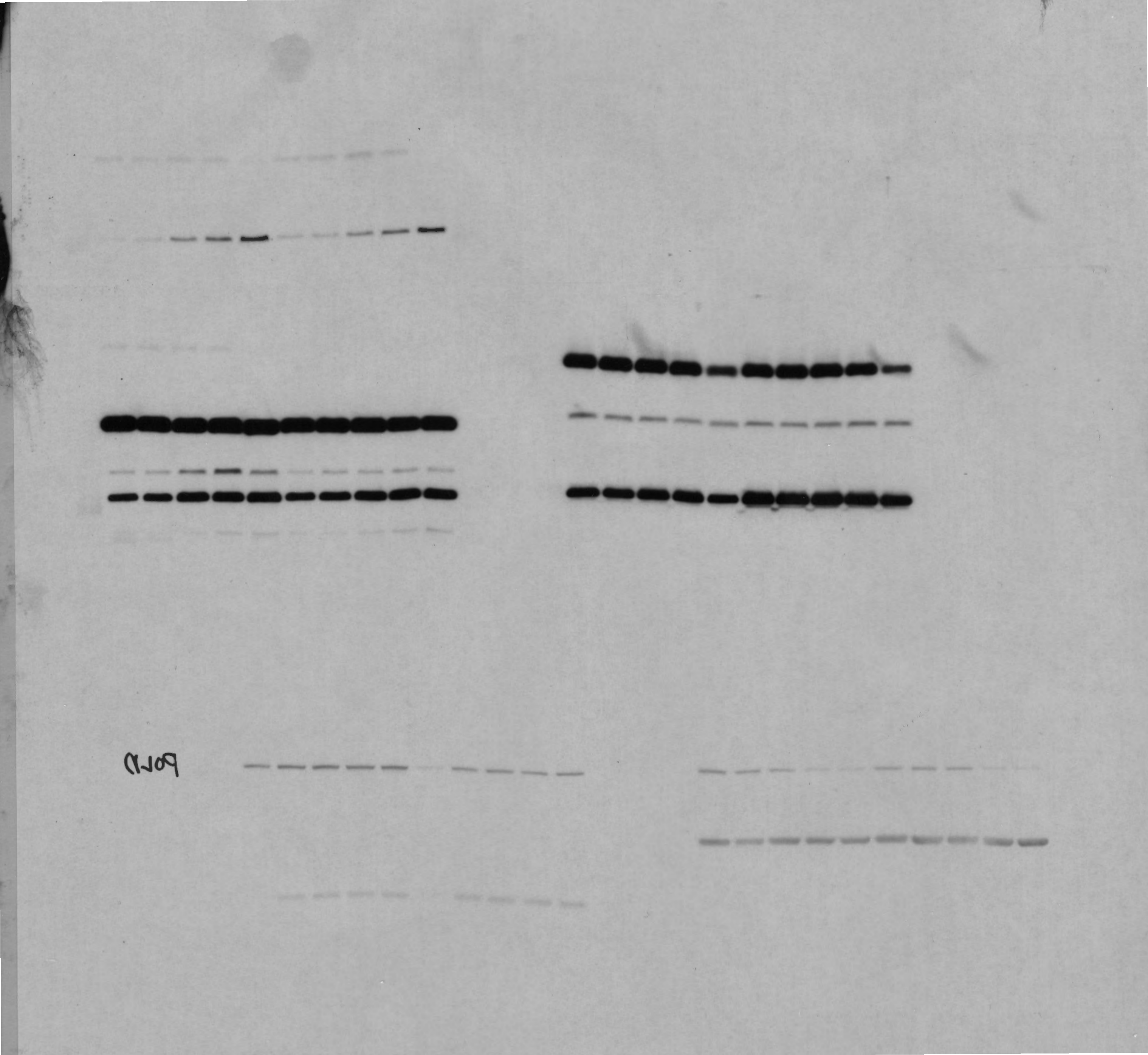

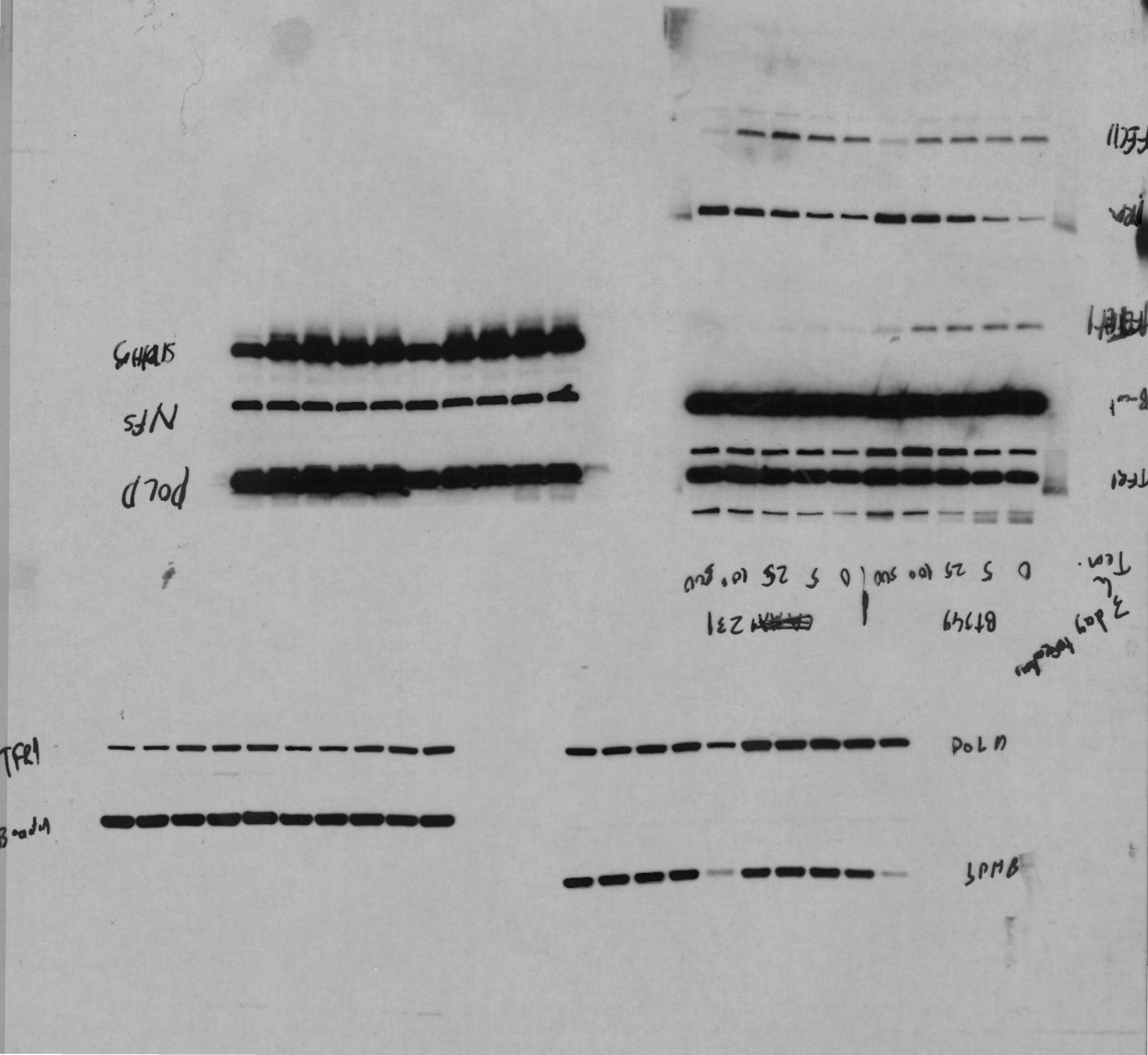
TFR1
POL D

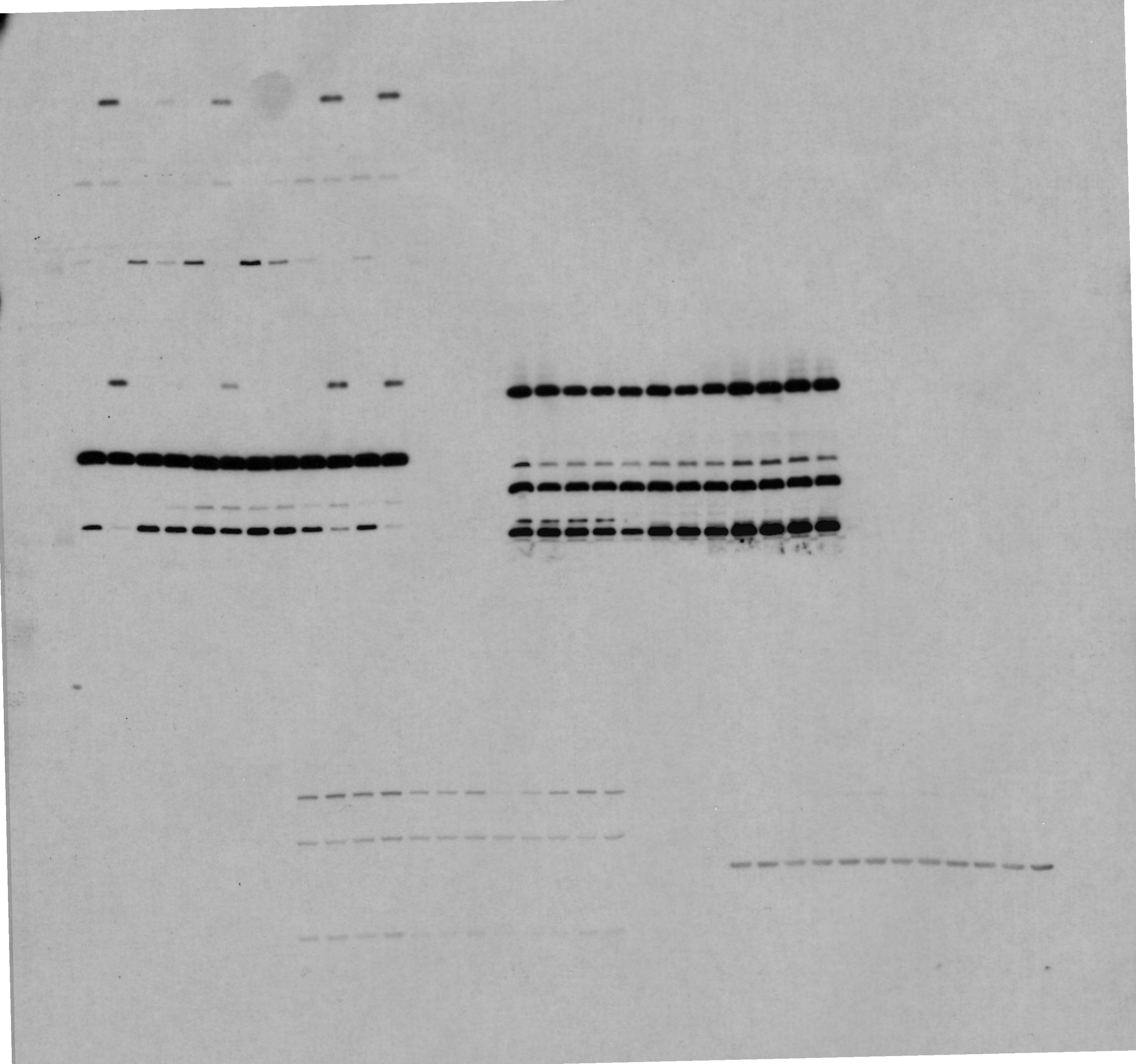

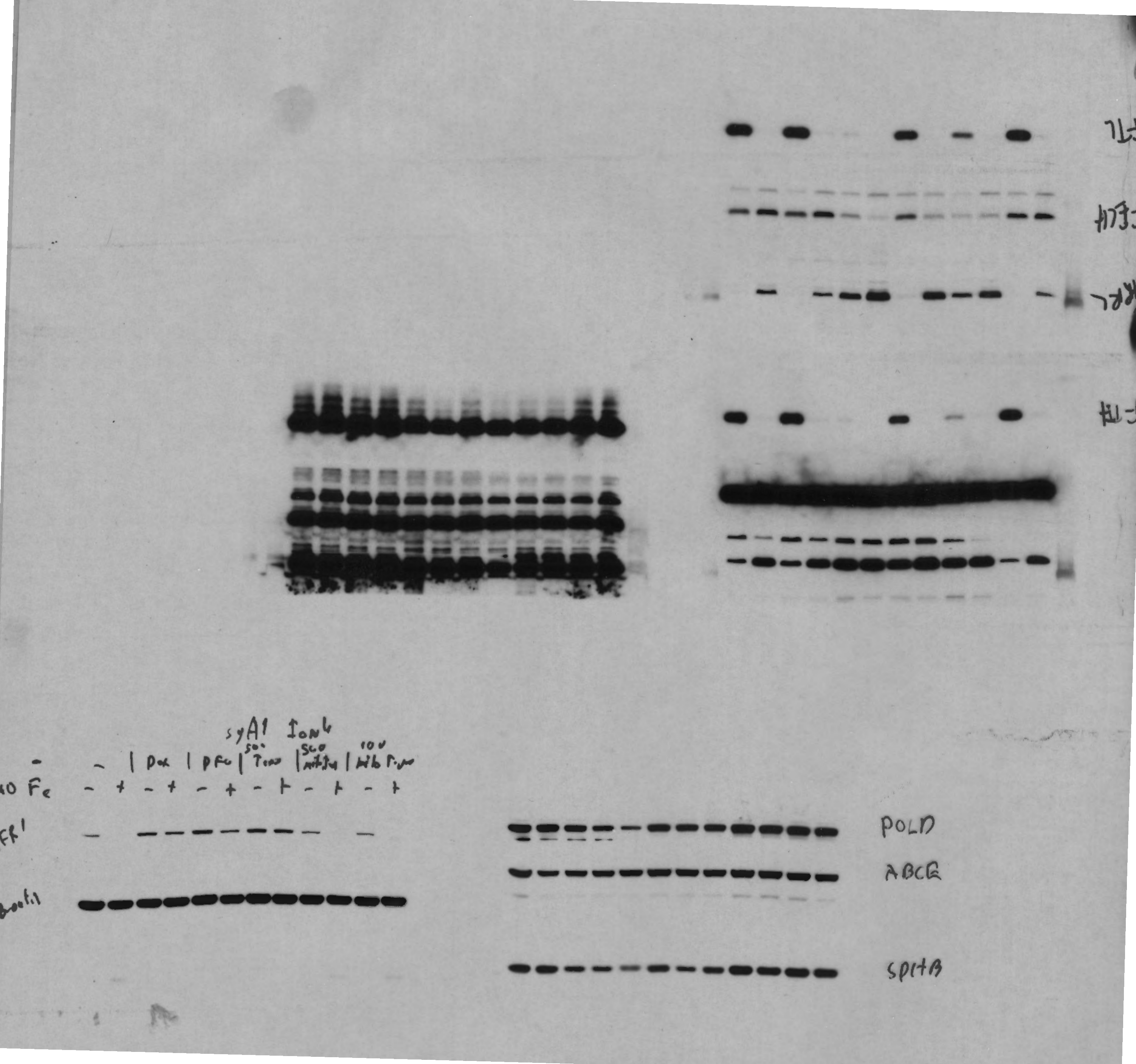
POLD
ABCE
SPItB

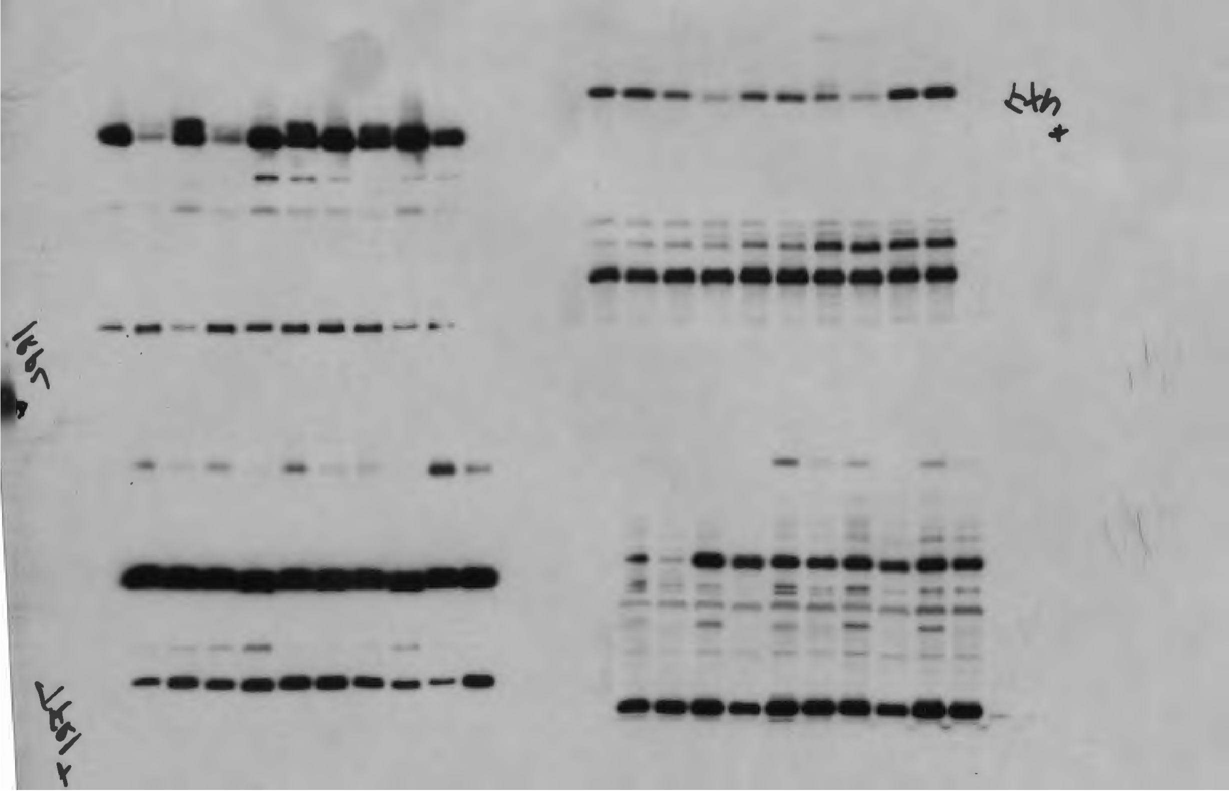

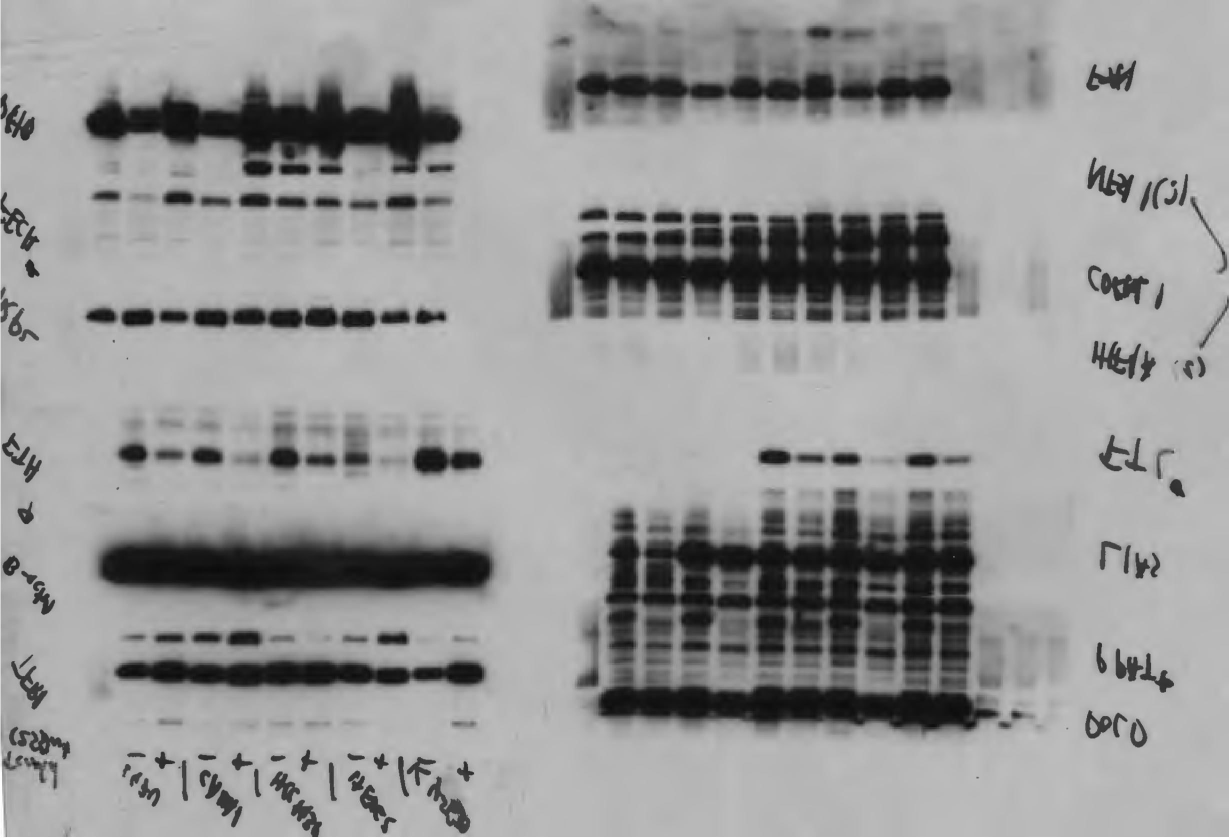

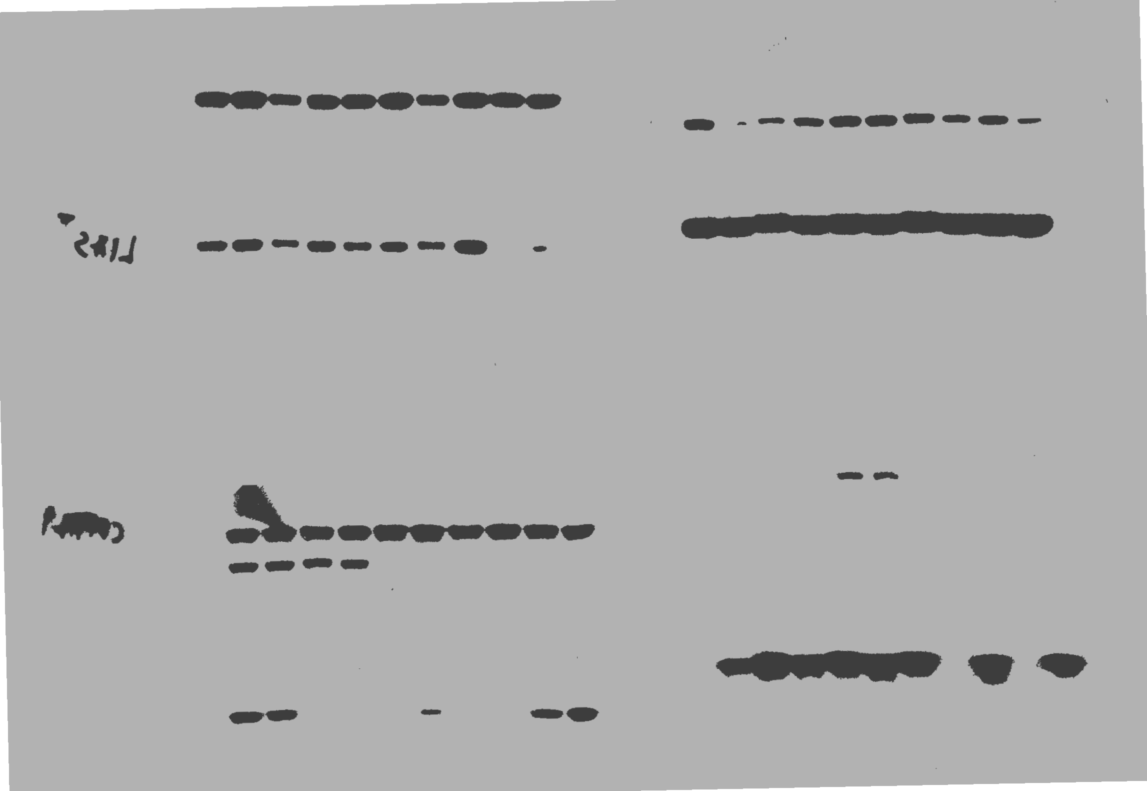

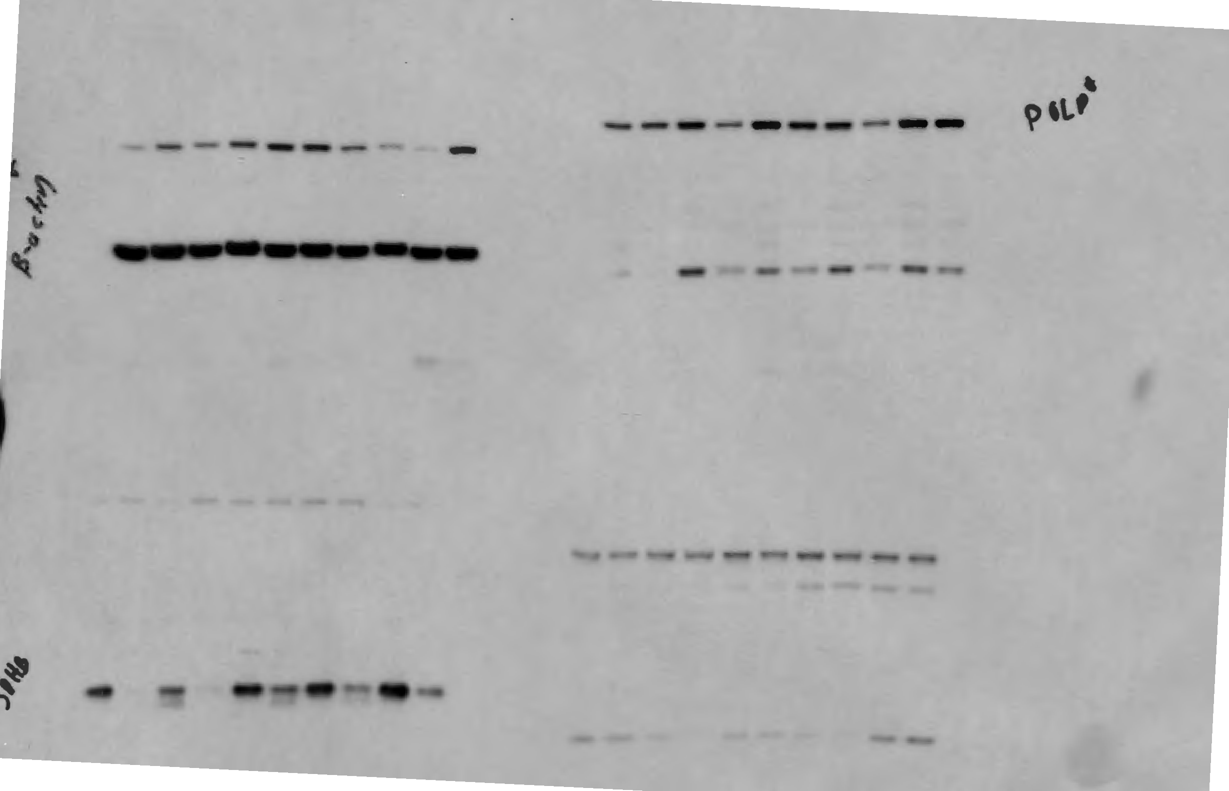
B-actin

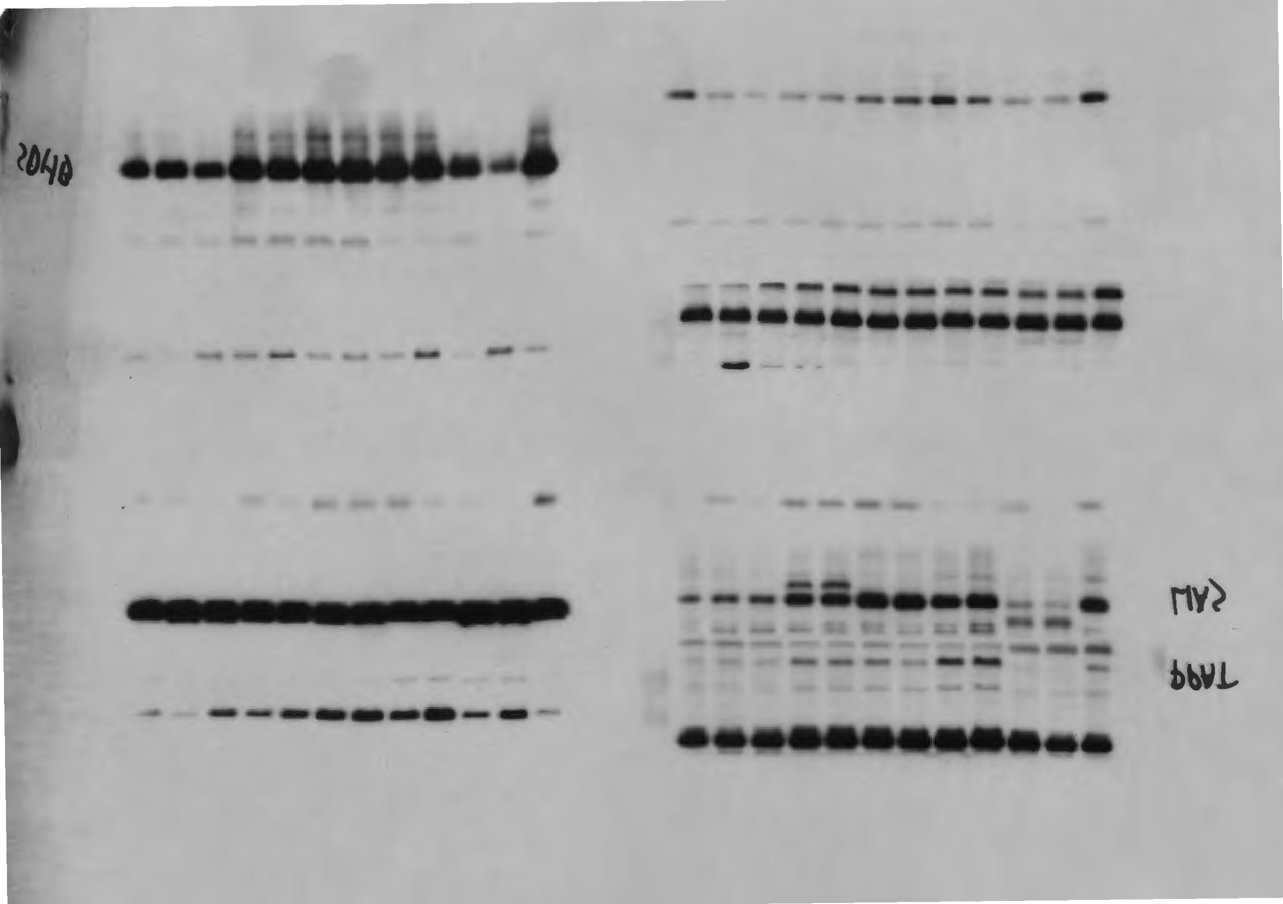

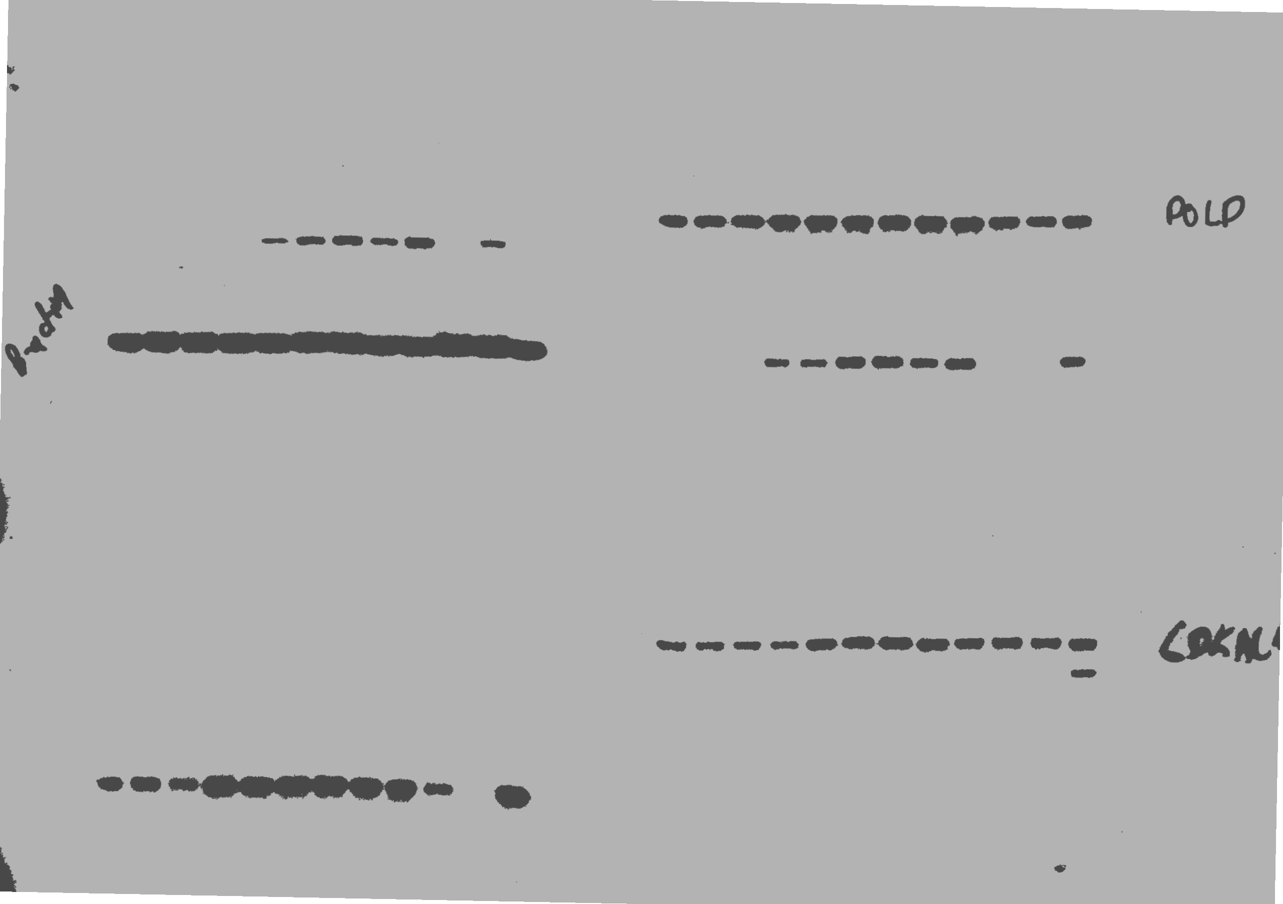
POLD

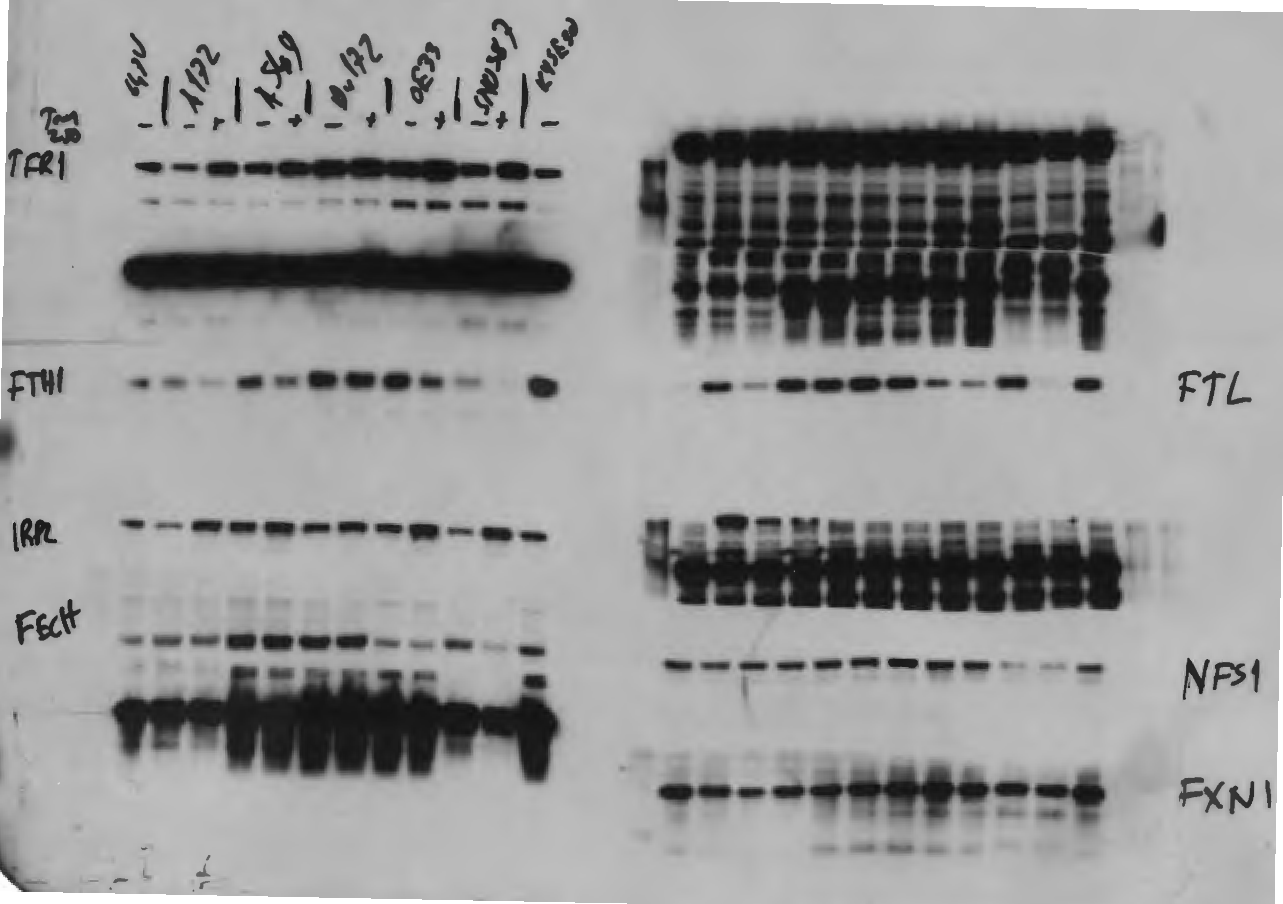
TFR1
FTH1
IRP2
FECH
FTL
NFS1
FXN1

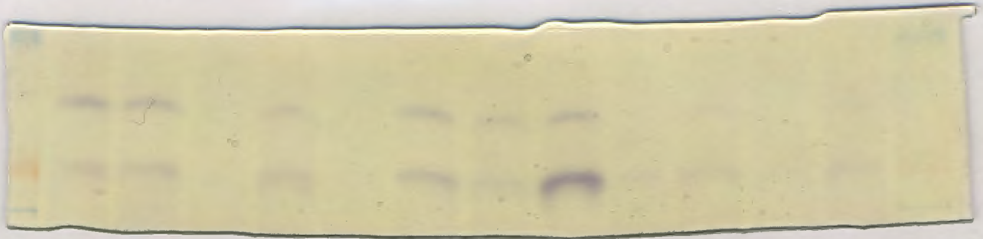

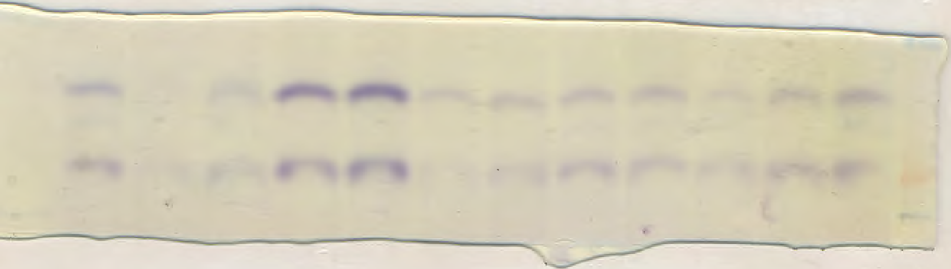

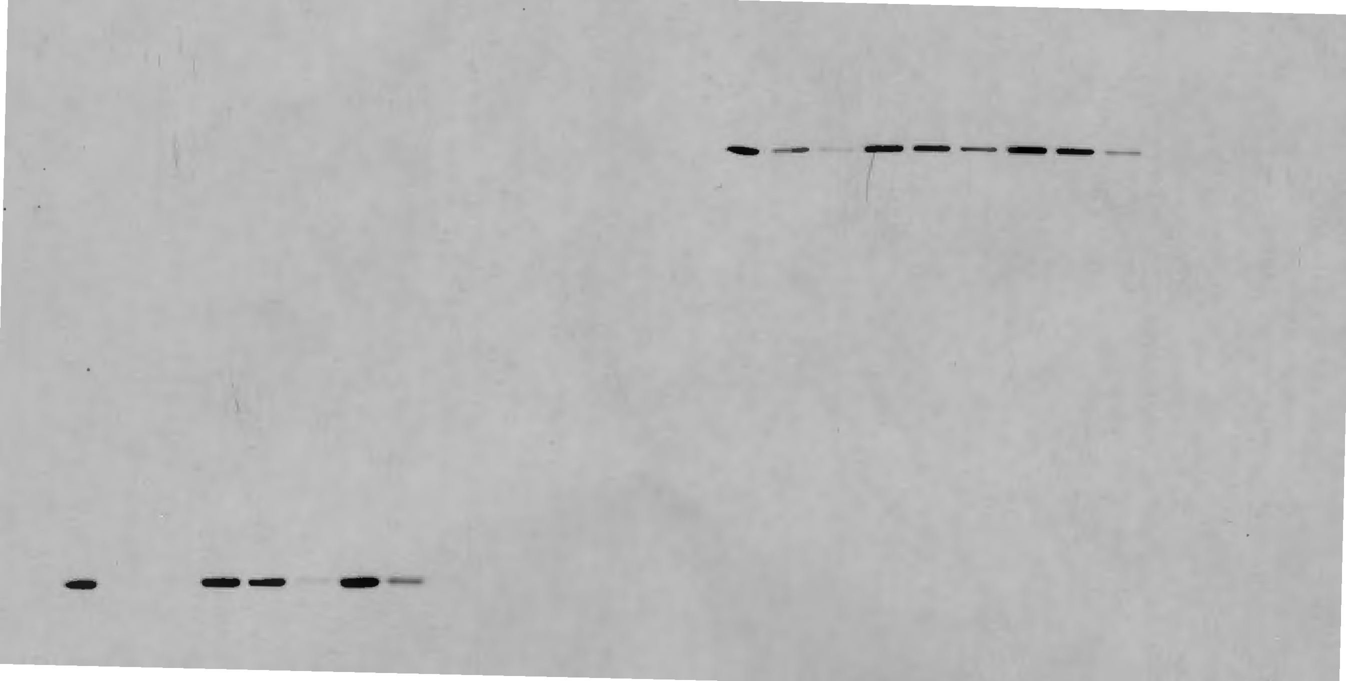

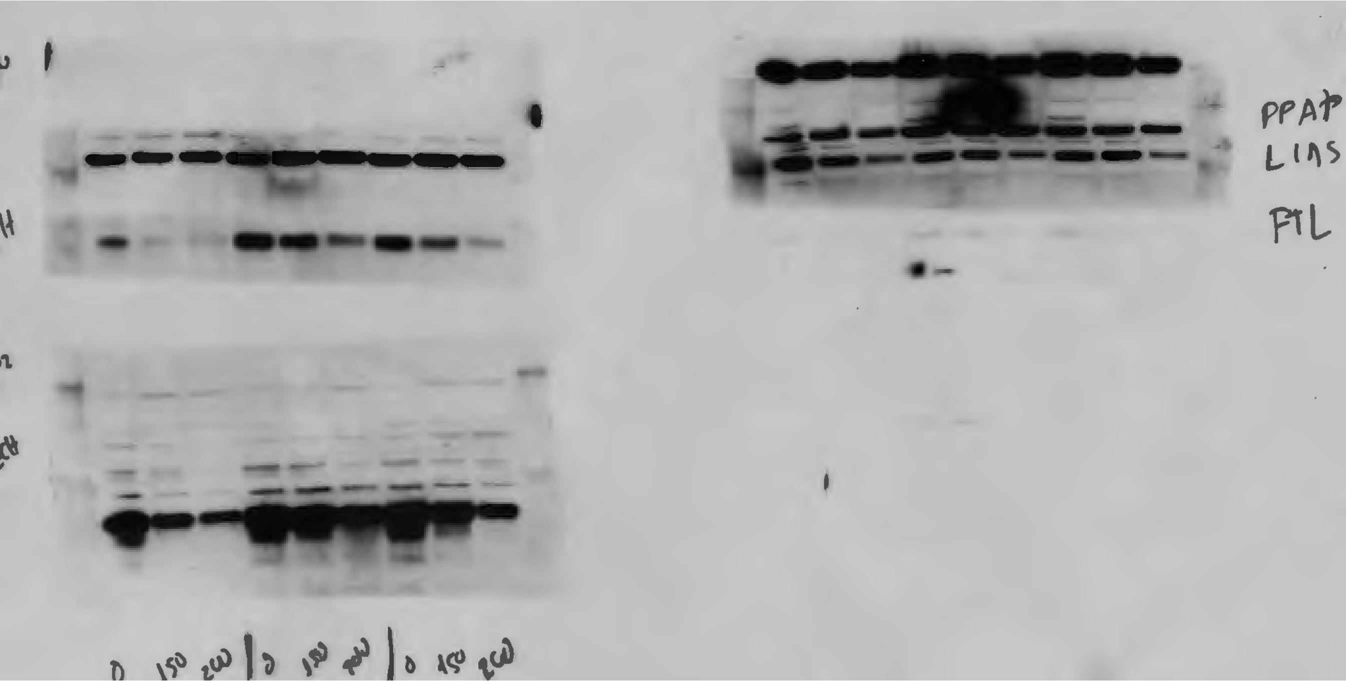
PPAP
LINS
FTL
0 150 200 | 0 150 200 | 0 150 200

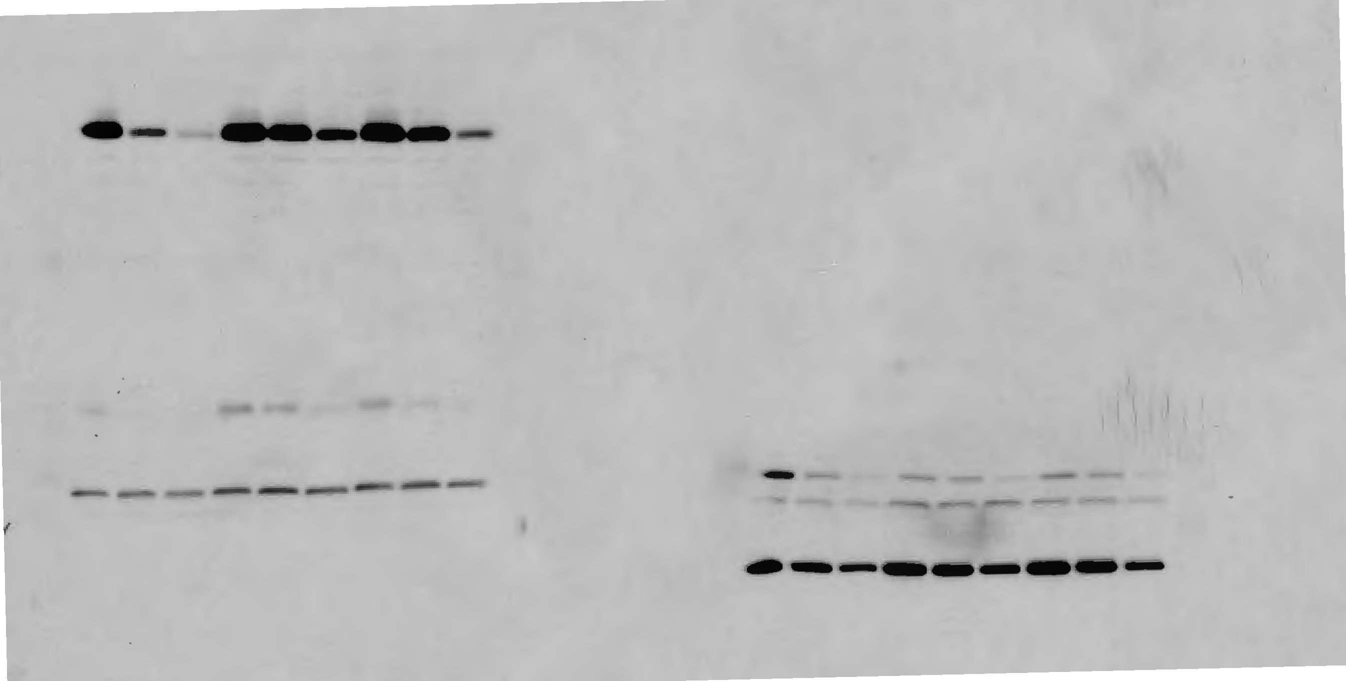

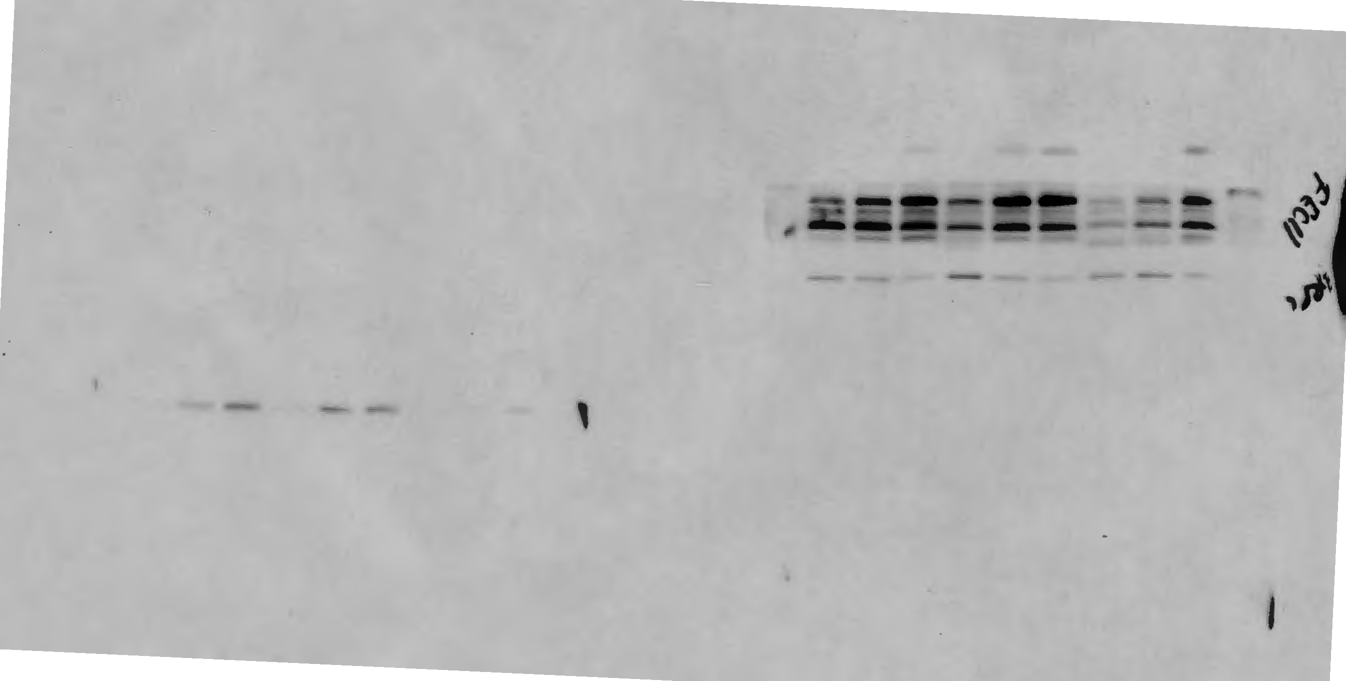

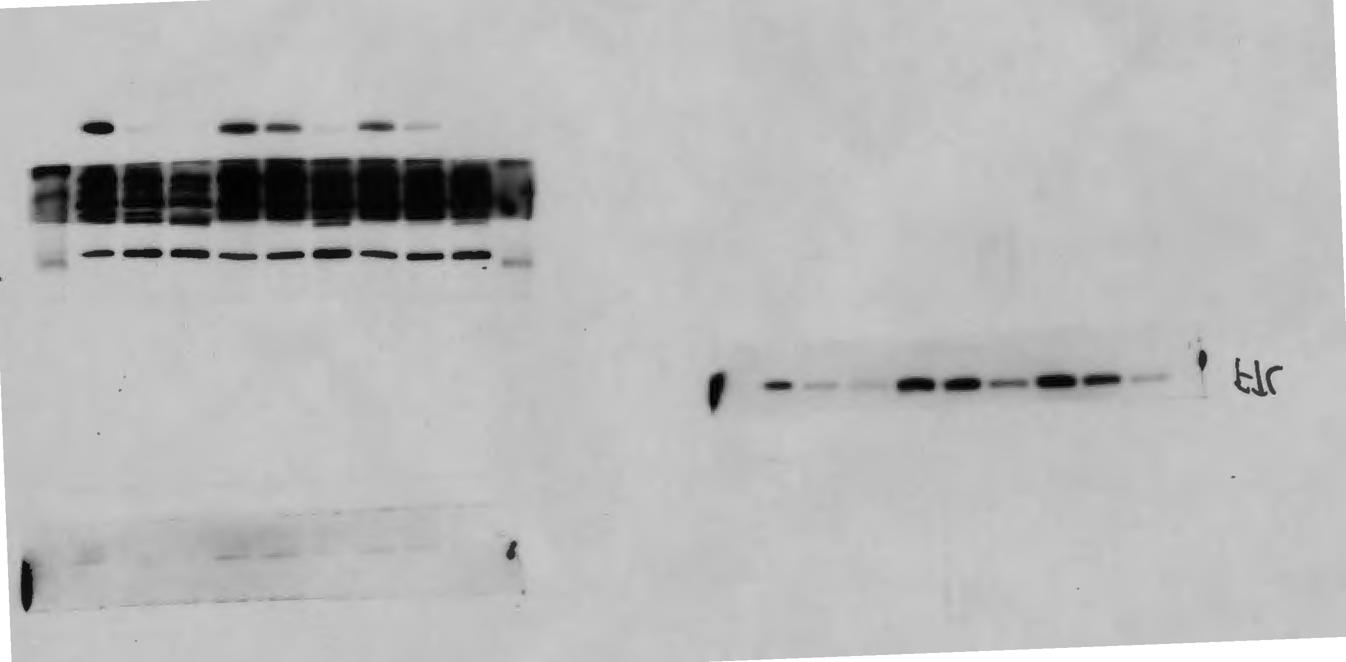

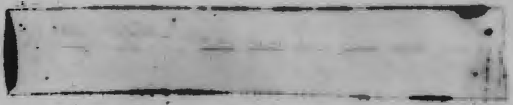

FLAG

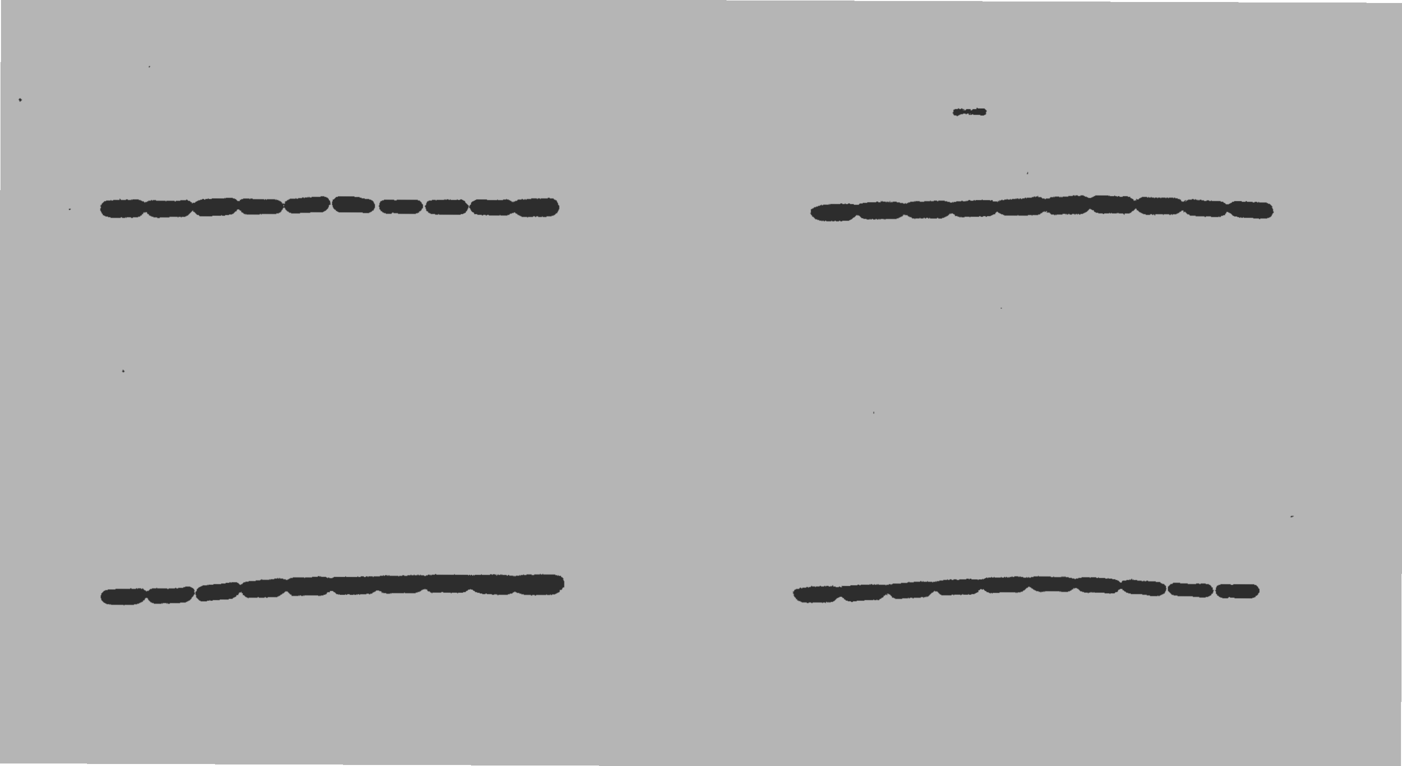

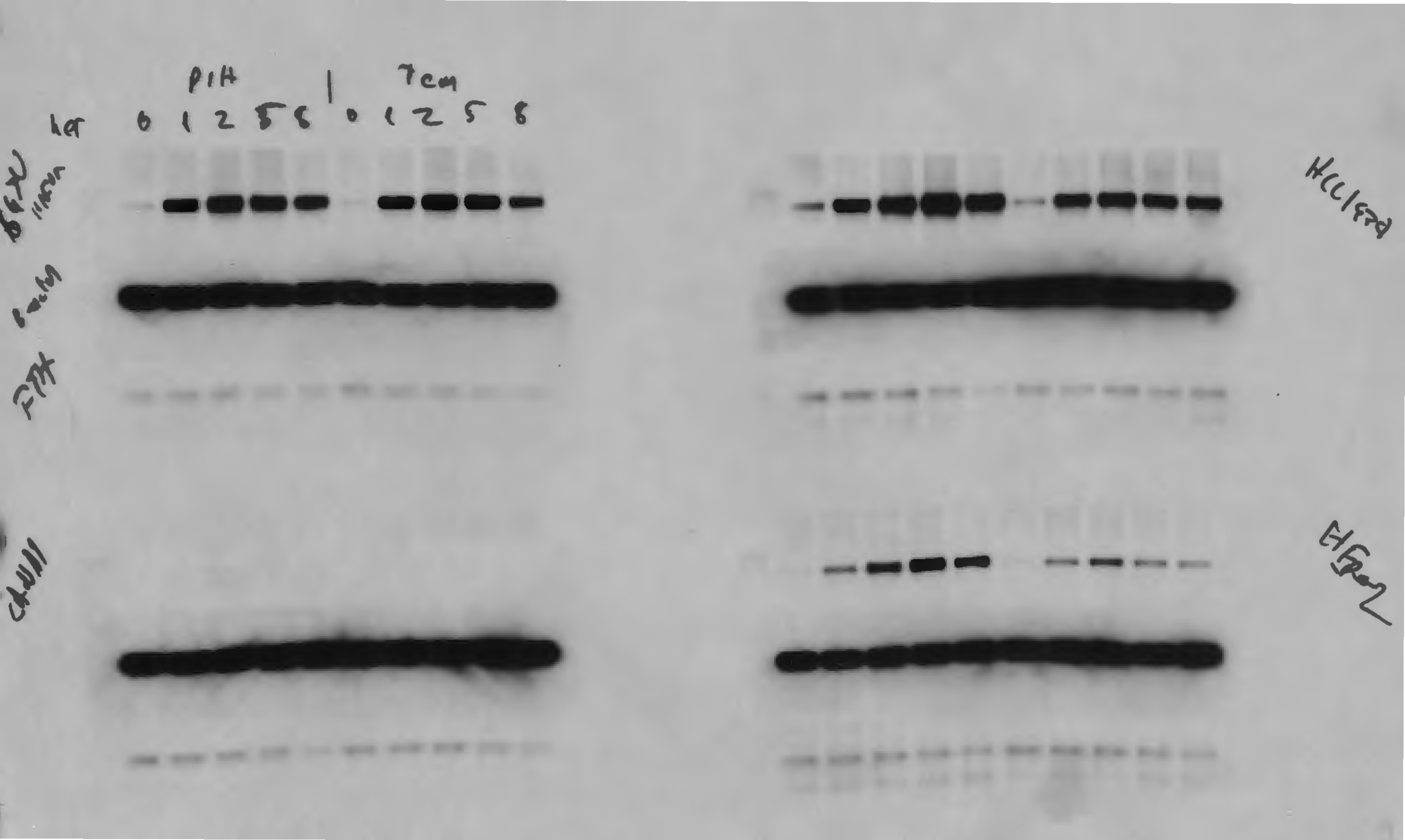
PIH
hr 0 1 2 5 8 0 1 2 5 8

[redacted] [redacted]

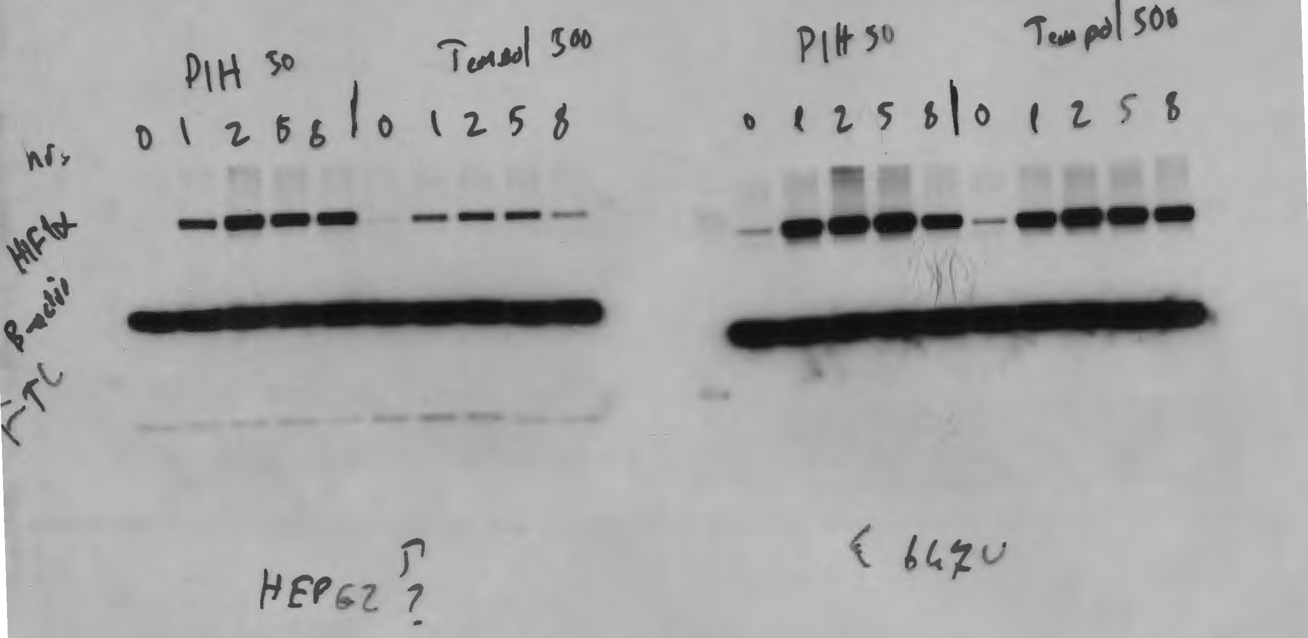
PIH 50
Tempol 500
0 1 2 5 8 | 0 1 2 5 8
PIH 50
Tempol 500
0 1 2 5 8 | 0 1 2 5 8
hrs
HIF1α
β-actin
HEPG2 ?
6642U

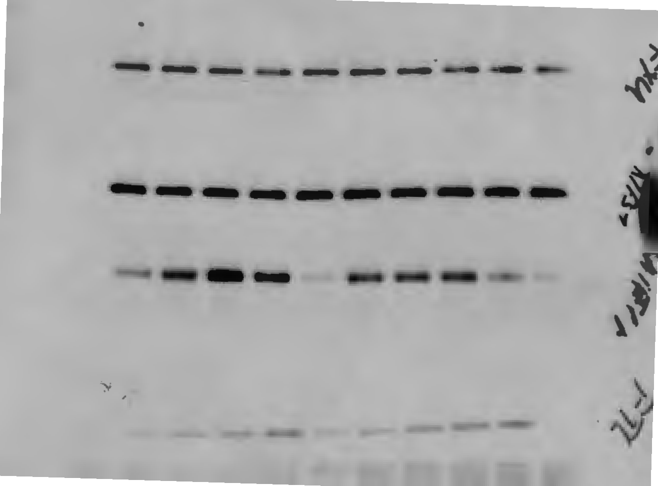

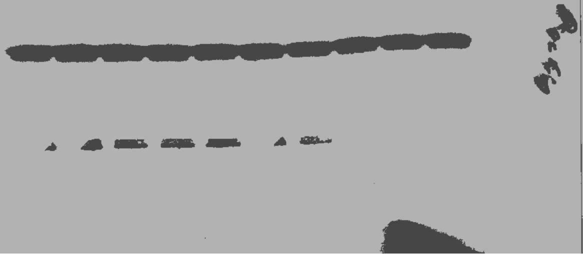

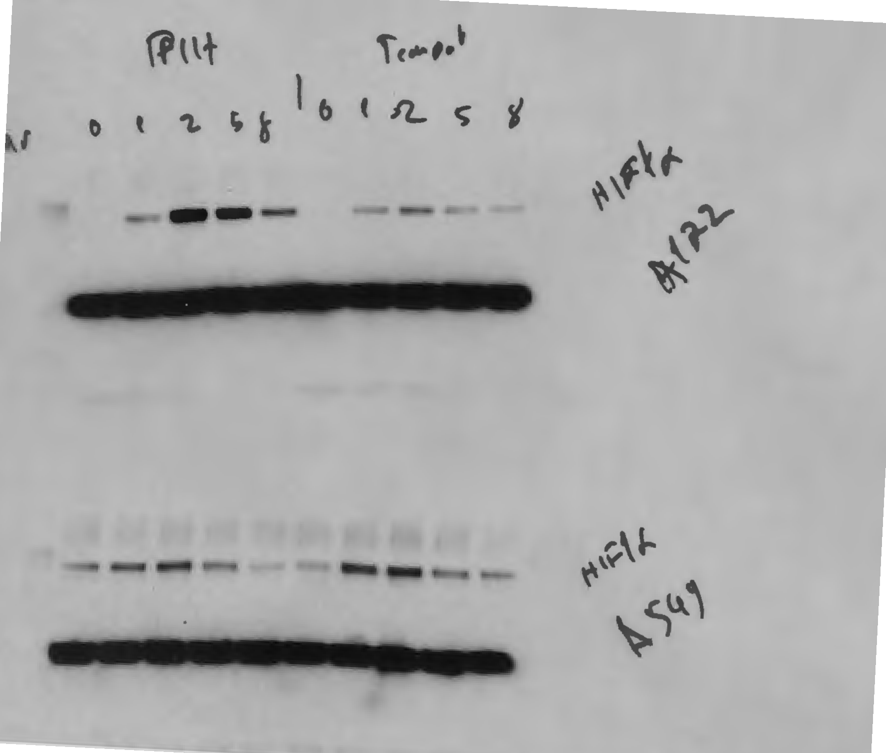
0 1 2 5 8
0 1 2 5 8
HIF1α
HIF1α
A549

[redacted]

[redacted]

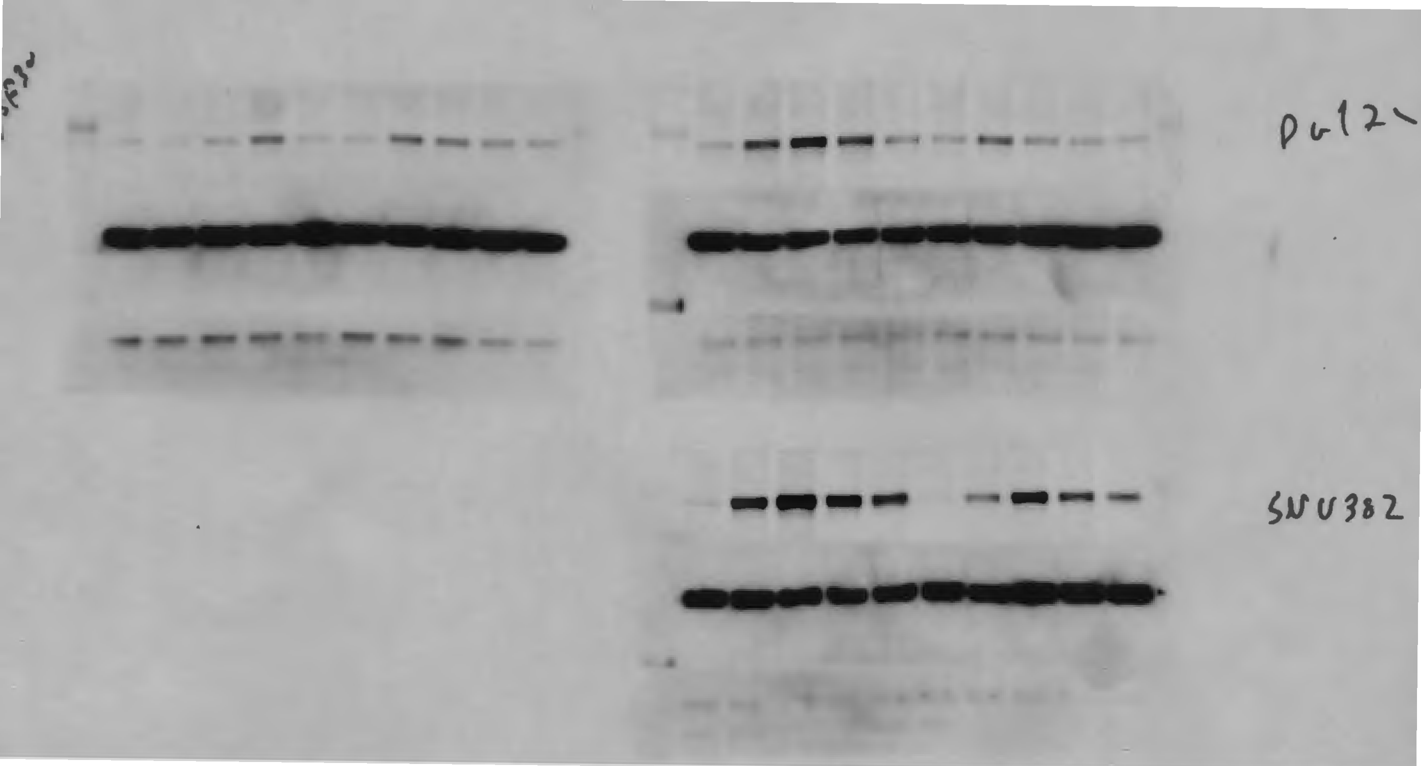
SNU382

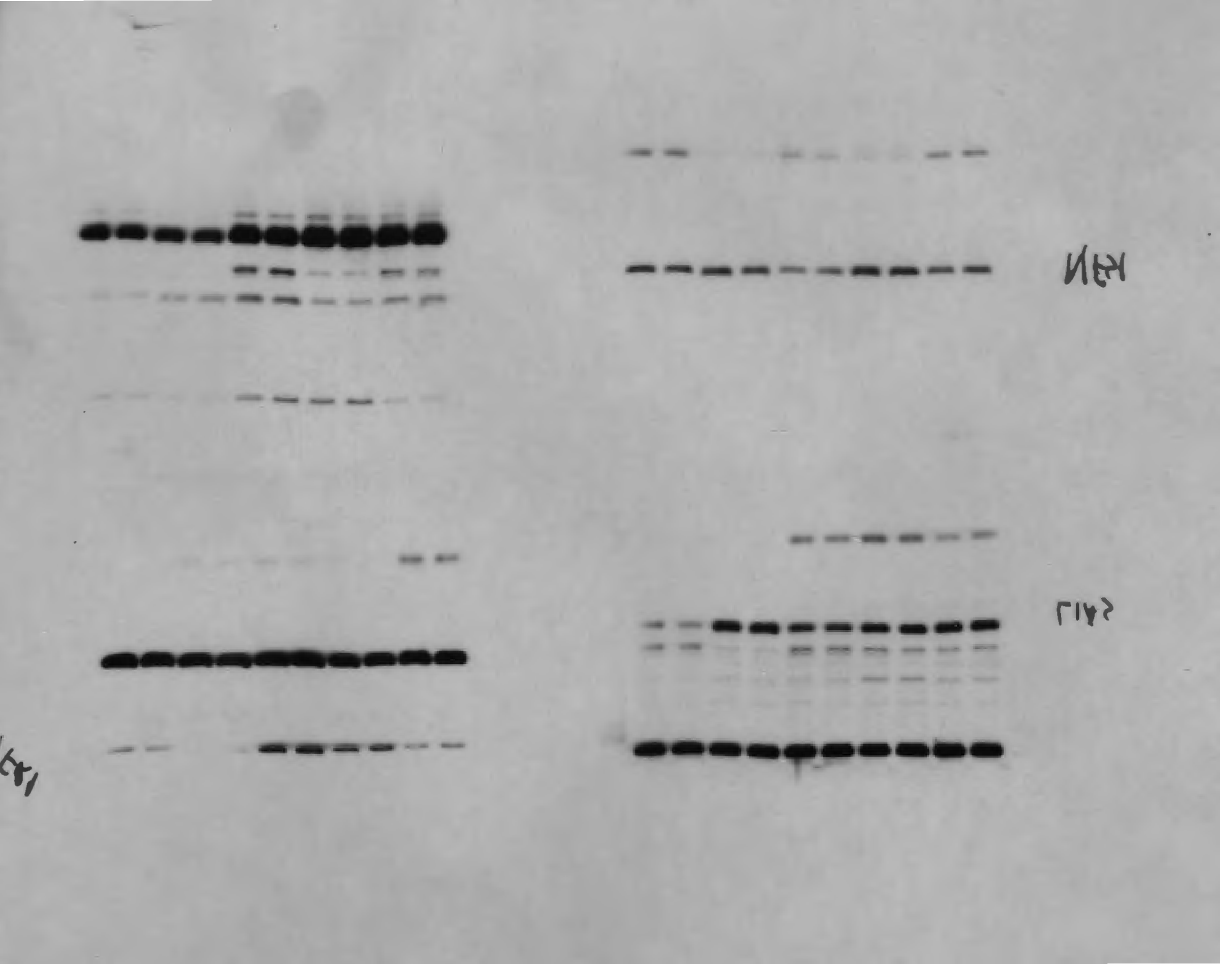

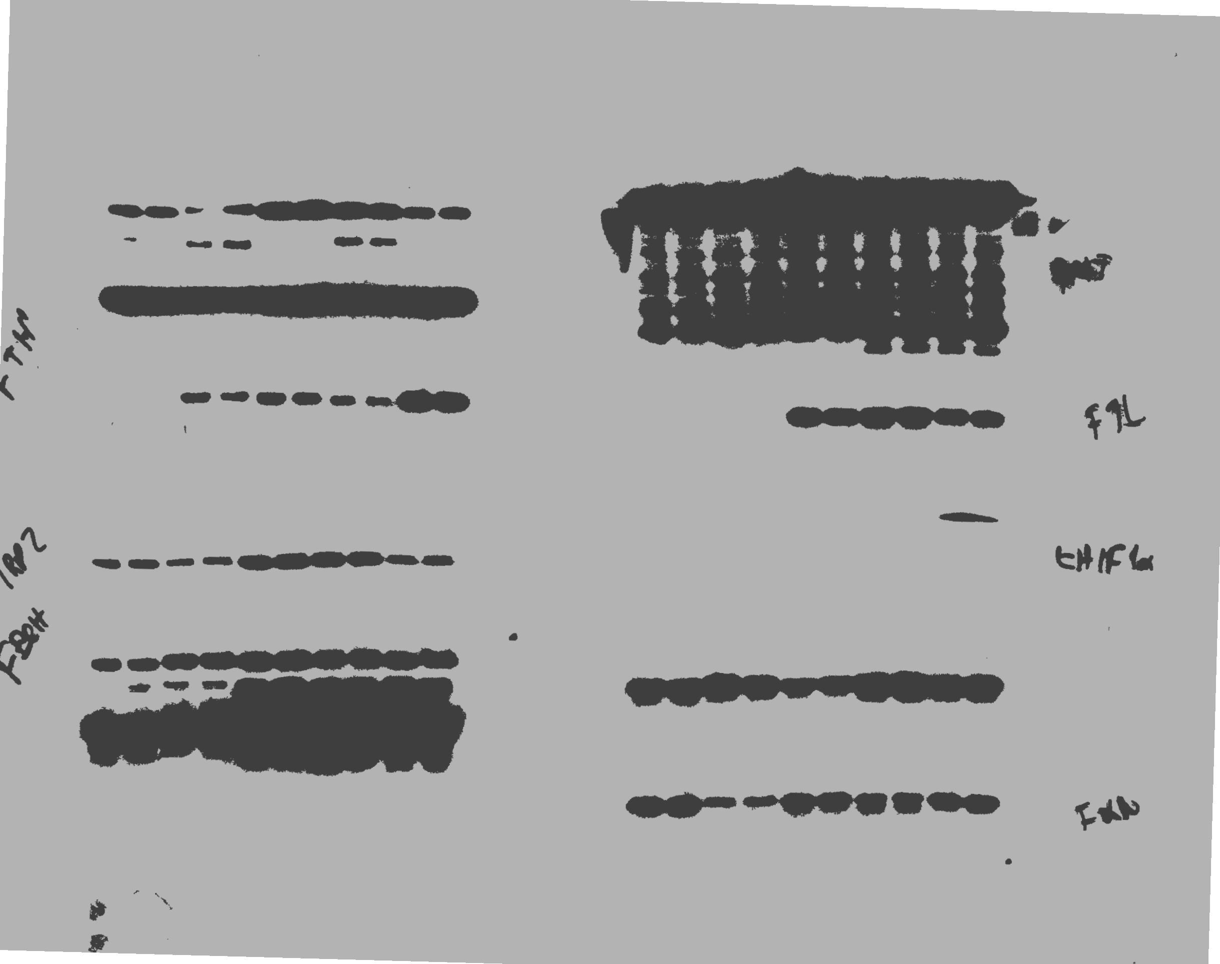

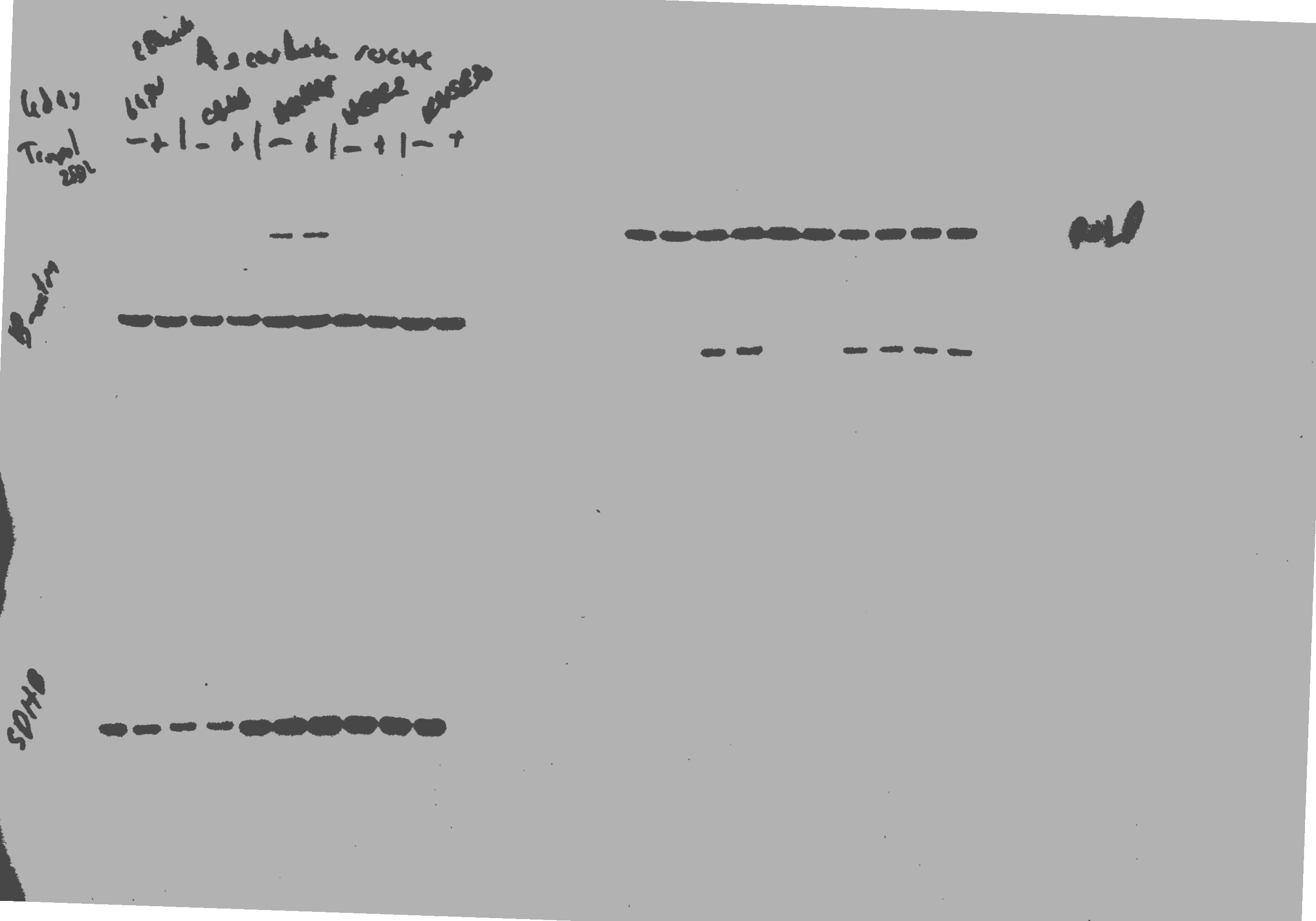
Tempol
SDHB

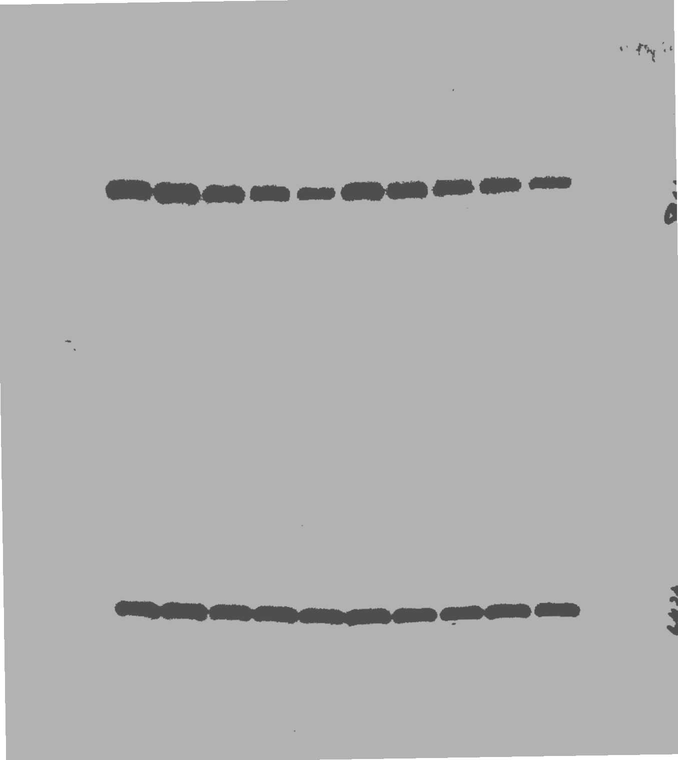

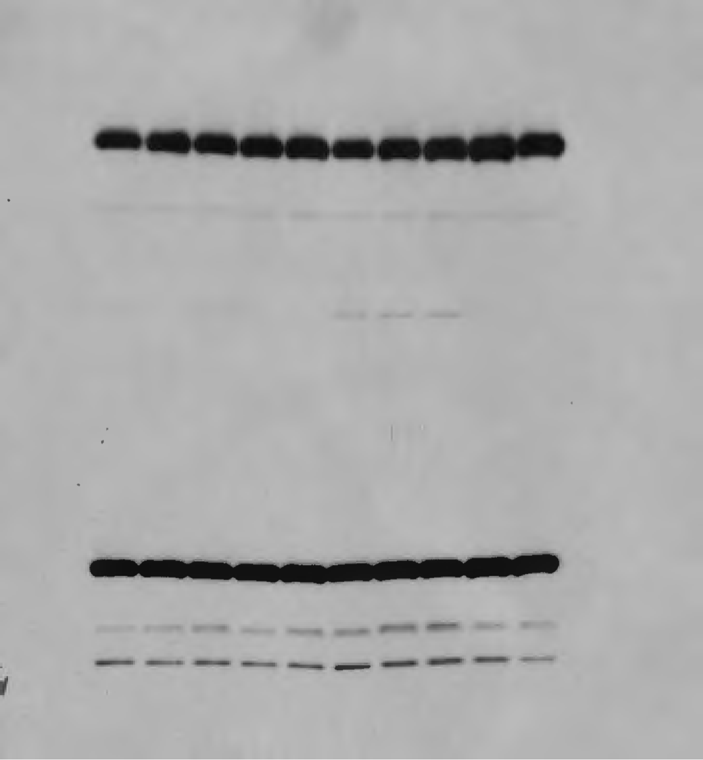

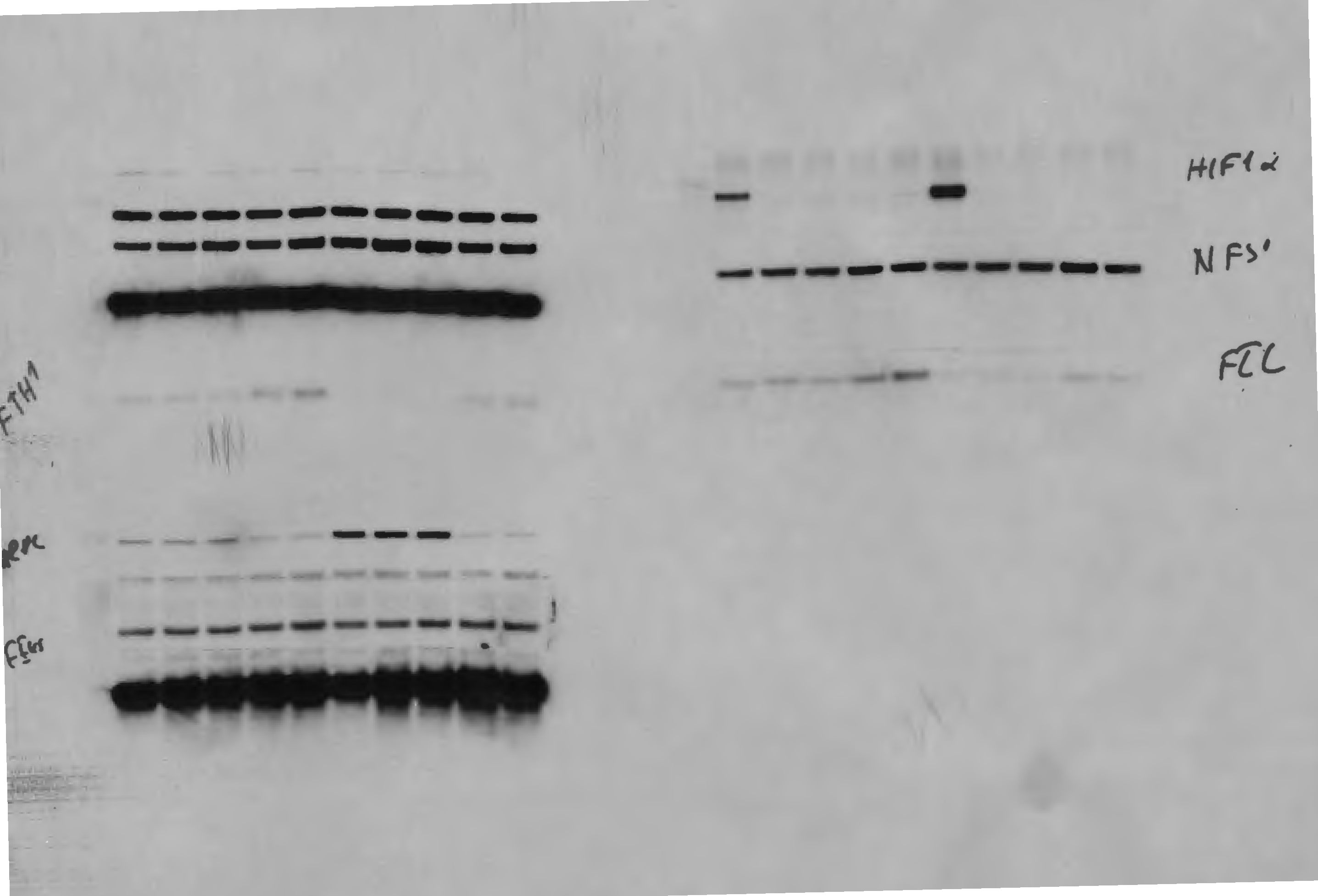

HIF1α
NFS1
FTL
FTH1

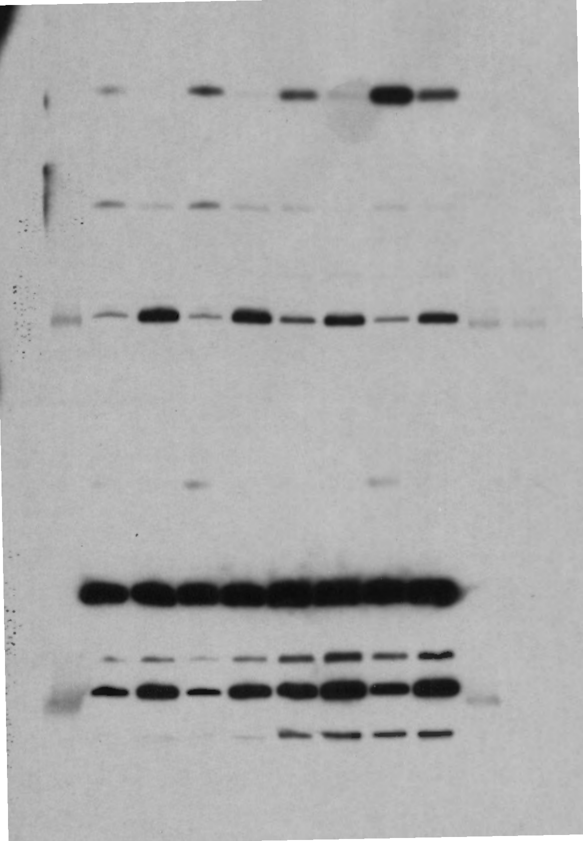

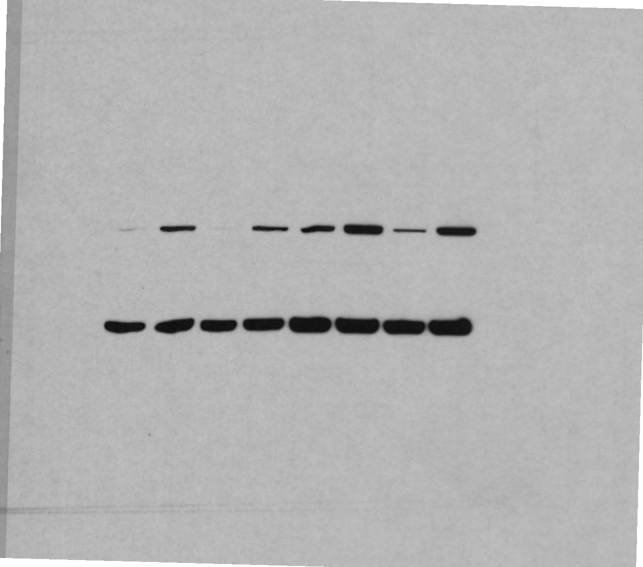

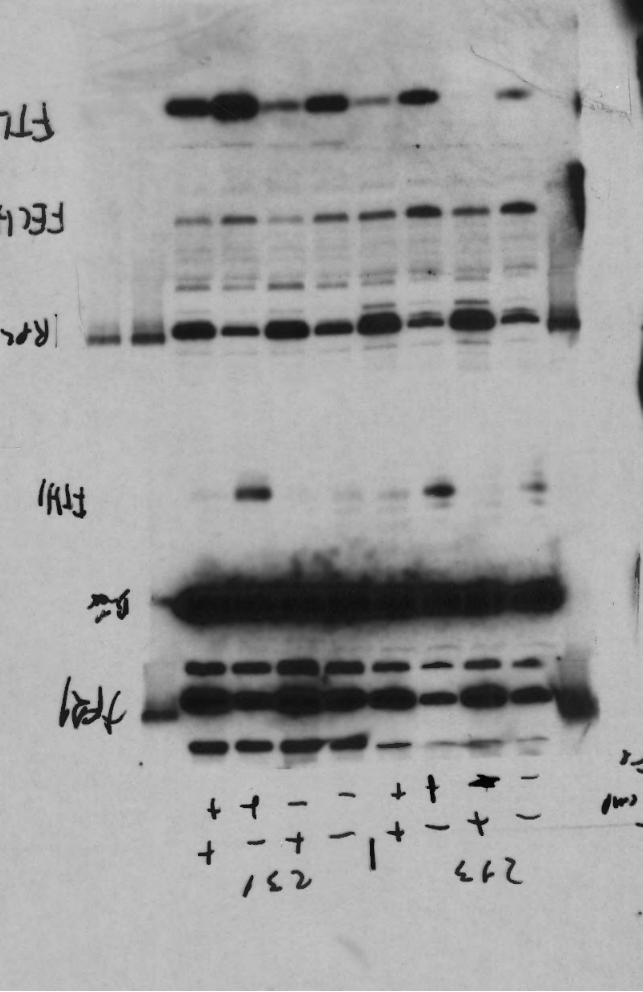

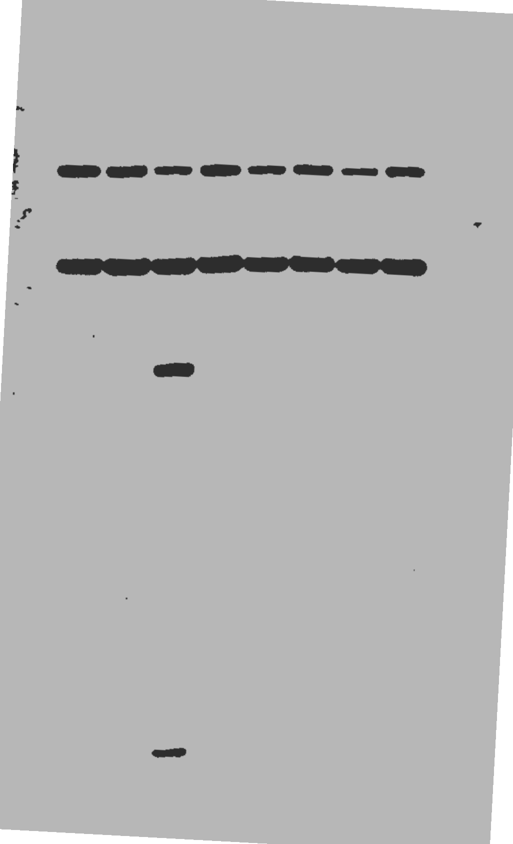

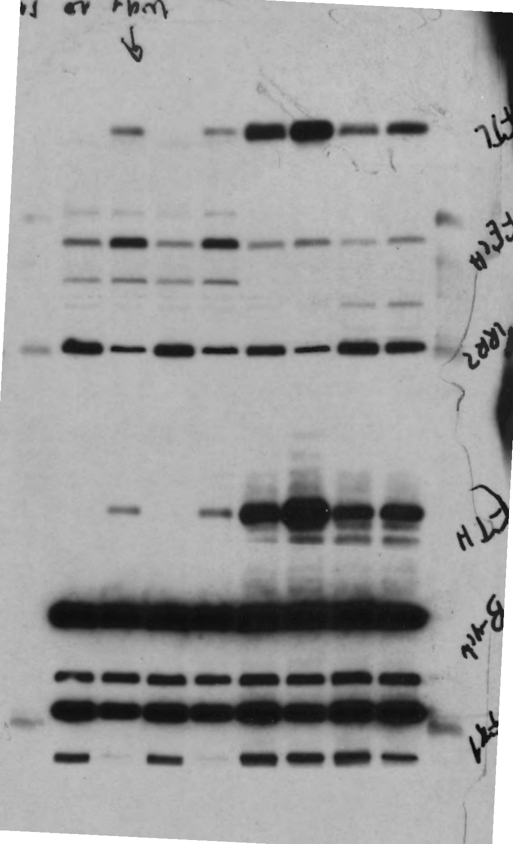

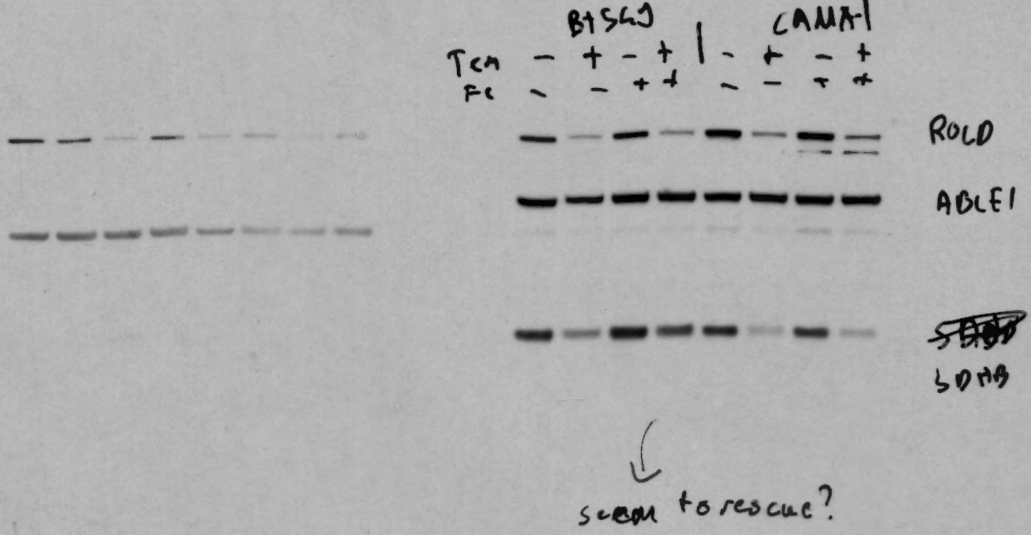
BT549
CAMA-1
Tcn − + − + | − + − +
Fe − − + + | − − + +
ROLD
ABCE1
SDHB
SDHB
seem to rescue?

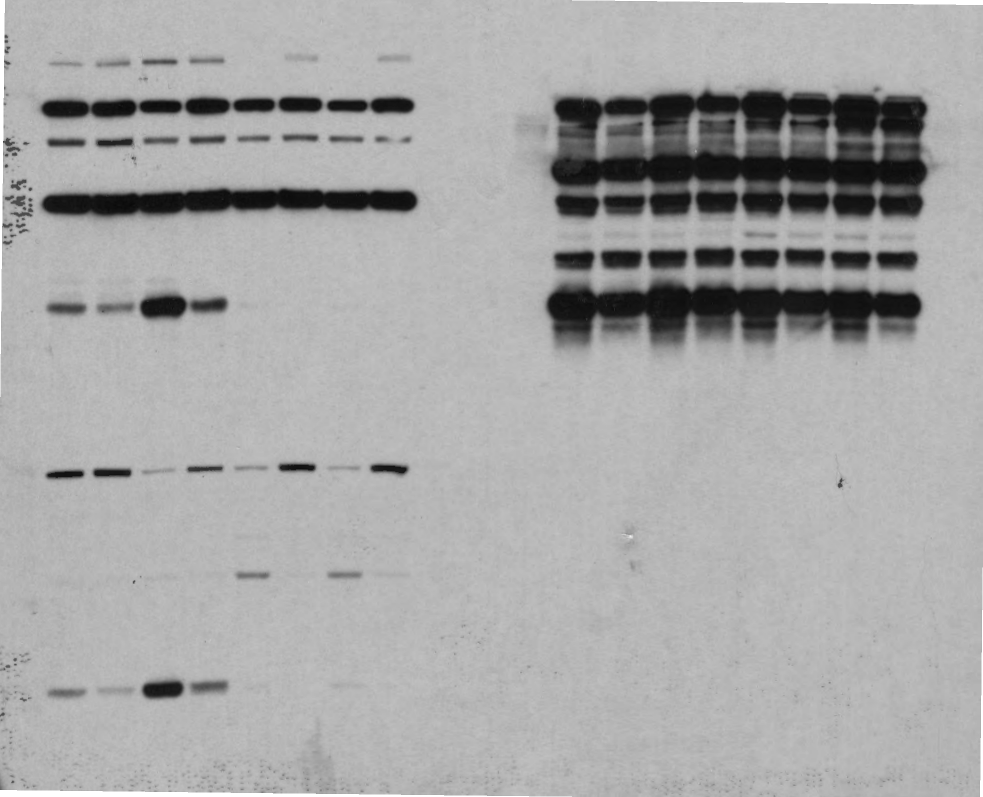

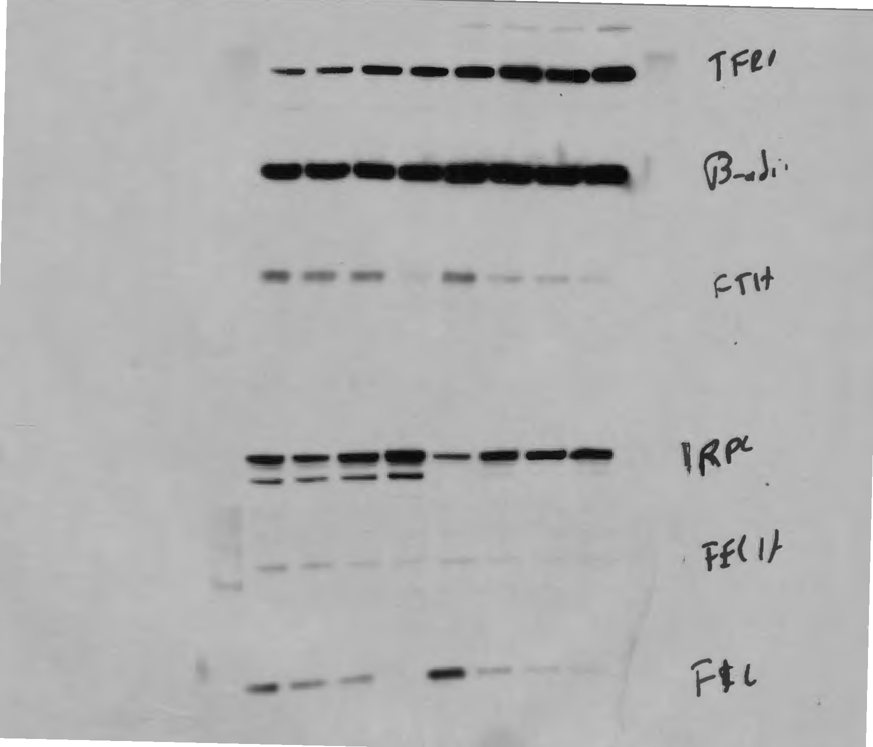

TFR1
FTH

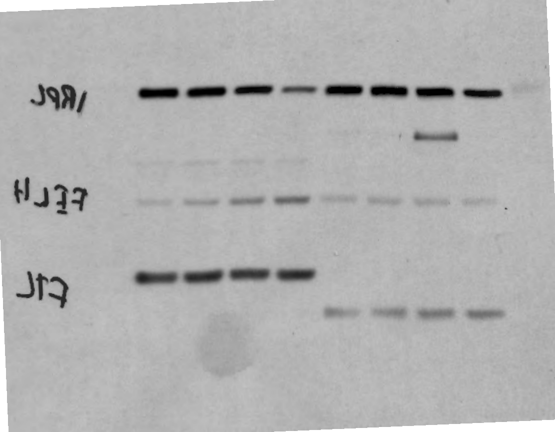

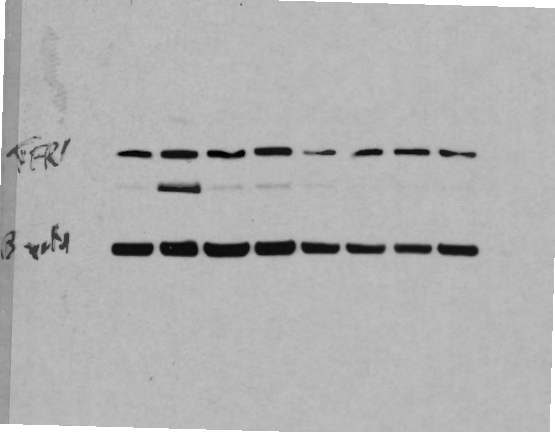
TFR1

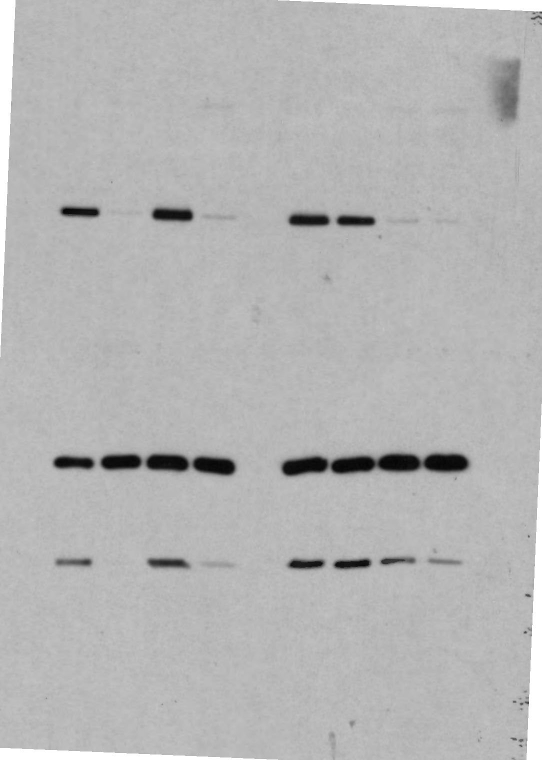

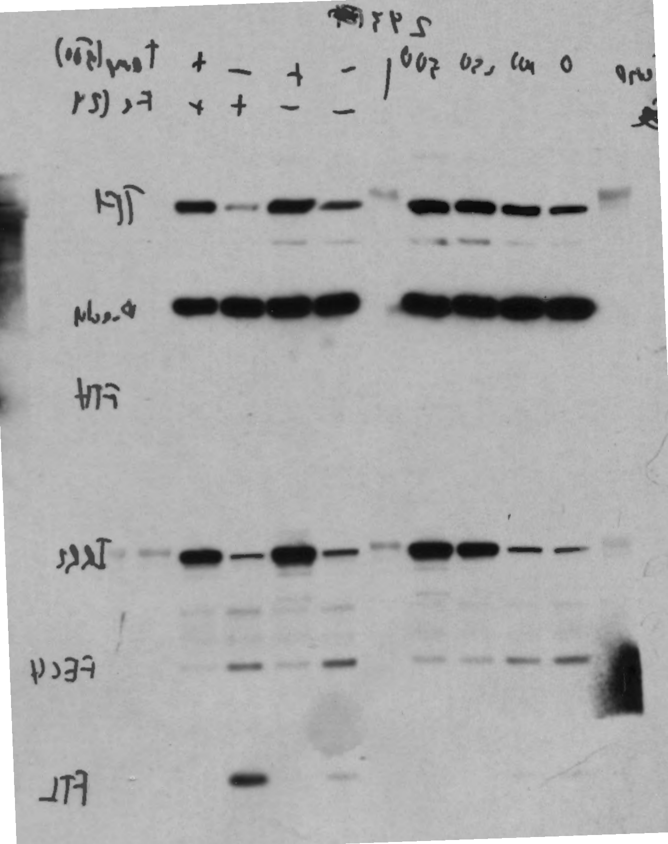

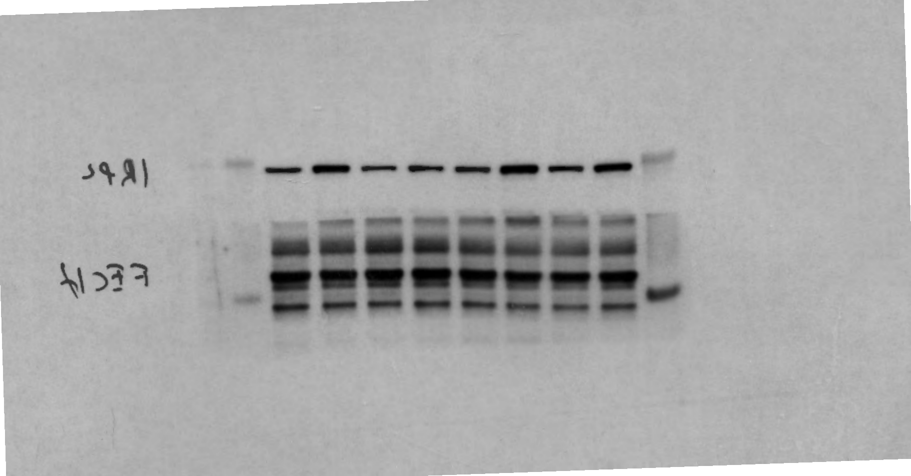

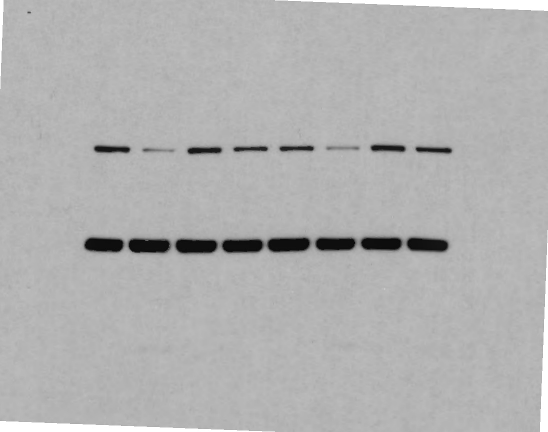

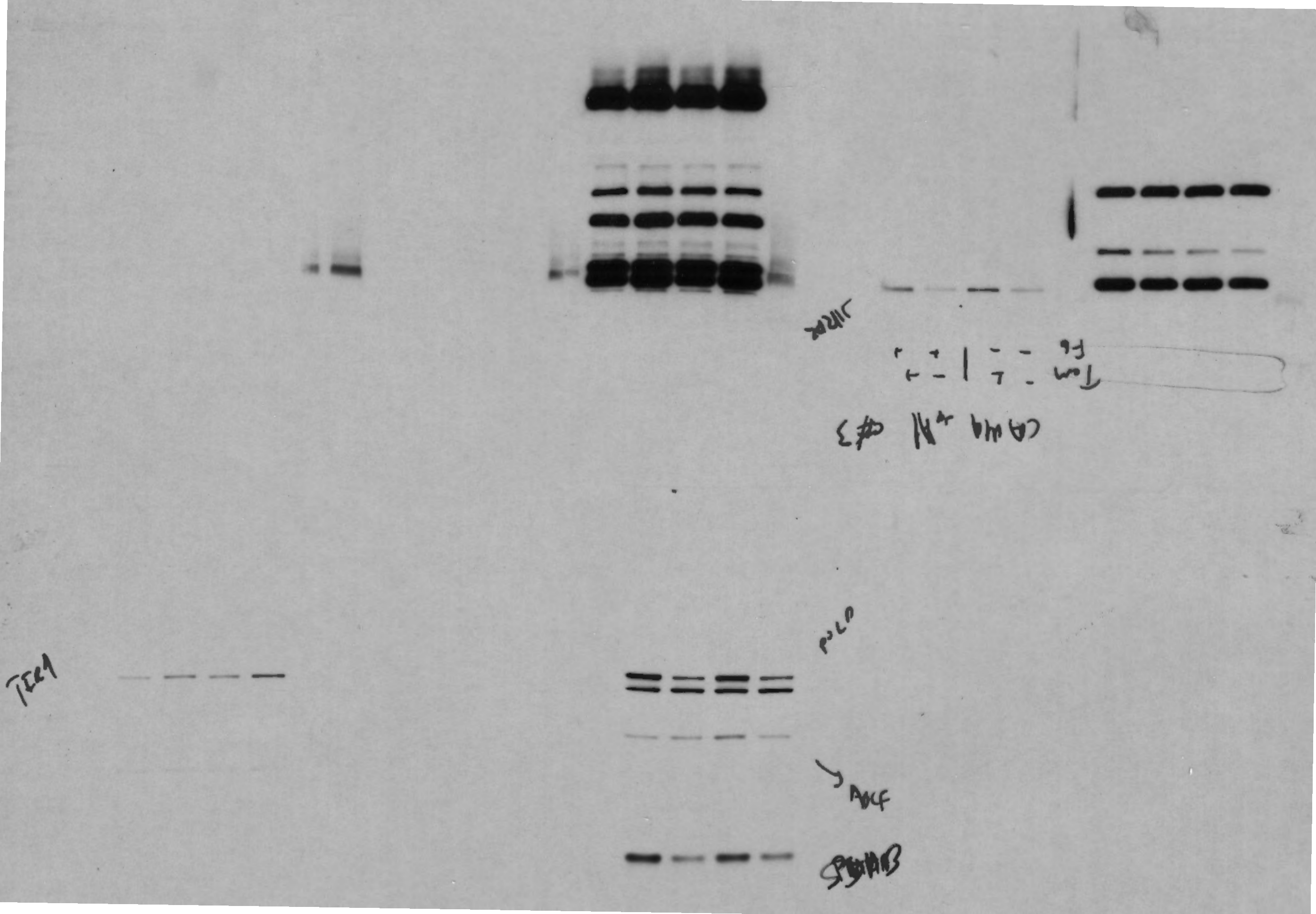

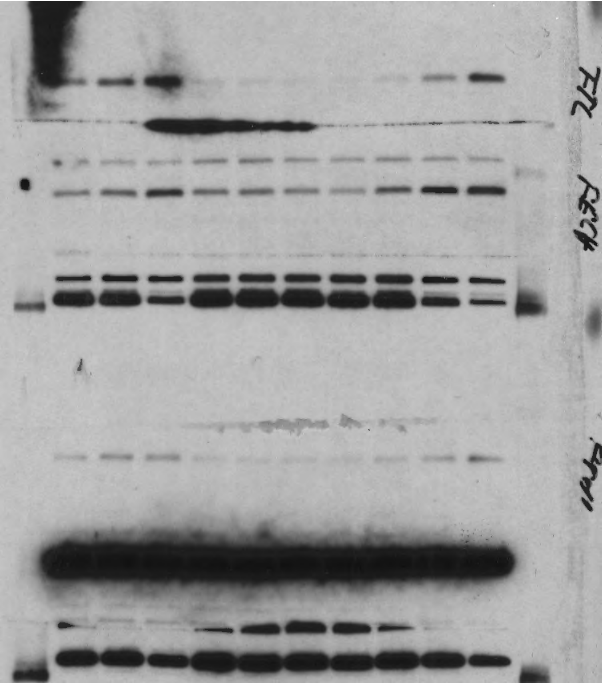

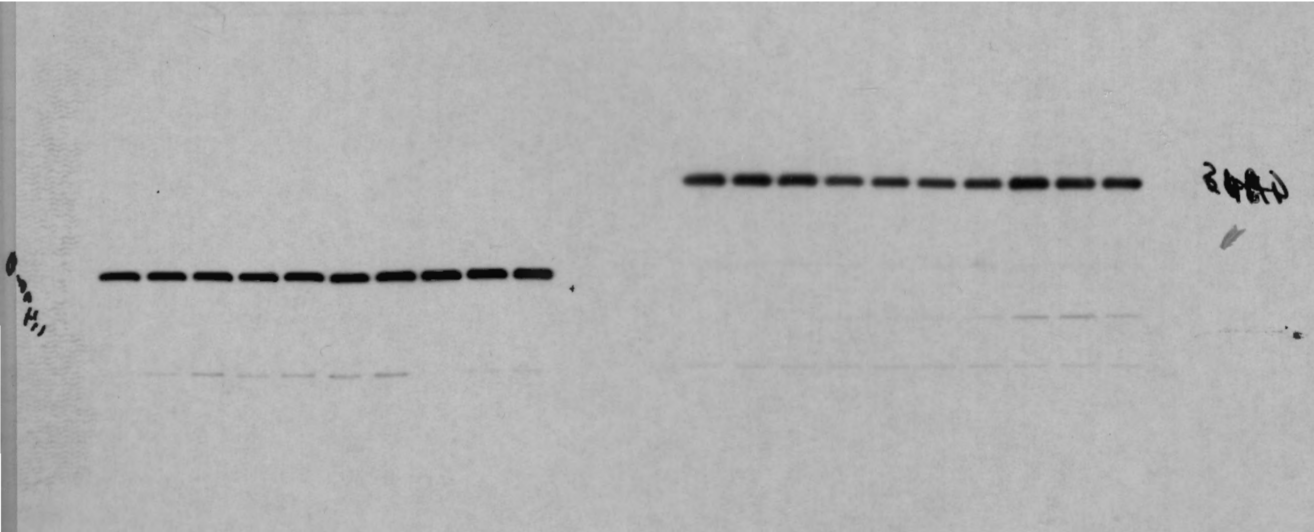

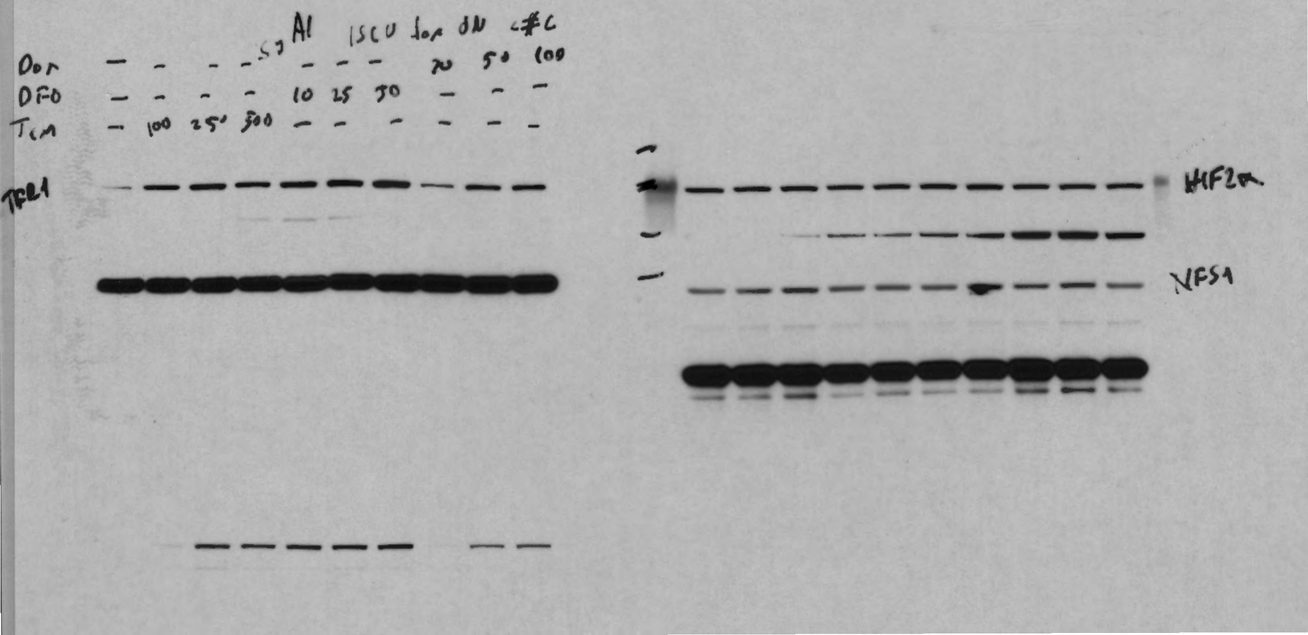

Dor
DFO
TFR1
HIF2α
NFS1

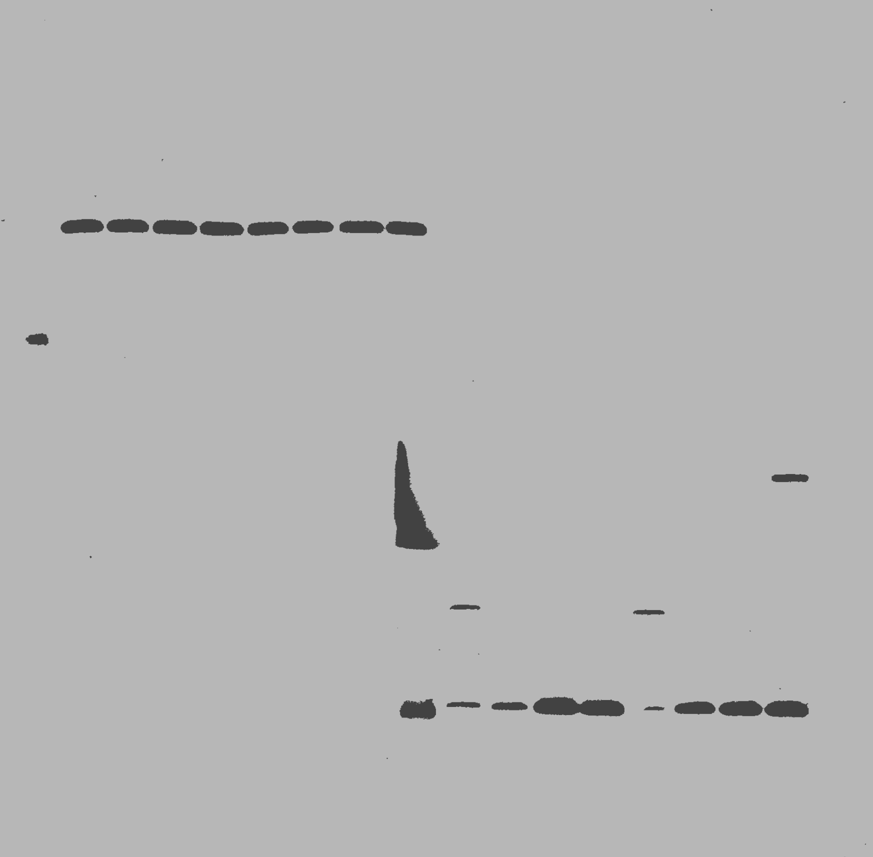

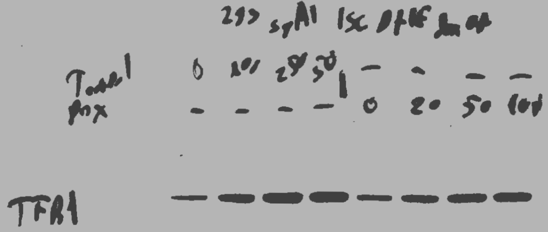
Total
0
20
50
TFR1

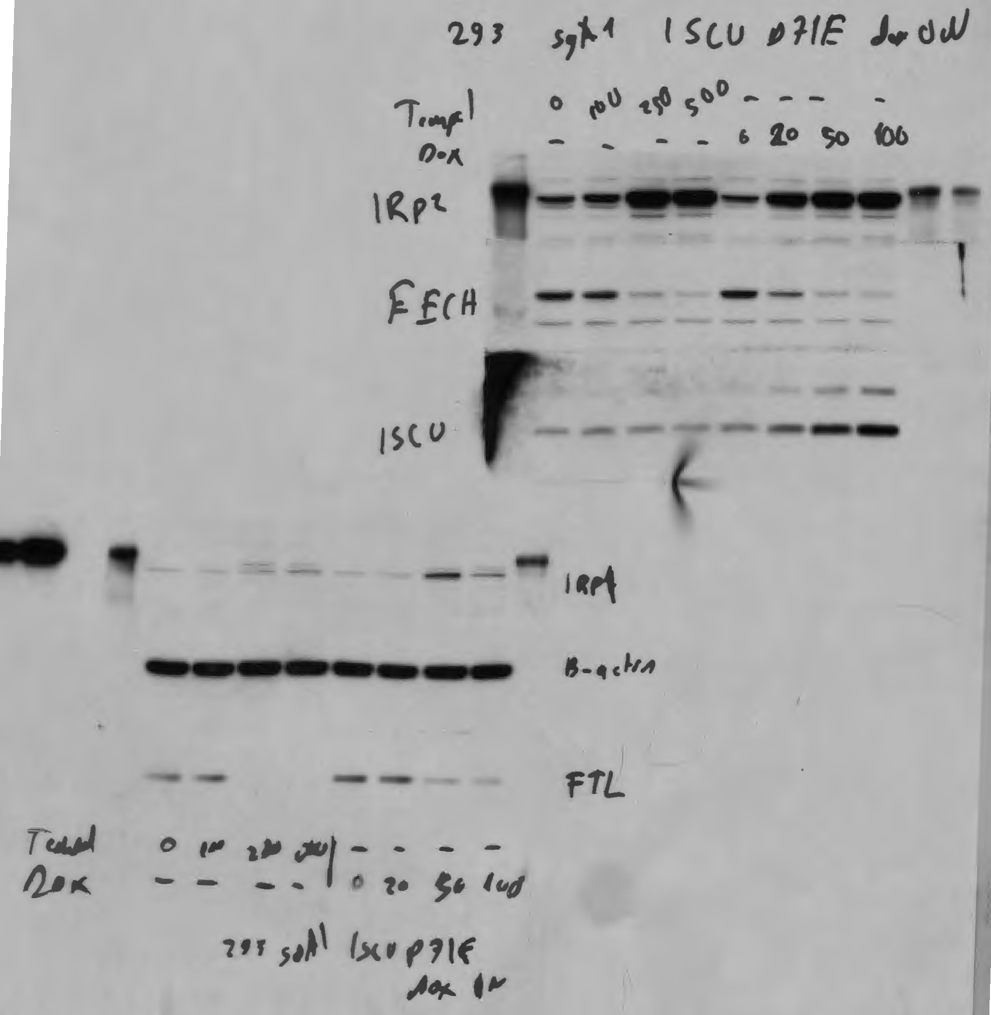
Dox
IRp2
FECH
ISCU
FTL

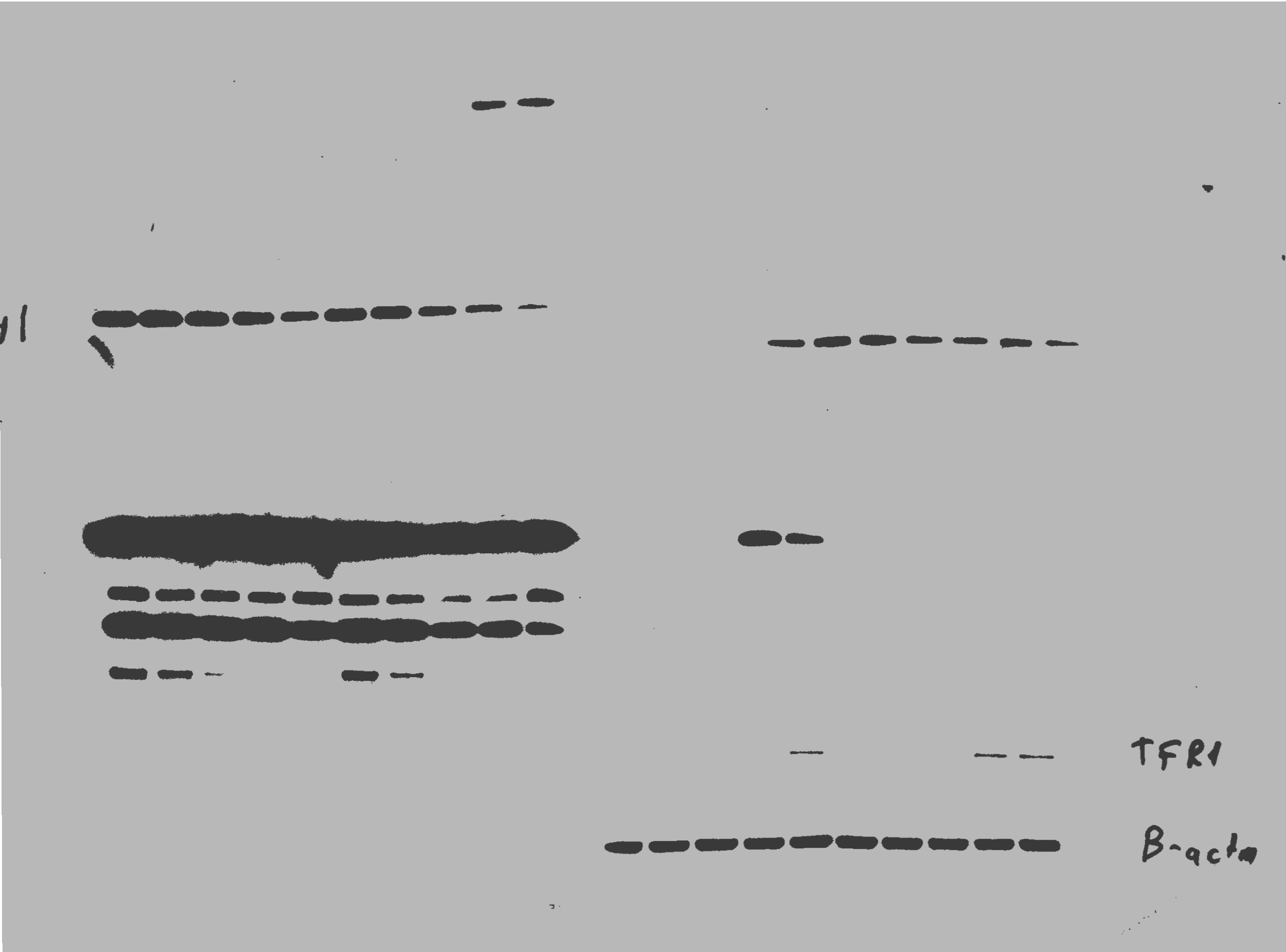
TFR1
B-actin

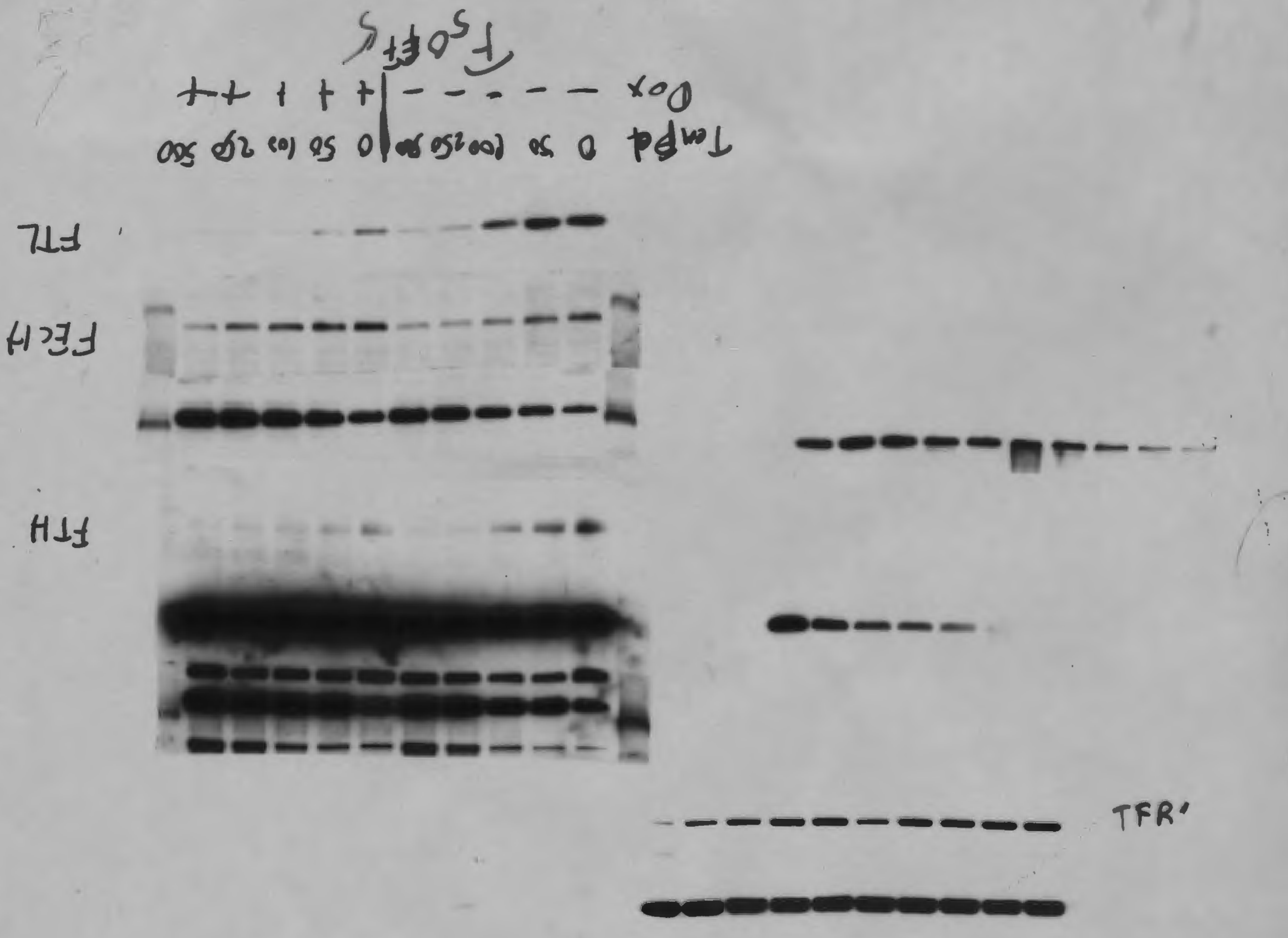

FTL
FECH
FTH
Dox
TFR'

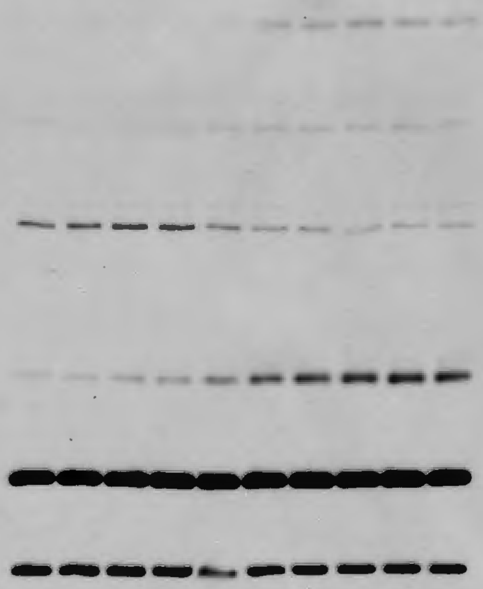

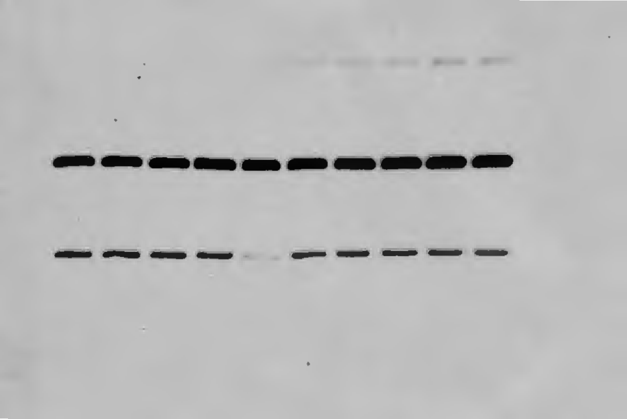

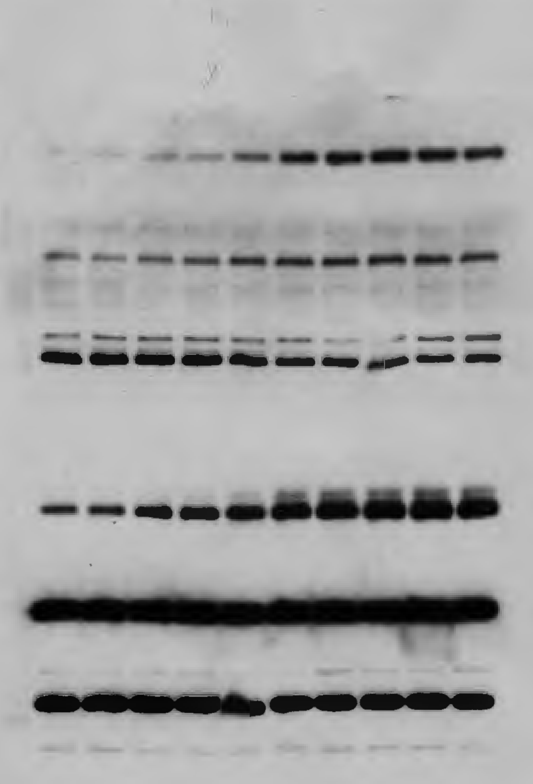

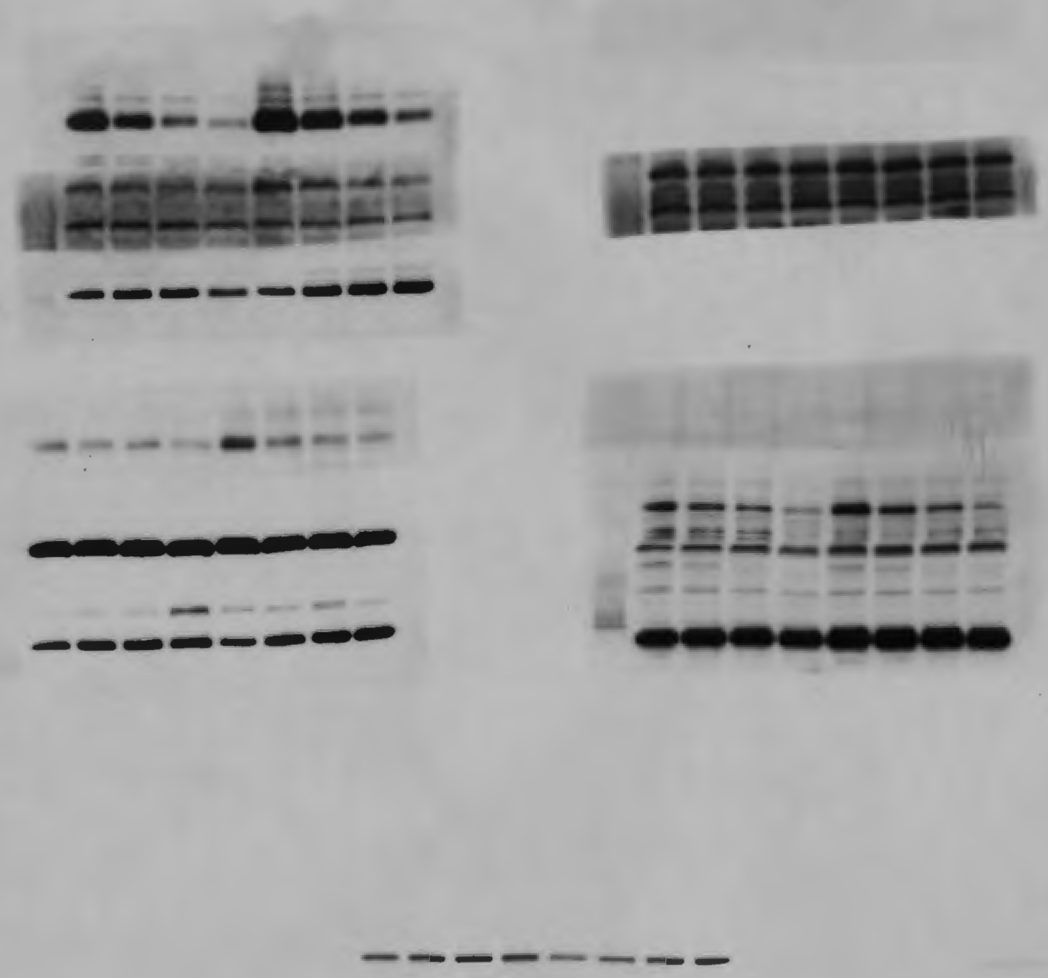

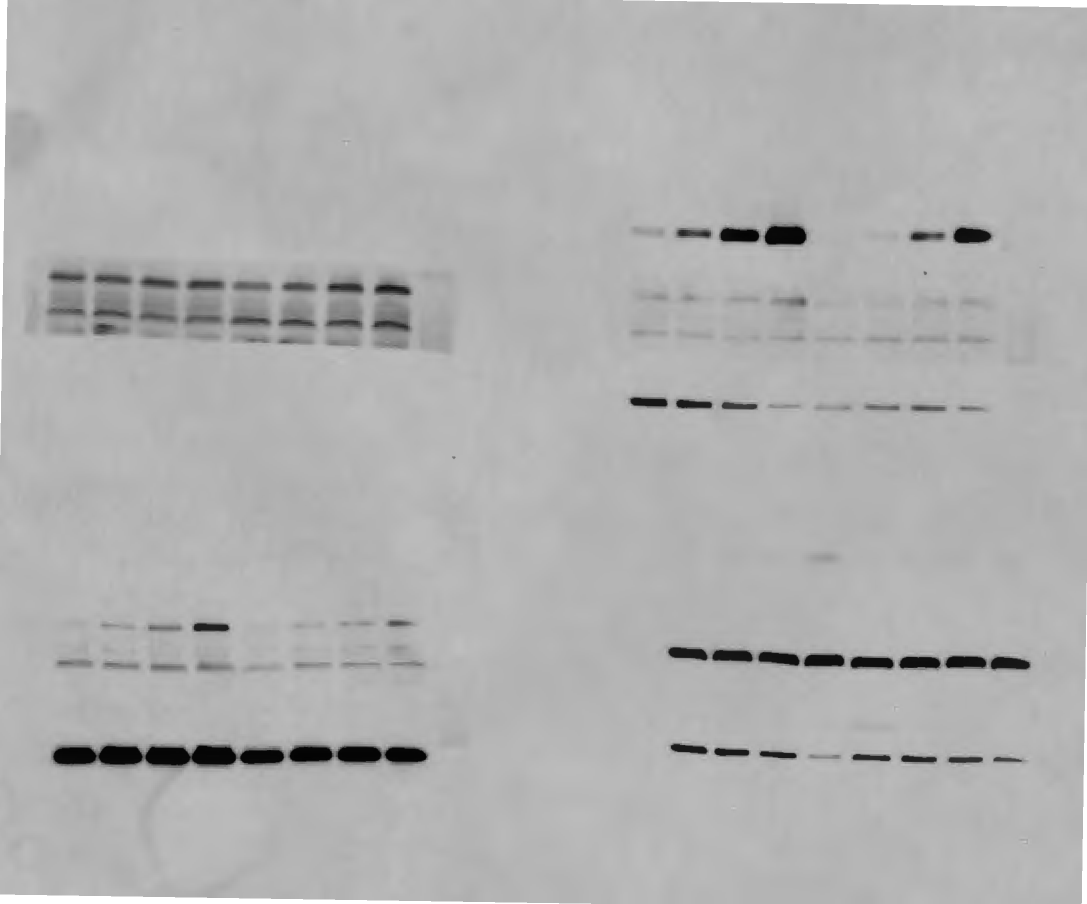

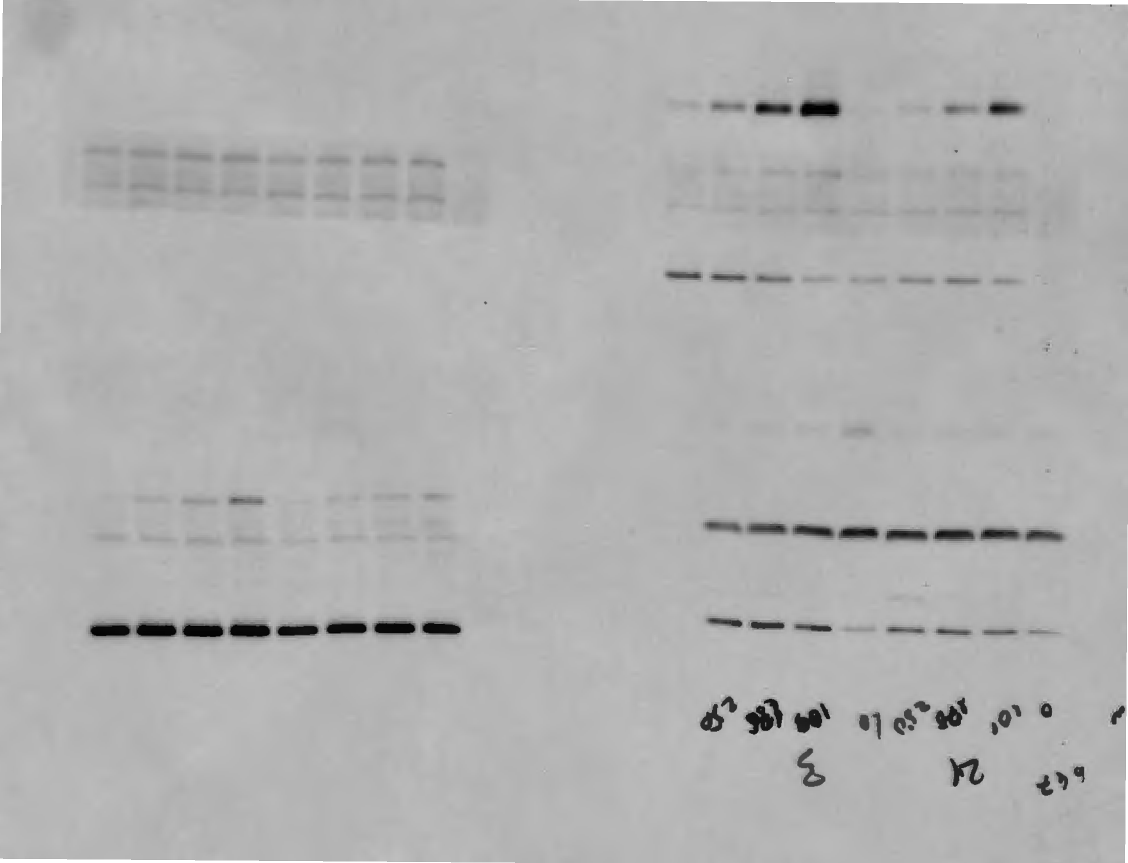

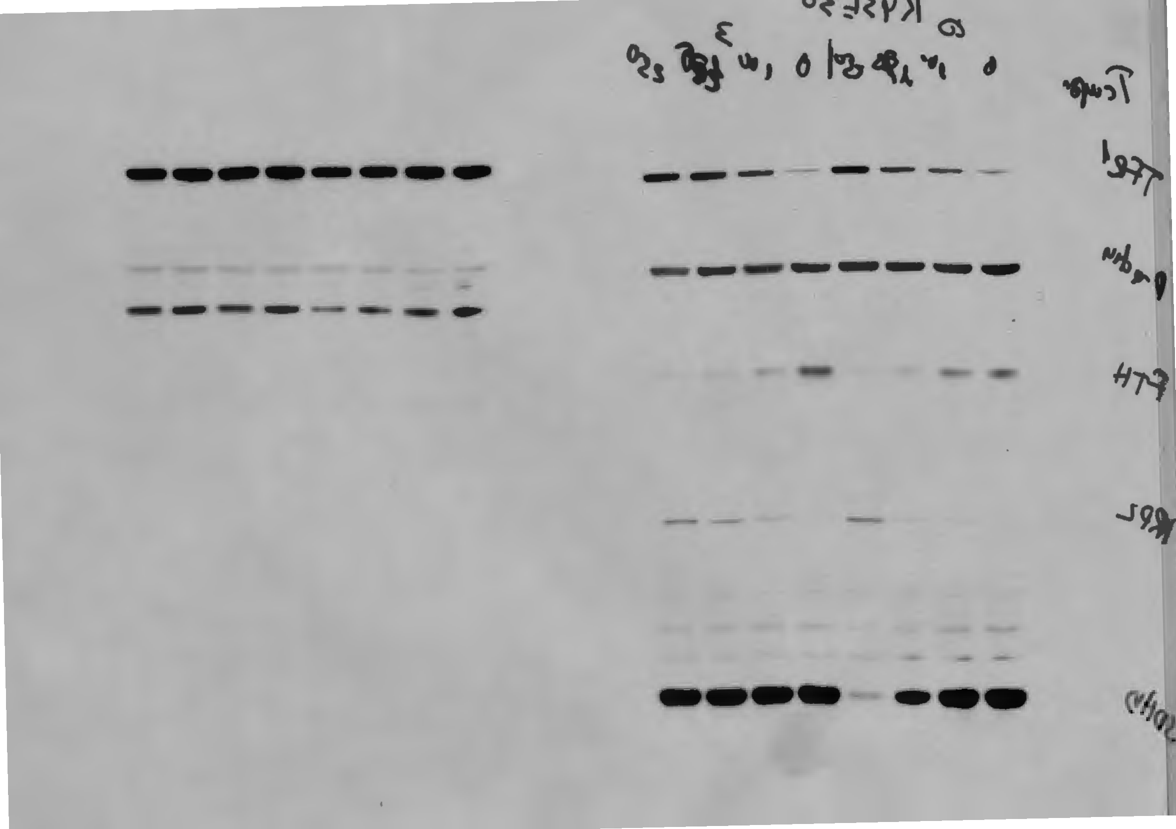

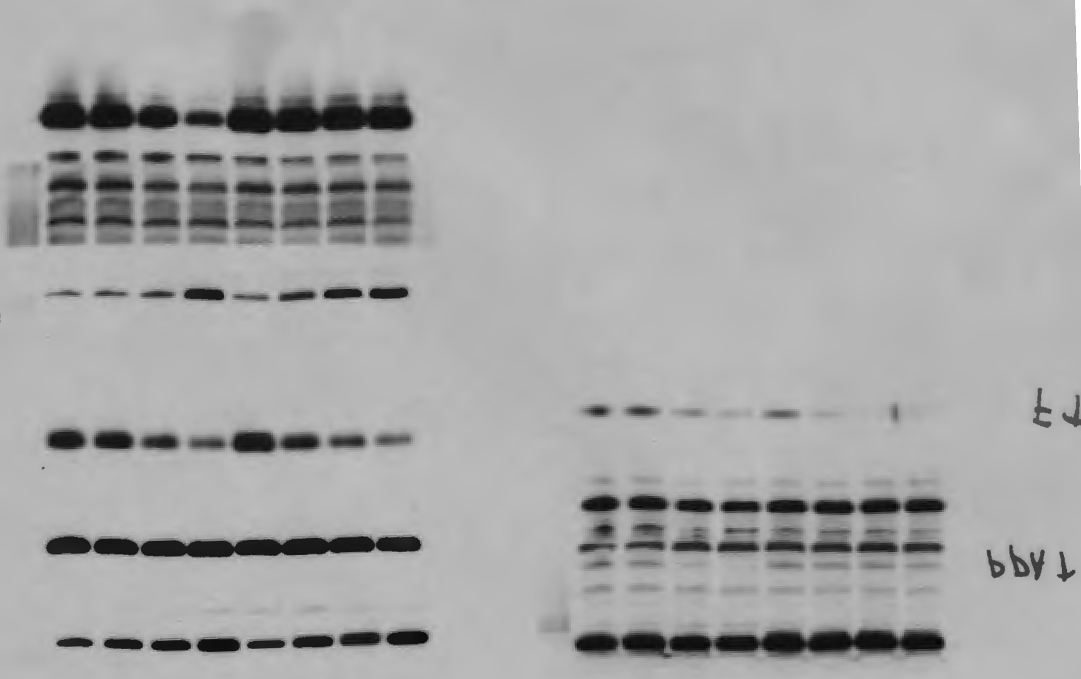

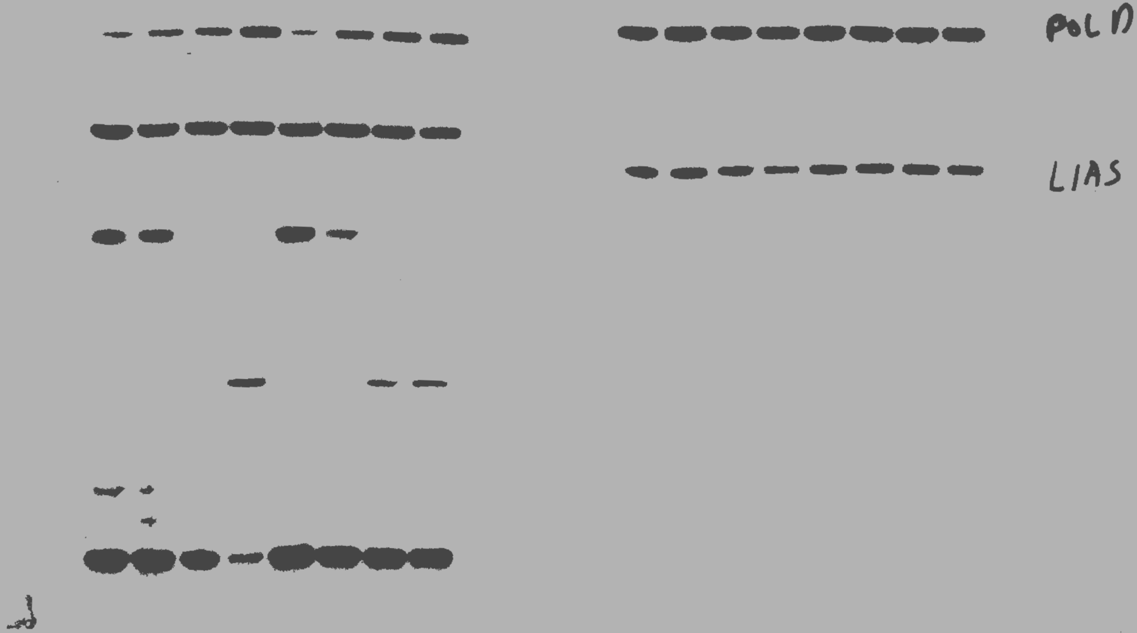

PoLD
LIAS

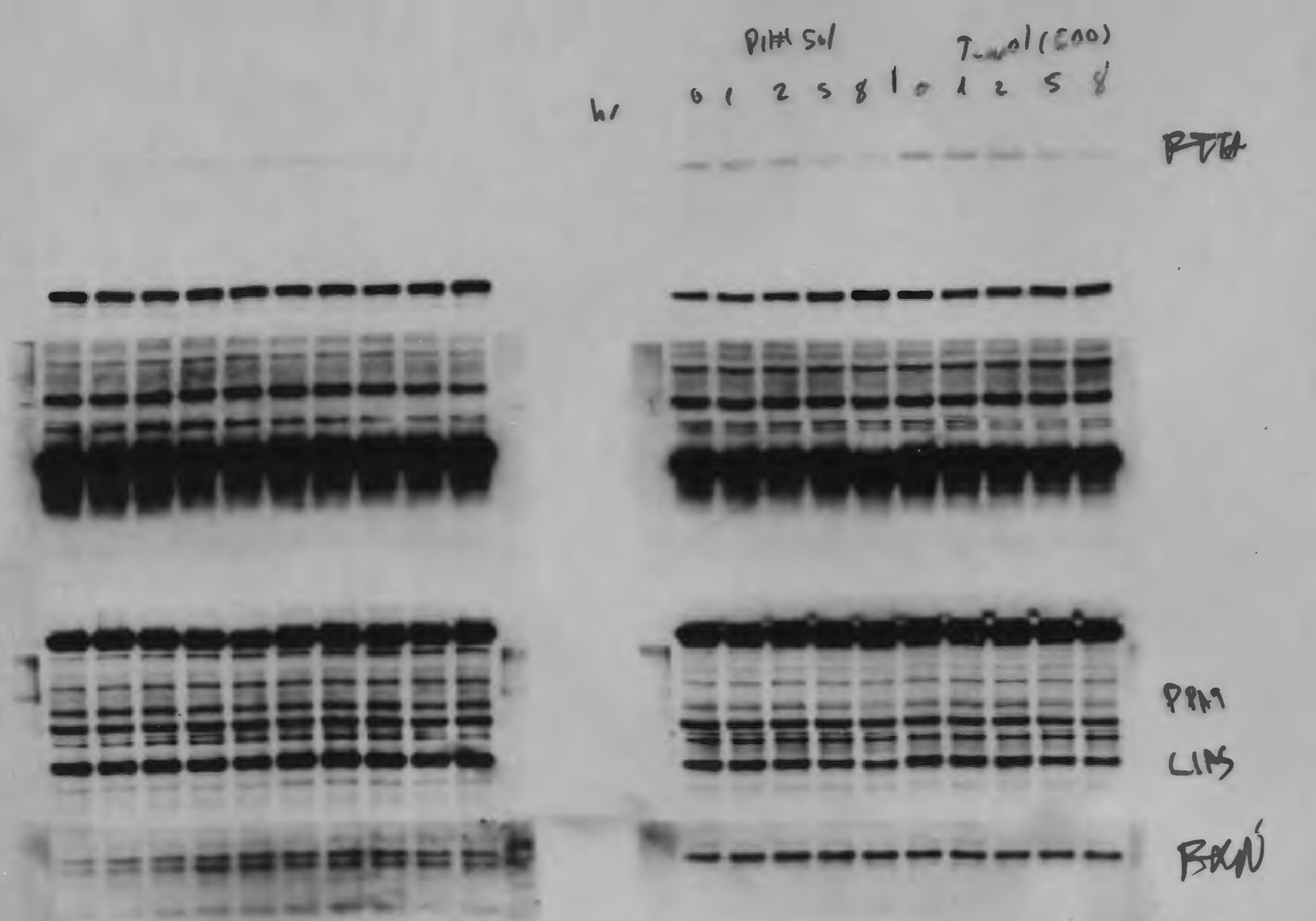
hr
0 1 2 5 8
0 1 2 5 8
(500)

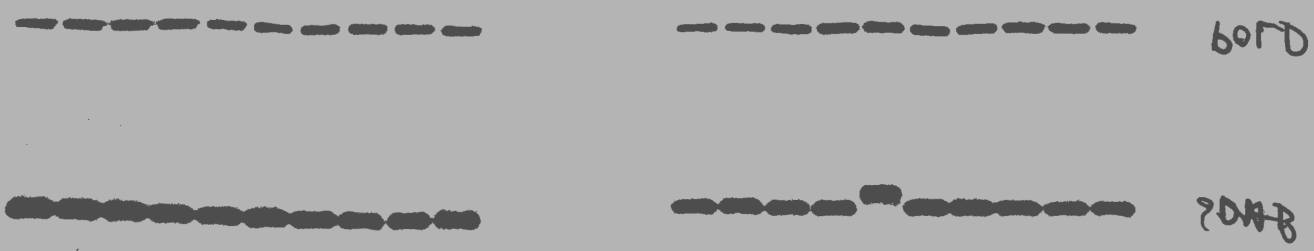

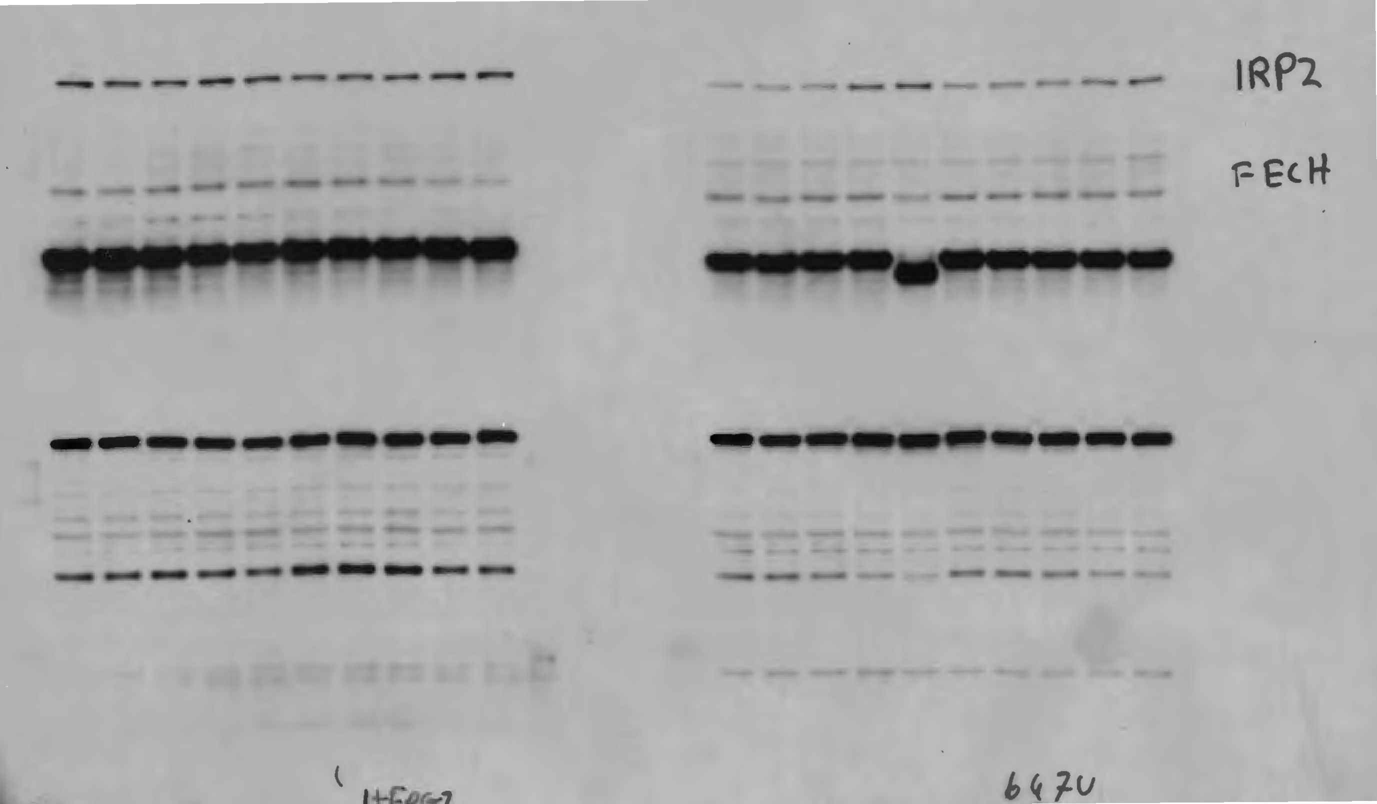
IRP2
FECH
647U

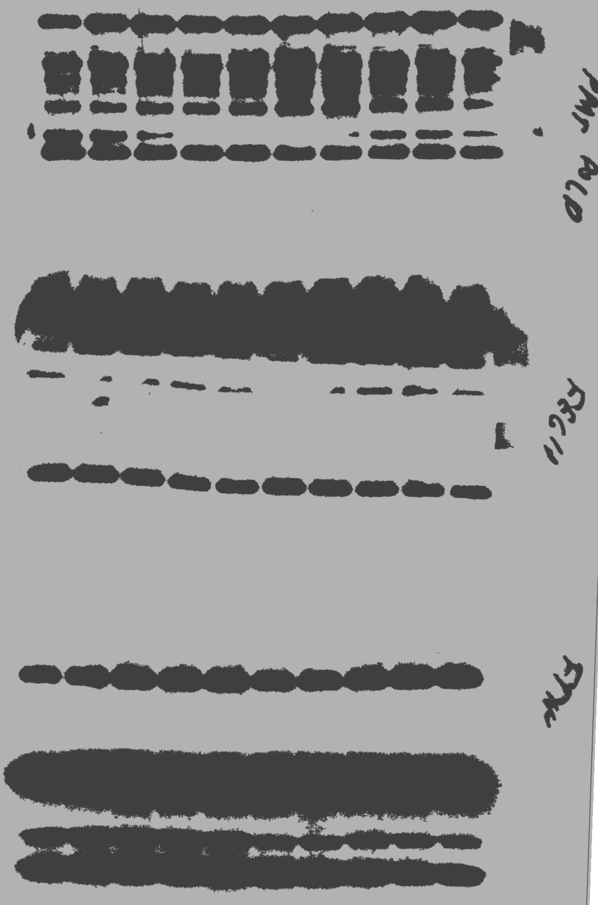

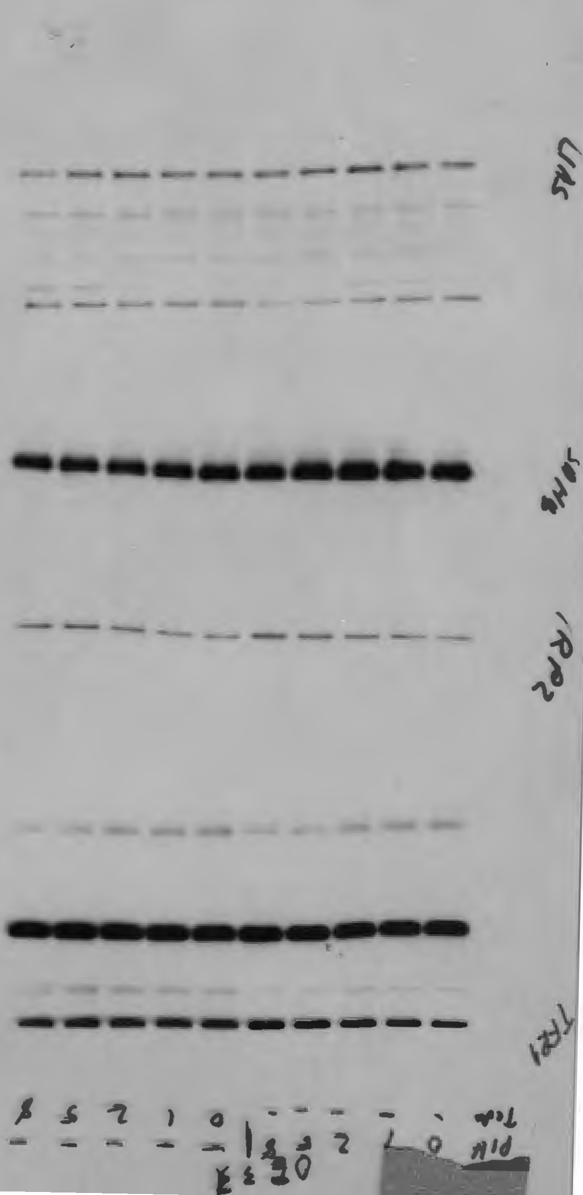

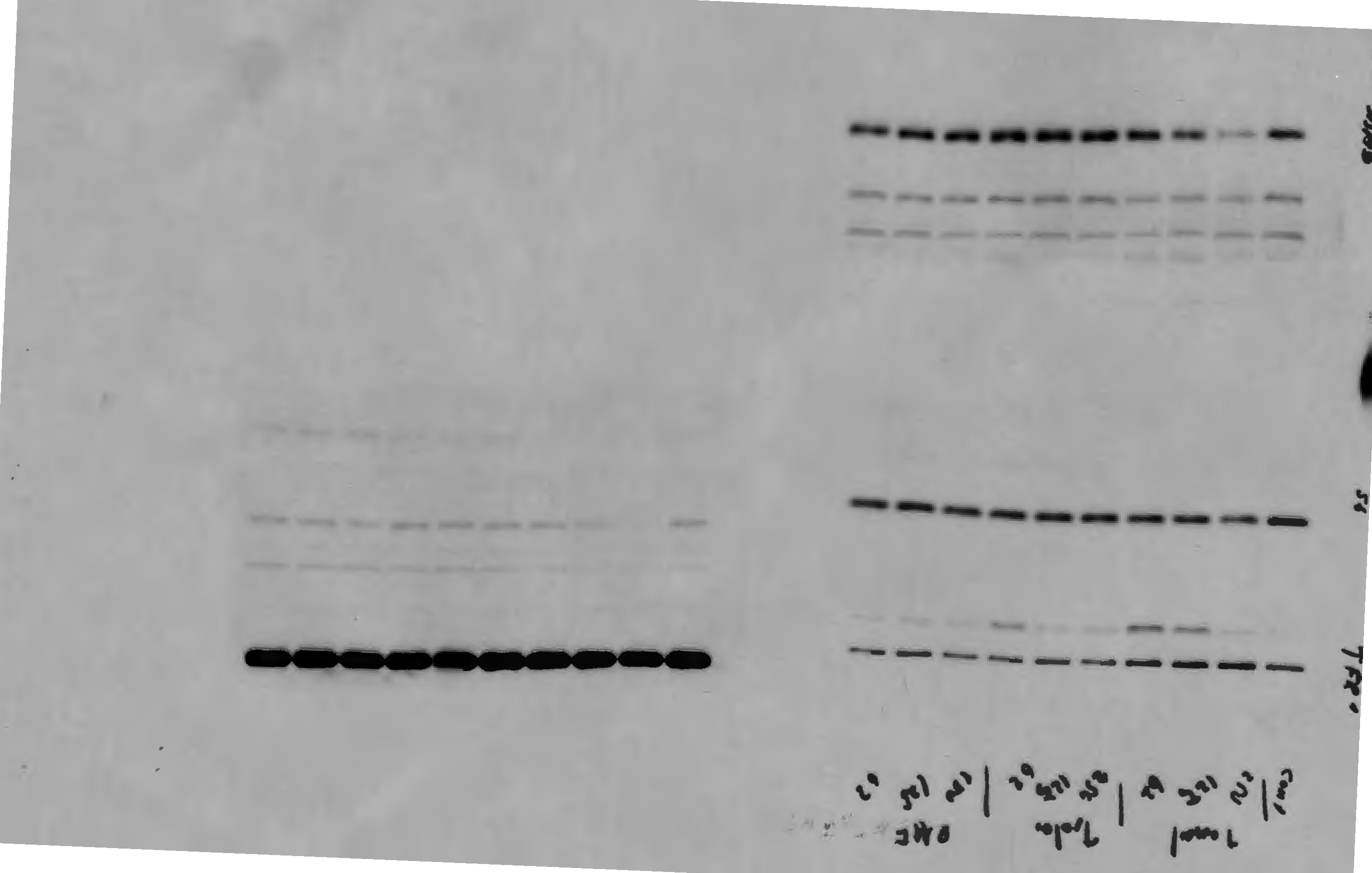

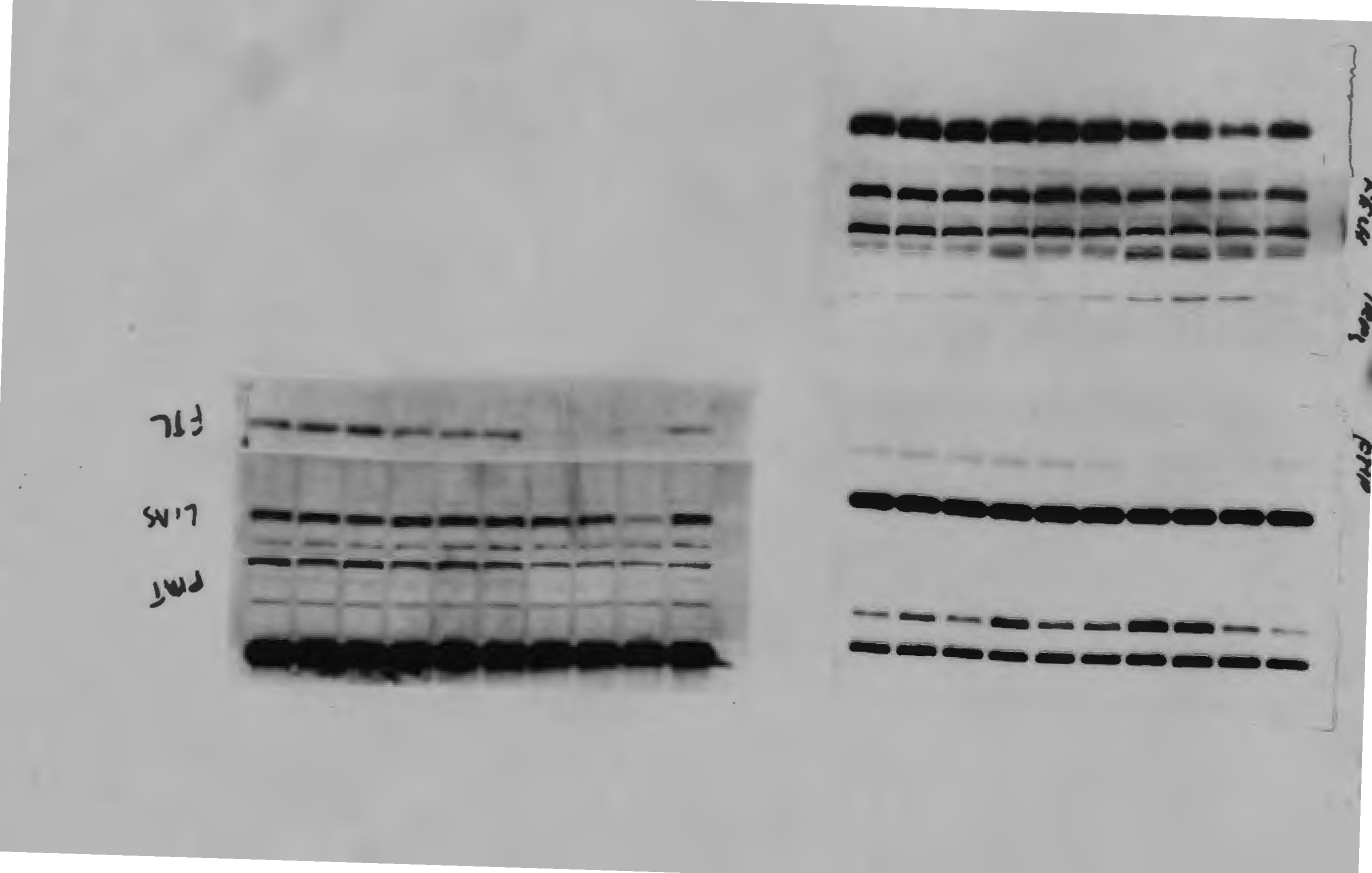

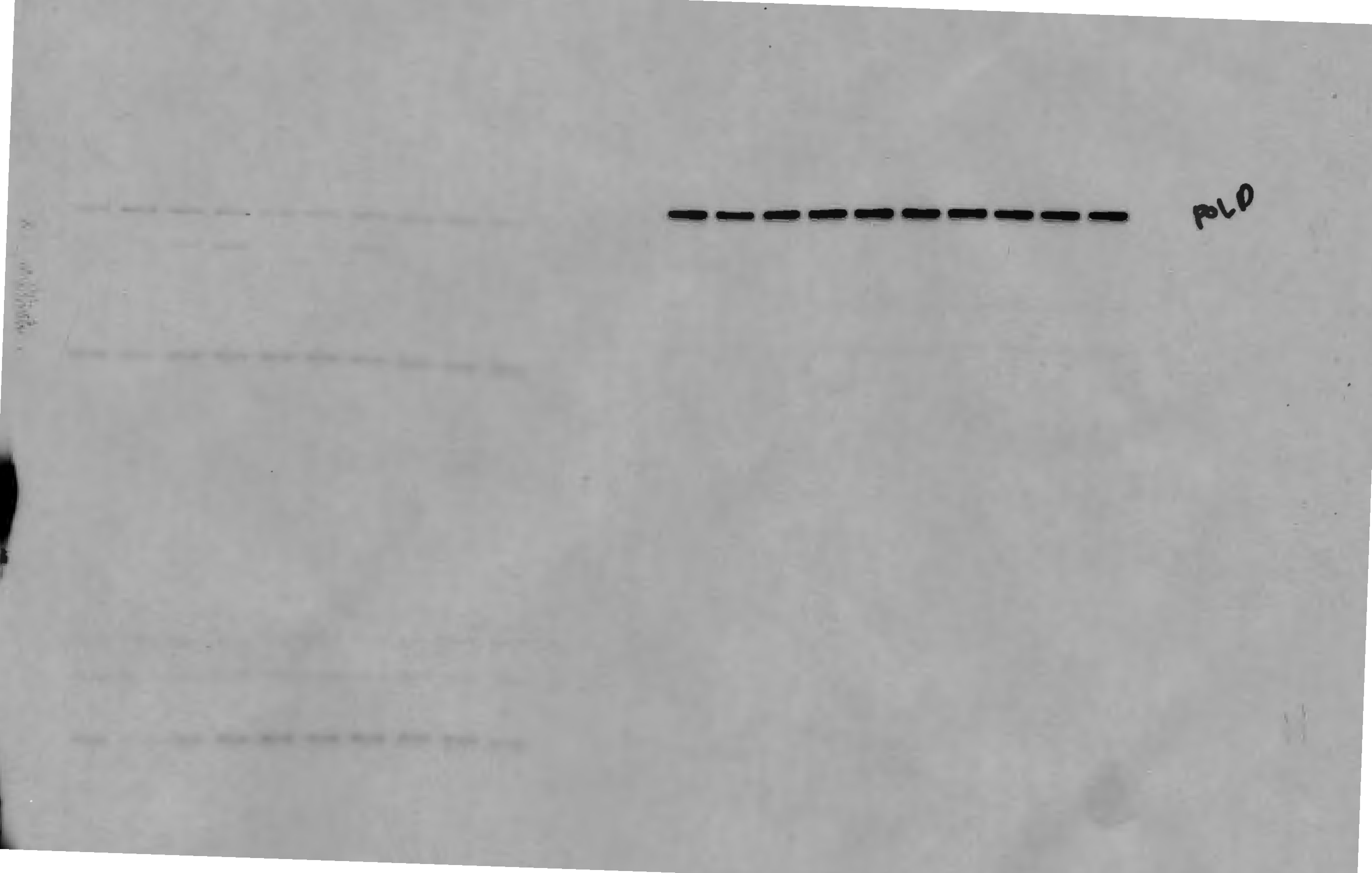
POLD

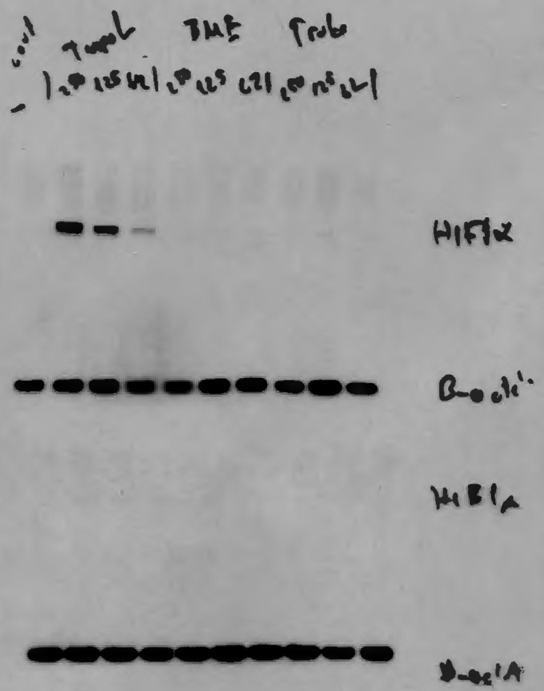

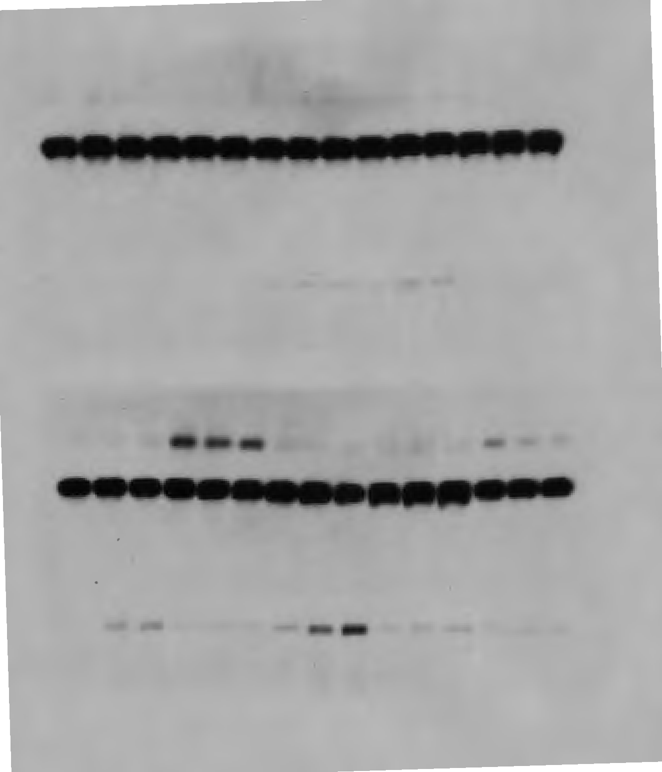

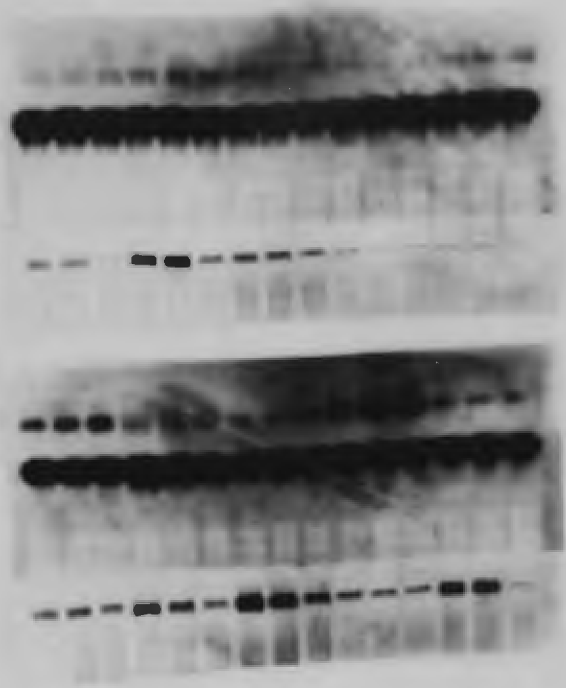

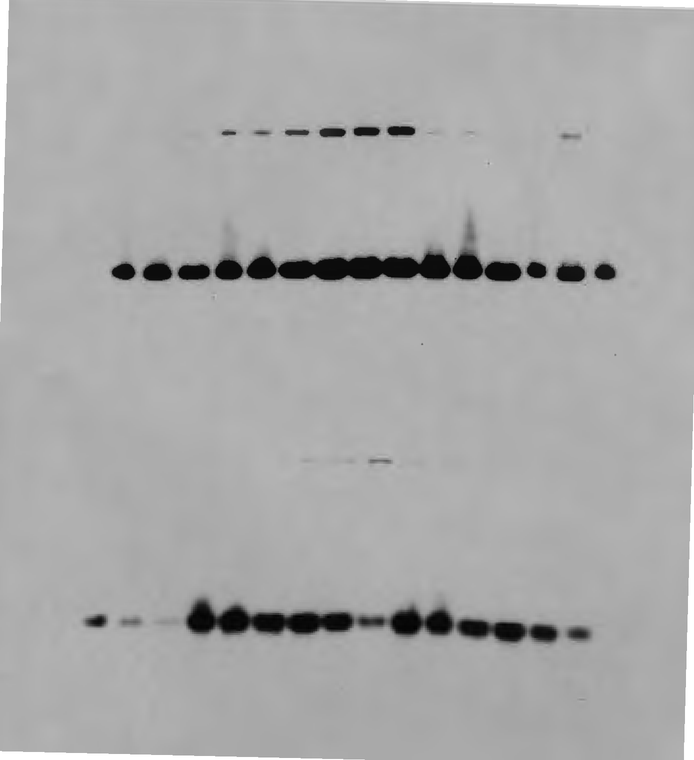

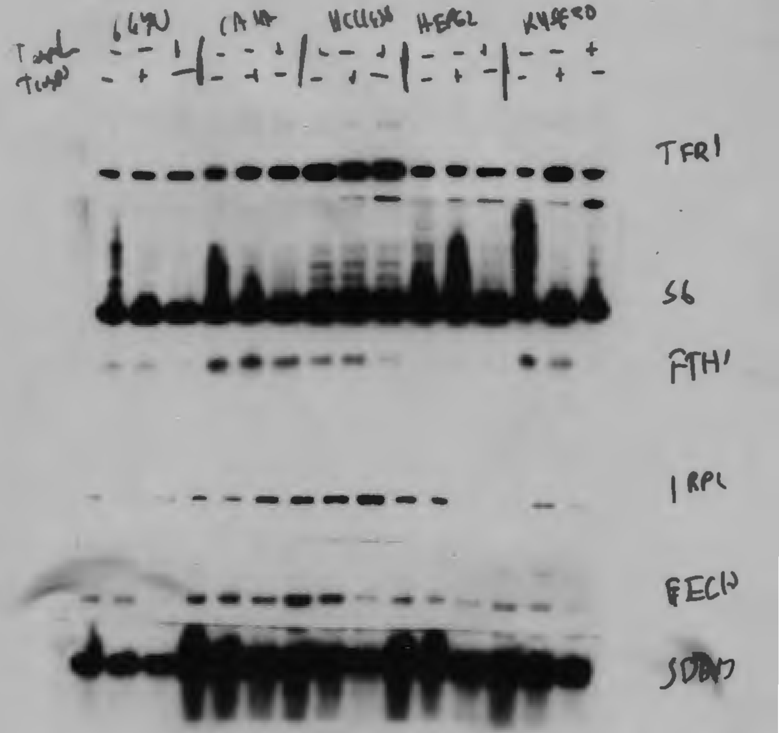
HEPG2
TFR1
S6
FTH1
FECH

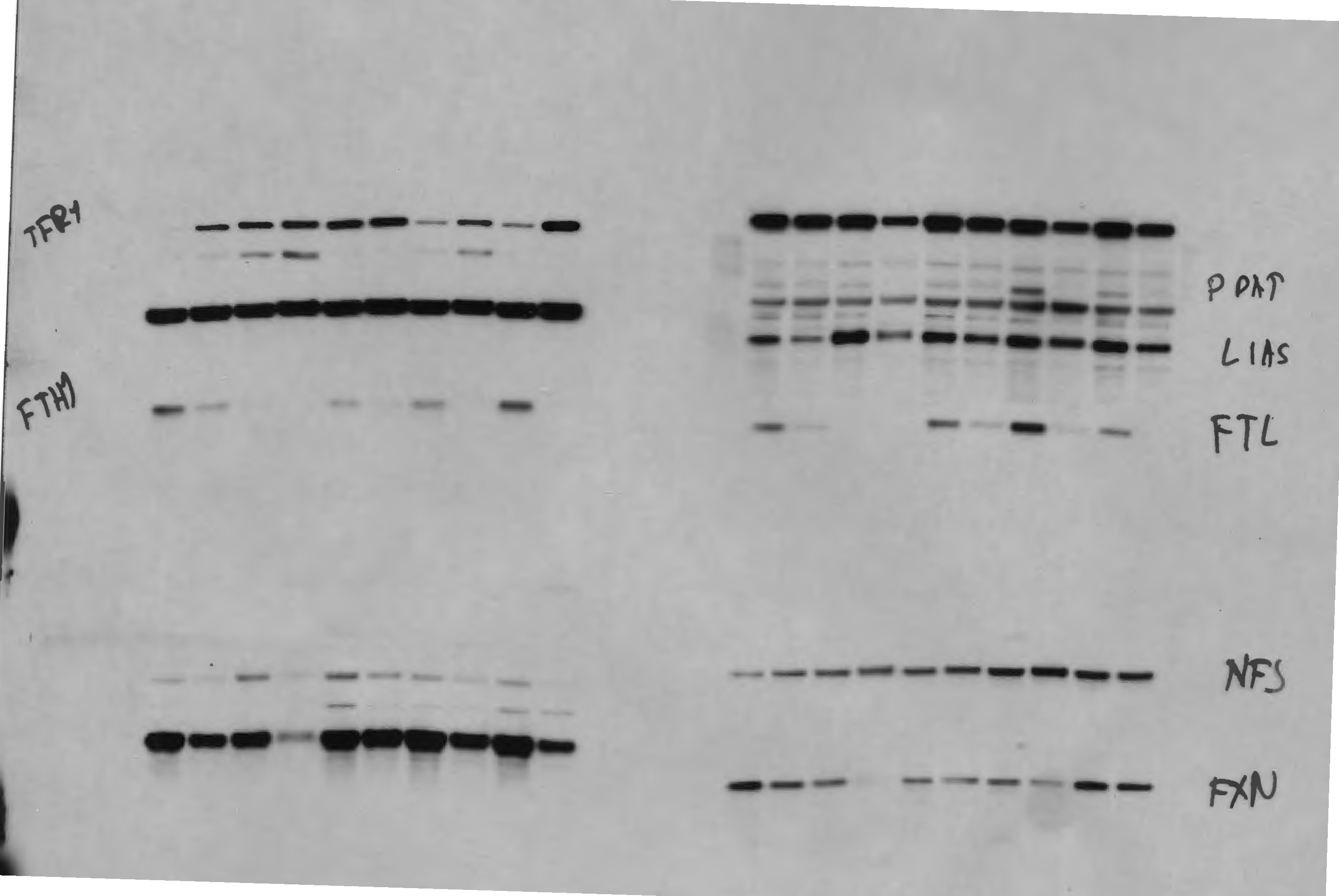
TFR1
FTH1
P DAT
LIAS
FTL
NFS
FXN

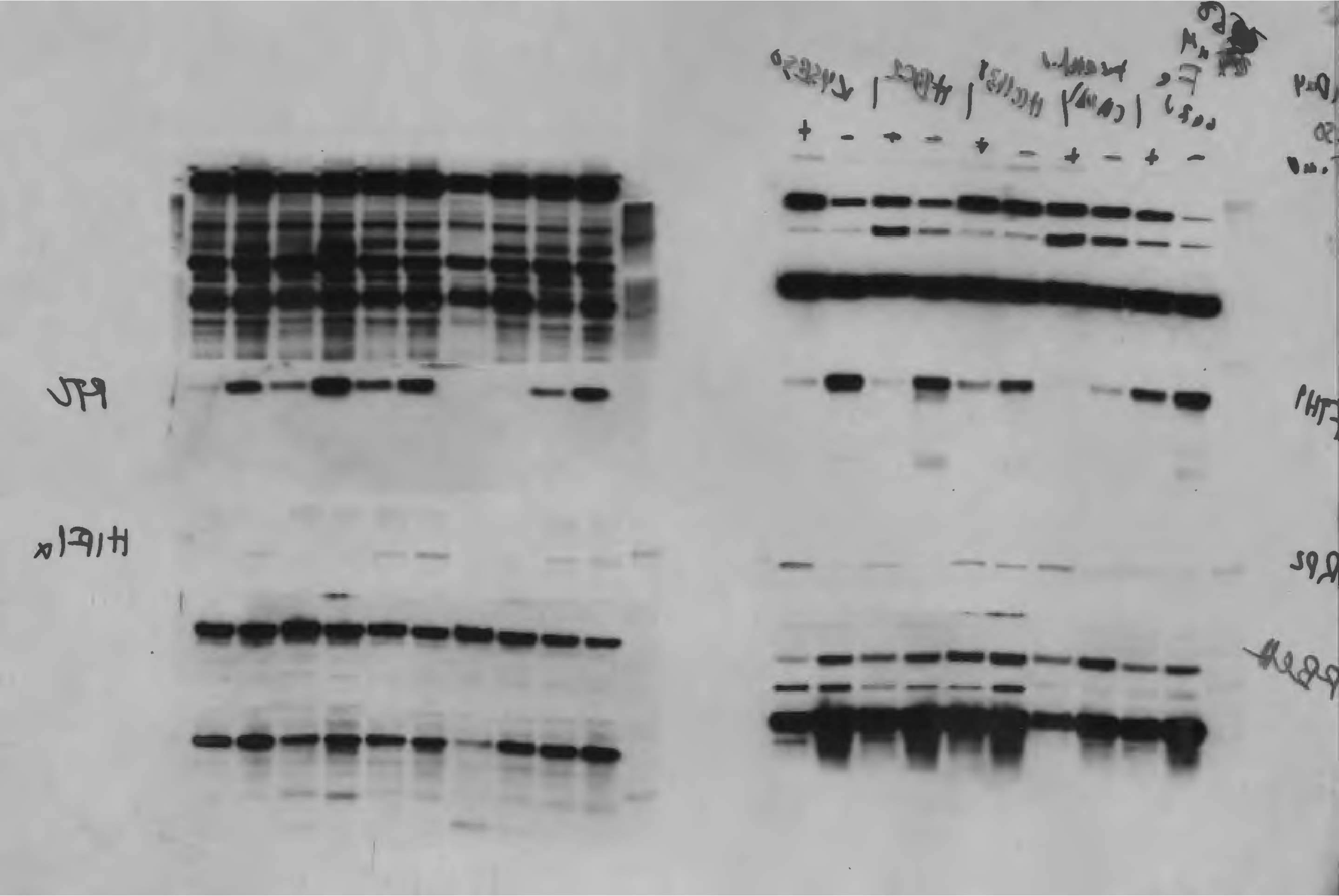

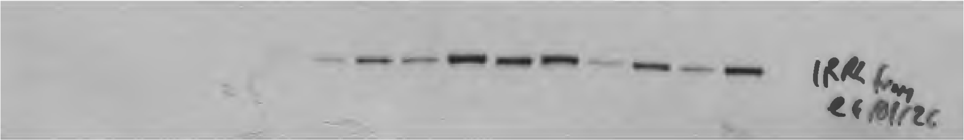
IRR

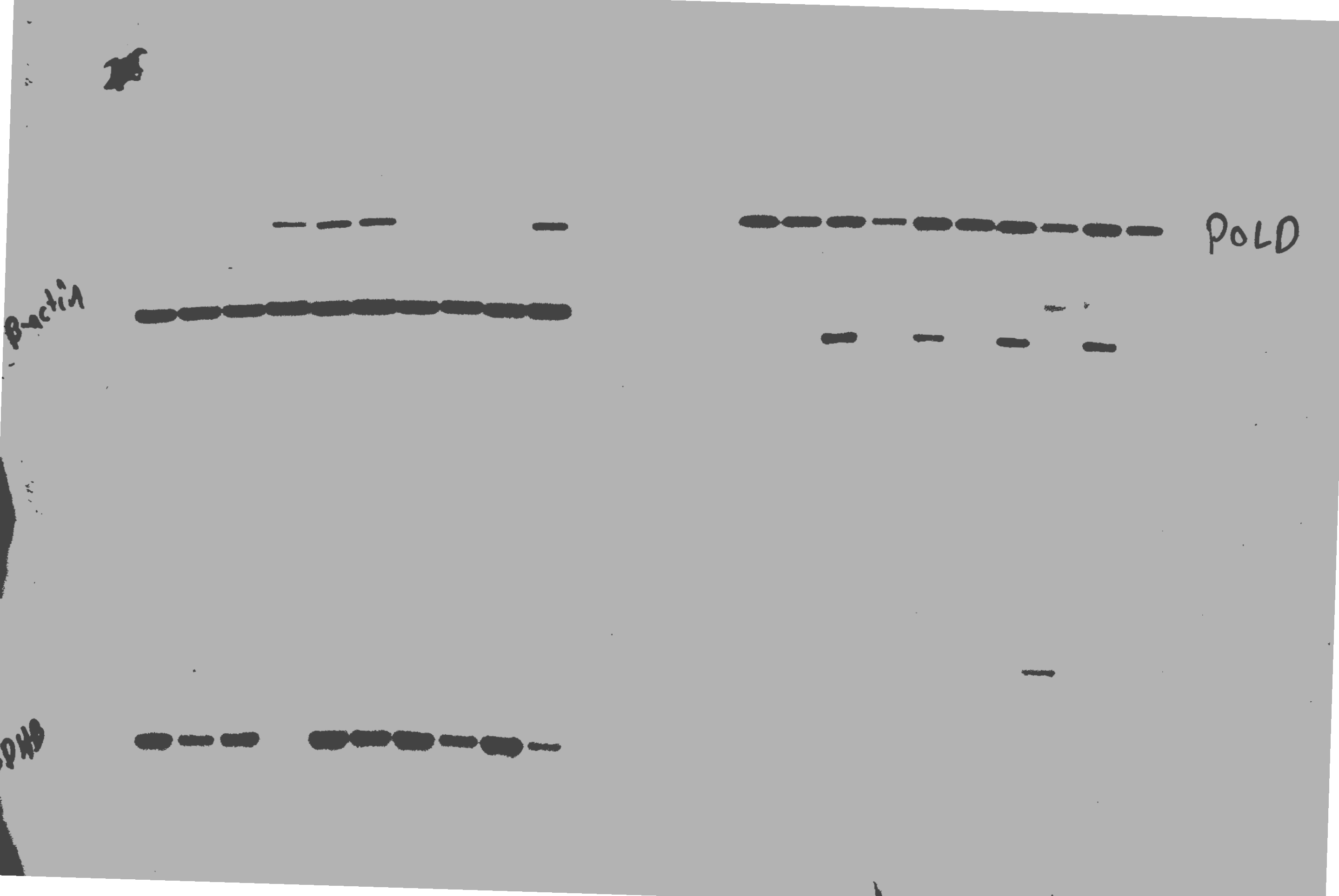
POLD
β-actin

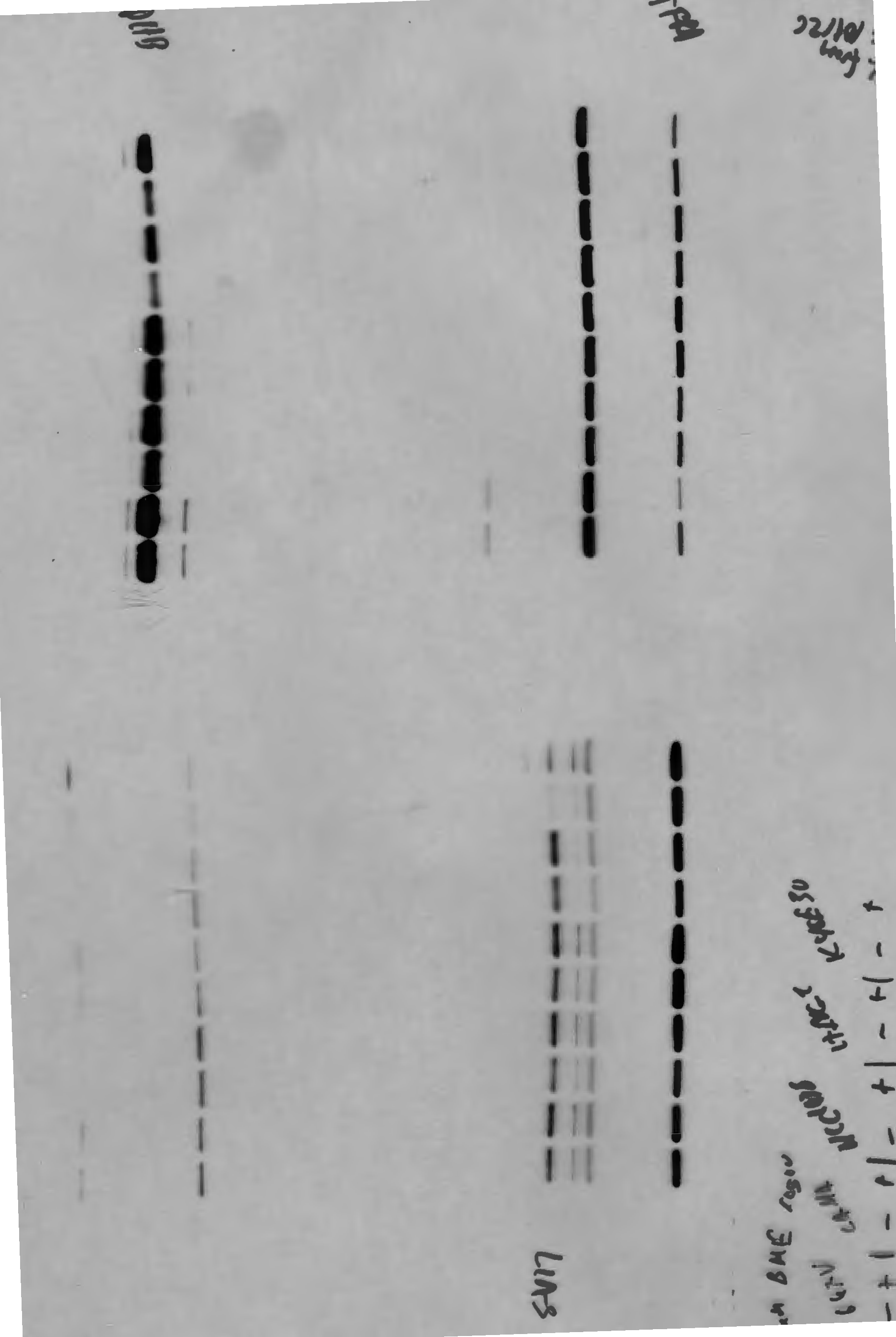
LIAS

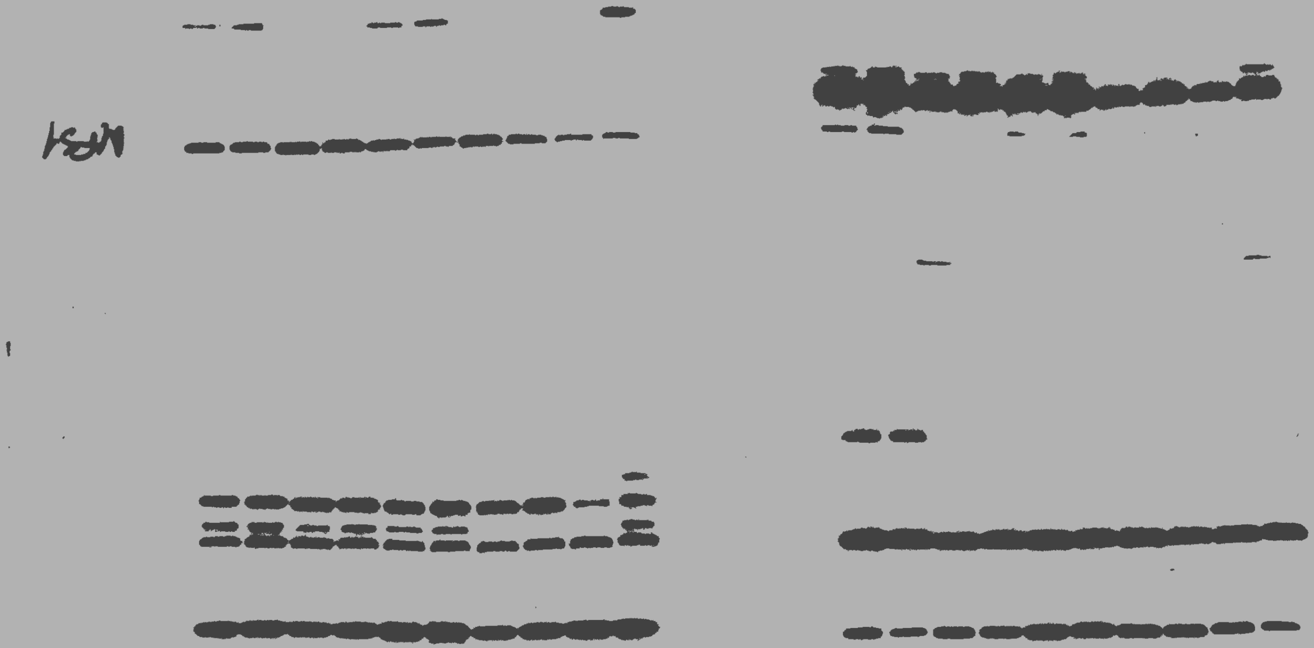

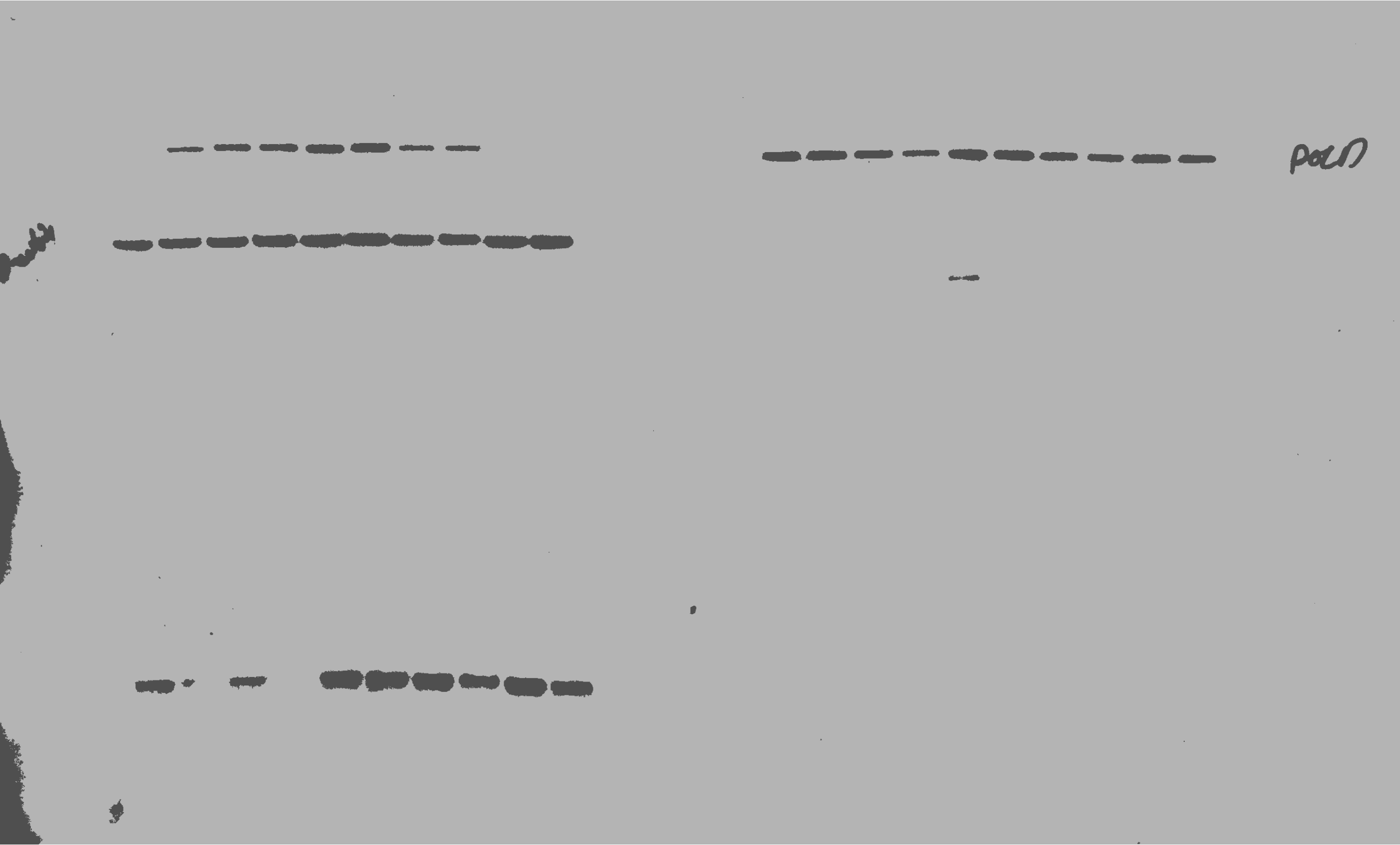

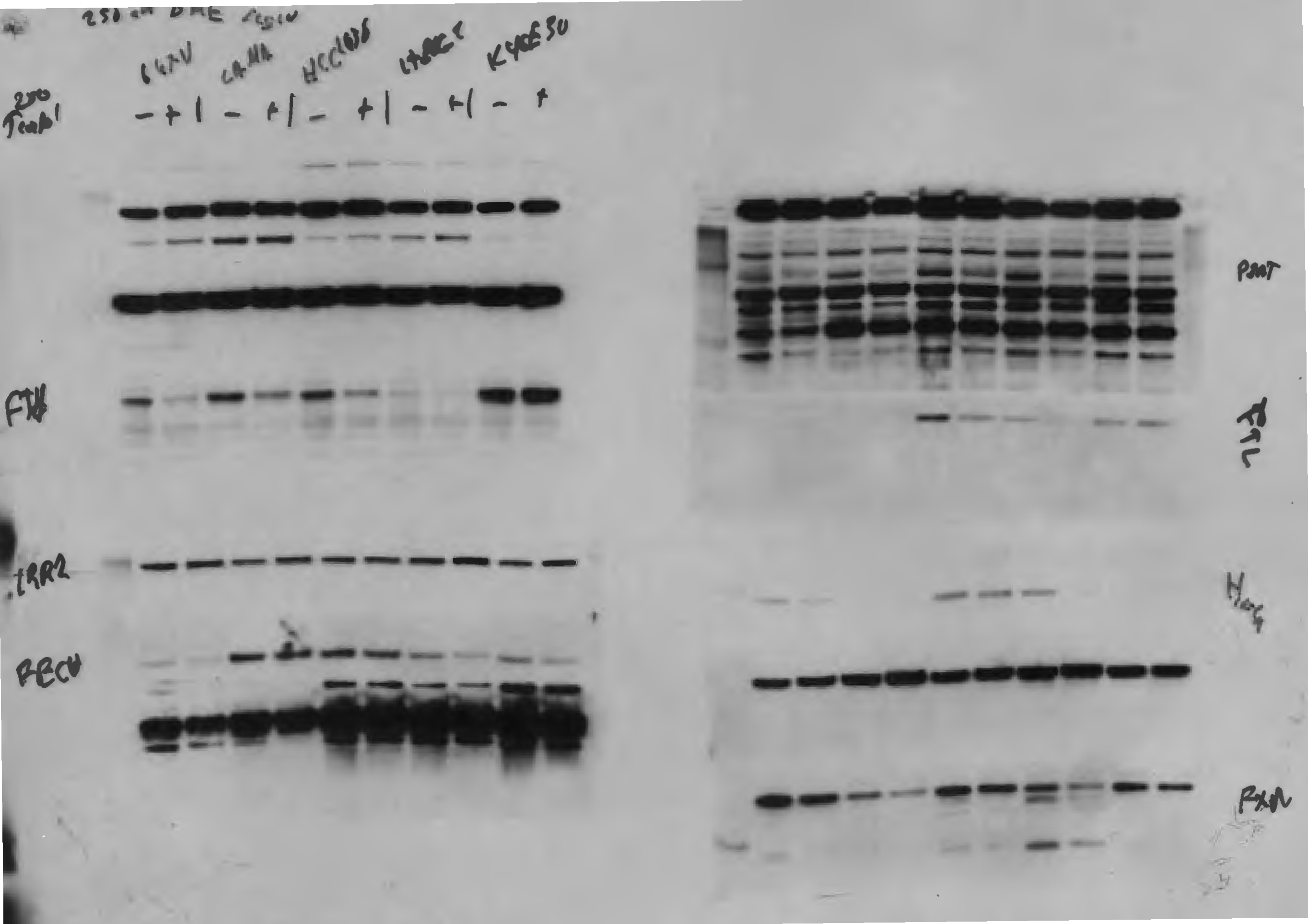
KYSE30
- + | - + | - + | - + | - +
FTH
FTL
